# Comparing two federal financing strategies on penetration and sustainment of the adolescent community reinforcement approach for substance use disorders: protocol for a mixed-method study

**DOI:** 10.1186/s43058-022-00298-y

**Published:** 2022-05-13

**Authors:** Alex R. Dopp, Sarah B. Hunter, Mark D. Godley, Chau Pham, Bing Han, Rosanna Smart, Jonathan Cantor, Beau Kilmer, Grace Hindmarch, Isabelle González, Lora L. Passetti, Kelli L. Wright, Gregory A. Aarons, Jonathan Purtle

**Affiliations:** 1 RAND Corporation, 1776 Main Street, Santa Monica, CA 90401 USA; 2 Chestnut Health Systems, 448 Wylie Drive, Normal, IL 61761 USA; 3 Department of Research and Evaluation, Division of Biostatistics Research, Kaiser Permanente Southern California, 100 South Los Robles Avenue 2nd Floor, Pasadena, CA 91101 USA; 4 RAND Corporation, 1200 South Hayes Street, Arlington, VA 22202 USA; 5 Department of Psychiatry, 9500 Gilman Dr. (0812), University of California San Diego, La Jolla, CA 92093 USA; 6 UC San Diego Altman Clinical and Translational Research Institute Dissemination and Implementation Science Center, La Jolla, CA 92093 USA; 7 Department of Public Health Policy & Management and Global Center for Implementation Science, New York University School of Global Public Health, 708 Broadway, New York, NY 10003 USA

**Keywords:** Adolescent substance use, Substance use disorder treatment, Evidence-based practices, A-CRA, Behavioral health service systems, Financing strategies, Implementation, Sustainment, Public finance, Policy

## Abstract

**Background:**

Sustained, widespread availability of evidence-based practices (EBPs) is essential to address the public health and societal impacts of adolescent substance use disorders (SUD). There remains a particularly significant need to identify effective financing strategies, which secure and direct financial resources to support the costs associated with EBP implementation and sustainment. This protocol describes a new project comparing two types of U.S. federal grant mechanisms (i.e., a type of financing strategy), which supported the implementation of the Adolescent Community Reinforcement Approach (A-CRA) EBP for SUD, through either organization-focused or state-focused granting of funds. The Exploration-Preparation-Implementation-Sustainment (EPIS) framework will guide our study aims, hypotheses, and selection of measures.

**Method:**

We will employ a longitudinal, mixed-method (i.e., web surveys, semi-structured interviews, document review, focus groups, administrative data), quasi-experimental design to compare the grant types’ outcomes and examine theoretically informed mediators and moderators. Aim 1 will examine the proportion of eligible clinicians certified in A-CRA with adequate fidelity levels (i.e., penetration outcomes) at the end of grant funding. Aim 2 will examine the sustainment of A-CRA up to 5 years post-funding, using a 10-element composite measure of treatment delivery and supervision activities. We will integrate the new data collected from state-focused grant recipients (~85 organizations in 19 states) with previously collected data from organization-focused grant recipients (Hunter et al., Implement Sci 9:104, 2014) (82 organizations in 26 states) for analysis. We will also use sensitivity analyses to characterize the effects of observed and unobserved secular trends in our quasi-experimental design. Finally, aim 3 will use comparative case study methods (integrating diverse quantitative and qualitative measures) to identify and disseminate policy implications about the roles of state- and organization-focused federal grants in efforts to promote adolescent SUD EBP implementation and sustainment.

**Discussion:**

The proposed research will have direct, practical implications for behavioral health administrators, policymakers, implementation experts, and the public. It will offer new knowledge that can directly inform financing strategies to support large-scale, sustained EBP delivery in behavioral health—while advancing implementation science through the use of novel methods to study financing strategies and sustainment.

**Supplementary Information:**

The online version contains supplementary material available at 10.1186/s43058-022-00298-y.

Contributions to the literature

- The project will advance understanding of effective financing strategies to support the implementation and sustainment of evidence-based practices for the treatment of adolescent substance use disorders.
- This project will generate new knowledge about penetration and sustainment outcomes, and mechanisms (i.e., mediators and moderators), for two types of U.S. federal grants (state-focused vs. organization-focused) designed to promote evidence-based practice implementation.
- This study will provide an example of using a quasi-experimental, mixed-method design and sensitivity analyses to maximize confidence in study conclusions and to identify generalizable conclusions about financing evidence-based practices for policy making.

## Background

In 2020, 1.6 million 12–17-year-olds (6.3% of adolescents) in the USA met the criteria for substance use disorders (SUD)—yet less than one in nine received treatment [1]. Providing high-quality treatment to these youth could help mitigate the adverse consequences of SUDs, including accidents, disease, violence, and criminality, which often last into adulthood [2, 3] and have estimated annual costs of $740 billion [2, 3]. To reduce the public health and societal impacts of adolescent SUD, behavioral health service systems must increase the availability of evidence-based practices (EBPs) for SUD [4–8]. Closing the current research-practice gap will require a better understanding of various strategies that support EBP implementation, defined as the practice of EBPs in everyday service settings [9–12].

The most recent systematic review [7] found that well-established EBPs for adolescent SUDs share several features, including delivery in community settings and a developmentally tailored approach that addresses youth, family, and peer influences. One such EBP is the Adolescent Community Reinforcement Approach (A-CRA [13]), a 12–14-week behavioral treatment for adolescents and young adults that seeks to replace environmental factors supporting substance use with alternative activities and behaviors. A-CRA improved substance use, mental health, and social outcomes in four randomized clinical trials [7], and implementing A-CRA with adequate fidelity predicts comparable clinical outcomes in community settings [14–20]. This project focuses on A-CRA as an exemplar EBP for adolescent SUD that has been widely implemented with a common, yet poorly researched, implementation financing strategy: federal grants.

### A-CRA implementation and sustainment in SUD services

Implementation of EBPs like A-CRA is best understood as a complex, long-term process that requires specific knowledge, skills, and resources—resulting in considerable costs to implementing organizations [10–12, 21, 22]. Public service organizations, including SUD treatment providers, must navigate multilevel contextual influences across Exploration, Preparation, Implementation, and Sustainment phases (as outlined in the EPIS framework [23, 24]) to successfully use an EBP. Various implementation strategies help providers and organizations navigate these phases [25–27]; strategies for A-CRA implementation have largely focused on individual clinicians and supervisors, including didactic training, technology-assisted consultation, and certification in the competent use of model procedures. Individual-focused strategies are important, but rarely sufficient to achieve large-scale penetration, as measured by the proportion of potential providers in a service system using an EBP [28]—let alone sustainment, or continued use of that EBP after initial support for implementation ends [29–31]. Indeed, previous research found that clinicians generally had positive perceptions of A-CRA, but about half discontinued its use due to intra- and extra-organizational factors such as limited leadership support or unstable funding [32–34].

Additional strategies are needed to support A-CRA penetration and sustainment, both key implementation outcomes [23, 24, 28], given that the public health impact remains limited without both widespread *and* long-term EBP use [35]. In particular, financing and budget considerations are essential, but behavioral health administrators must often make decisions about financing EBPs without research-based guidance [8]. Recent research identified financing strategies [36] that help organizations secure and direct financial resources to support implementation and sustainment; examples include increased reimbursement [37], grant or contract funding [38], and earmarked taxes [39]. All strategies involve government or philanthropic funders, and most provide time-limited funding to promote the success of initial implementation [40–46]. Unfortunately, little is known about how financing strategies can be optimized to promote SUD EBP penetration and sustainment [36].

### Current project

Federal initiatives that support EBP implementation offer unique opportunities to identify and more fully understand the impact of financing strategies on a large scale [6, 47]. In one of the largest investments in EBP implementation to date, SAMHSA’s Center for Substance Abuse Treatment has offered two types of discretionary grants to promote A-CRA implementation: “organization-focused” grants directly to treatment organizations and “state-focused” grants to state substance use service agencies. With state-focused grants, SAMHSA sought to spread A-CRA to a great number of providers within awardee states and create state-level infrastructure to support A-CRA sustainment. These two grant initiatives provide a natural experiment to compare penetration and sustainment outcomes between different financing strategies in cases where the EBP (A-CRA) and other implementation strategies were the same.

**Fig. 1:**
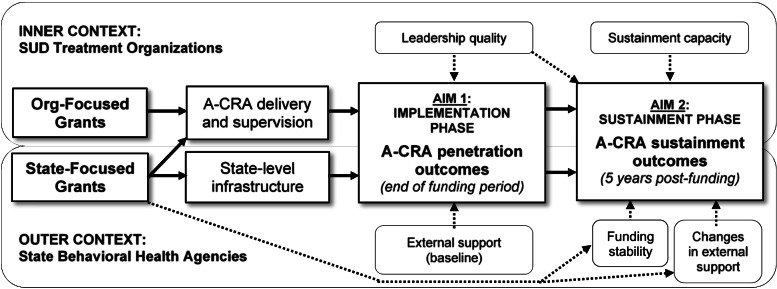
Pathways of proposed grant type effects on A-CRA implementation and sustainment outcomes. A-CRA = Adolescent Community Reinforcement Approach. SUD = substance use disorder. Predicted inner- and outer-context moderators and mediators of outcomes are shown by dotted arrows and round rectangles; mediators are distinguished by the additional dotted arrows showing their being affected by state-focused grants

The current project will use a longitudinal, mixed-method approach to gather data from state-focused grant recipients, aligned with previous work [48] that described implementation and sustainment among organization-focused grant recipients. We will integrate data from both projects to compare A-CRA outcomes between state-focused versus organization-focused grants. Figure 1 depicts the proposed natural experiment, illustrating how each grant type is thought to impact outcomes in the implementation and sustainment phases. Following the EPIS framework [23, 24], all grants targeted “inner” (intra-organizational) context factors and state-focused grants also targeted the “outer” (extra-organizational) context.

Our aims are to compare state-focused versus organization-focused SAMHSA grants on A-CRA (1) penetration, defined as rates of certification among eligible clinicians, and (2) sustainment, measured over a 5-year post-grant period. We hypothesize that state-focused grants will have higher rates of penetration and sustainment and that these effects will be moderated by inner- and outer-context factors that are associated with implementation success [49, 50]. We will also (3) identify policy implications for financing the implementation and sustainment of adolescent SUD EBPs through federal grants. Importantly, we do not propose to isolate the causal impact of grant type on outcomes; such conclusions are not possible without a fully randomized design and are unnecessary for answering our research questions. Rather, to increase our policy impact, researchers need to offer a wide range of rigorous, externally valid forms of evidence that policymakers can use [51–54].

## Method

### Research design

Aims 1 and 2 use a longitudinal, natural experiment design to compare A-CRA penetration and sustainment for state-focused grants (new data collection) versus organization-focused grants (collected previously [48]). Given that this is a natural experiment wherein we did not assign participants to grant types, we will conduct a sensitivity analysis that characterizes the influence of observed and unobserved secular trends on our estimates of grant type effects. Aim 3 will use a comparative case study design [55, 56] to identify policy implications for federal SUD service grants. Across aims, qualitative and quantitative data will be collected concurrently within a mixed-method approach [57].

In this protocol, we followed the Standards for Reporting Implementation Studies [58] (StaRI; see Additional file 1) for describing our project. All described procedures were reviewed and approved by the RAND Corporation IRB (Protocol #2020-N0887).

### Project context

As noted earlier, SAMHSA has used both organization-focused and state-focused discretionary grant strategies to fund EBP implementation (including A-CRA) for adolescent SUD. All grants were administered by SAMHSA’s Center for Substance Abuse Treatment [59], whose primary role was to provide funding, oversight, and national leadership to grantee organizations and states/territories/tribal entities (hereafter, “states”). SAMHSA funded (i) four cohorts of organization-focused grantees, awarded 2006–2010, across 26 states followed by (ii) four cohorts of state-focused grantees, awarded 2012–2017, across 22 states. Eight states had recipients of both grant mechanisms, so overall, 40 states were represented across cohorts.

In both initiatives, SAMHSA partnered with Chestnut Health Systems (CHS; an organization that conducts A-CRA training and research) to provide training and certification for A-CRA clinicians and supervisors. CHS provided a standardized, 2.5-day training for all organization-focused grantees and most clinicians in state-focused grantees’ states—although some received training from certified A-CRA supervisors. In every case, CHS still oversaw A-CRA clinician and supervisor certification. Certification activities required each trainee to demonstrate competency in delivering A-CRA treatment and/or supervision procedures, depending on their role. Specifically, clinicians and supervisors participated in twice monthly, group-format coaching calls with CHS expert consultants; they also submitted audio-recorded A-CRA therapy or supervision sessions, respectively, and their consultant provided numeric ratings and written feedback [60–62]. Typically, the certification process lasted from 6 months to a year.

Next, we describe the two grant types in more detail. We follow recommendations for specifying implementation strategies in terms of actors, actions, action targets, temporality, dose, outcomes, and justification [27, 36].

#### Organization-focused grants

SAMHSA awarded these grants directly to treatment organizations. Grantees received ~$900,000 across a 3-year period to support A-CRA implementation. These grants targeted the EPIS inner context by paying for clinicians delivering A-CRA, supervisors providing A-CRA supervision, and related activities such as evaluation (see https://externallinks.samhsa.gov/grants/2009/ti_09_002.dhtml to view a representative request for proposals). Our prior work showed that grantees generally had initial success in implementing A-CRA with fidelity and reducing youth substance use, but many did not sustain A-CRA post-funding [32–34].

#### State-focused grants

SAMHSA awarded these grants to state agencies that administer publicly funded SUD services. These were ~$3–4 million, 3- to 4-year awards (sometimes extended to 6 years) that could support the implementation of several EBPs; most funded states selected A-CRA. The grants targeted EPIS outer context factors [4, 43, 63]—one third of the grant funds paid for states to develop EBP-focused infrastructure like funding, training, developing policies, and promoting treatment organizations’ capacity to deliver youth SUD treatment. State agencies proposed up to six “dissemination site” organizations that implemented A-CRA first; those sites received funds similar in amount and purpose to those provided to organization-focused grantees, thus still addressing inner context factors. See https://www.samhsa.gov/grants/grant-announcements/ti-17-002 to view a representative request for proposals. The outcomes of state-focused grants have not been evaluated.

### Participants

Participants will include state SUD agency administrators, clinicians, and supervisors from treatment organizations that implemented A-CRA (in both state-focused and organization-focused grants), and other state and federal agency administrators whose work is relevant to youth SUD treatment.

#### State SUD service agencies

We will recruit state agency administrators from each state that received a state-focused grant. To be eligible, administrators must be currently or recently employed in a leadership position that involved administration of a SAMHSA state-focused grant from the past 5 years; we will seek to interview one administrator per state, but will permit group interviews when appropriate. Three states’ grants ended more than 5 years ago, so SUD service agency administrators from 19 states are eligible for participation.

#### Treatment organizations

In our previous project, we collected semi-structured interview and survey data from 169 clinicians and supervisors at 78 treatment organizations (across 27 states) that received SAMHSA organization-focused grants [33, 34]. The current project involves collecting similar data from treatment organizations that received support from their state agency to implement A-CRA as part of a state-focused grant, including the dissemination sites and any other organizations that implemented A-CRA. To be included in the current project, organizations must be located in a state where less than 5 years have passed since their state-focused grant ended.

Given the larger scope of state-focused grants, we will not collect data from every treatment organization involved (across the 19 eligible states, CHS records indicate that staff from 282 organizations received A-CRA training). Instead, we used a three-step sampling procedure to select up to five organizations per state from which data will be collected; this approach kept data collection feasible and ensured that organizations from highly successful states were not overrepresented. First, we identified five organizations (in four states) that received training through a state-focused grant while their organization-focused grant was still active, and excluded them due to the extent of confounding. Second, we selected up to three dissemination sites to ensure we included some organizations targeted directly by state-focused grants without overrepresenting the organizations most likely to implement A-CRA successfully; we randomly selected three dissemination sites if a state had more. Third, we randomly selected from the remaining treatment organizations in each state until we reached a maximum of five per state. These procedures selected 91 organizations (*M* = 4.78 per state) from which we will recruit. Chi-squared tests using CHS administrative data indicated that clinicians and supervisors from the selected organizations were no more or less likely to be A-CRA certified (*χ*^2^_(1)_ = 3.416, *p* = .065) or to have left the organization (χ^2^_(1)_ = 2.480, *p* = .115) than those not selected.

Individuals currently or recently employed as a clinician or clinical supervisor responsible for adolescent SUD treatment will be eligible to participate (with a preference for individuals knowledgeable about A-CRA implementation). We kept eligibility criteria for clinicians and supervisors consistent with the previous organization-focused sample [34], which will allow us to combine the data for analyses. The previous sample [34] had an average of 2.17 participants per organization (41% clinicians, 59% supervisors), and we will aim for a similar breakdown of participants. A maximum of 197 clinician and supervisor participants may be included in the state-focused grant sample. Also, in cases where an organization no longer operates adolescent SUD treatment services, we may instead collect data from program directors or other administrative staff with knowledge about the organization’s SUD treatment services.

#### Federal and state agency administrators

Near the end of the project, we will ask state agency administrator participants to nominate administrators in other state and federal agencies with which they partner to address youth SUD services. We will invite those nominees to participate in focus groups, supplementing with our own nominations as needed to ensure broad representation of relevant agencies (e.g., SAMHSA, Centers for Medicare and Medicaid Services, Health Resources and Services Administration). Agency administrators (i.e., appointed policymakers) are the most relevant audience for discussing the implications of our findings, as they (not elected policymakers) are responsible for setting most grants administration policies.

#### Recruitment

The RAND Survey Research Group—a center specializing in quantitative and qualitative primary data collection—will be responsible for all interview and survey recruitment activities. Recruiters will contact eligible individuals using contact information from CHS, which maintains a list of SAMHSA grantees and has pre-established working relationships with the state SUD agency administrators and treatment organizations through their A-CRA training and certification activities. CHS will support all recruitment activities and help to resolve challenges that arise.

Recruiters and interviewers will use multiple methods (i.e., mail, phone, e-mail) to connect with participants and remind them about data collection opportunities and timelines, consistent with effective survey methods [64]. We will reach out to state agency administrators first, to ensure they are aware of the project and can provide input if needed, before contacting clinicians and supervisors in the same state. Interviewers will collect comprehensive contact information for follow-up. Individuals who decline participation in a given wave of data collection will still be invited in future waves, as long as they remain eligible.

In the last year of the project, the PI (first author) will provide state SUD agency administrators with information about focus groups, invite them to attend, and solicit their nominations of other (state and federal) administrators to invite. The PI will then extend email and phone invitations for the focus groups to all nominated administrators, emphasizing the relevance of the project to their agency’s mission.

For each interview, survey, and focus group, we will offer compensation of $25 per activity completed. However, we anticipate some participants will decline compensation due to organizational restrictions or viewing participation as part of their professional role.

### Data sources and collection procedures

We will collect four waves of quantitative and qualitative data from state-focused grantees, to be combined with administrative records from CHS and previously collected data from organization-focused grantees. Table 1 details the planned data collection schedule. In each wave, we will collect data (semi-structured interviews, surveys, document review) from state agency representatives and clinicians/supervisors. Treatment organizations that report no longer delivering A-CRA will complete the ongoing wave of data collection, but will only participate in subsequent waves if they begin delivering A-CRA again and thus have new information to share (we will verify at the beginning of each wave). Focus groups with state and federal agency administrators will take place in the final year and so are not included in Table 1.

**Table 1:** Waves of planned data collection for state-focused grant cohorts

Grant cohort (years)	Target # of organizations	Expected # of respondents	Data collection waves: years since funding ended			
			Wave 1 (2021–2022)	Wave 2 (2022–2023)	Wave 3 (2023–2024)	Wave 4 (2024–2025)
(1) 2013–2017	7 states 37 organizations	7 state agency administrators 33 clinicians 47 supervisors	4 years Post-funding	^a^5 years post-funding	*NA*	*NA*
(2) 2012–2018	7 states 33 organizations	7 state agency administrators 29 clinicians 42 supervisors	3 years Post-funding	^a^4 years post-funding	^a^5 years Post-funding	*NA*
(3) 2017–2021	5 states 21 organizations	5 state agency administrators 19 clinicians 27 supervisors	0 years Post-funding	^a^1 year post-funding	^a^2 years Post-funding	^a^3 years Post-funding

In each data collection wave, we will collect semi-structured interviews from all eligible participants, web-based surveys from eligible clinicians and supervisors, and grant-related documents for review from eligible state agency administrators

*NA* not applicable (i.e., data are not collected because the data collection wave falls more than five years post-funding)

^a^ Treatment organizations that reported not sustaining the Adolescent Community Reinforcement Approach (A-CRA) in the previous wave will not be eligible unless they report having restarted A-CRA delivery since the previous wave

We will collect data via secure methods (telephone for interviews, Confirmit for surveys, Zoom for Government for focus groups). We will obtain informed consent for each activity, and all data collection will be voluntary. We will not collect personally identifiable information; we will de-identify each participant’s data and assign them a unique identification number.

Next, we describe each data collection activity, followed by the details of specific measures to be collected. In addition, Tables 2 and 3 provide summaries of the data collection activities used to evaluate penetration and sustainment outcomes, respectively.

**Table 2:** Data collection activities for comparing A-CRA penetration outcomes between grant types

Activity	Measures	Participants	Time	Compensation	Relevant Aims^a^
Administrative data from CHS		Clinicians, supervisors^b^	n/a	n/a	
	Proportion certified				Aim 1a, 1b, 3
	Descriptive data about fidelity scores				Aims 1a, 1b
Web survey (Wave 1)			30 min	$25	Aims 1b, 3
	External support [65]	Supervisors^b^			
	Leadership support (ILS [66])	Clinicians^b^			
Semi-structured interviews (Wave 1)			45 min	$25	Aims 1a, 1b, 3
	Organization-reported barriers/ facilitators of A-CRA implementation	Clinicians, supervisors^b^			
	State agency-reported barriers/ facilitators of A-CRA implementation	State behavioral health agency administrators			
Document review	A-CRA implementation-related documents	State behavioral health agency administrators	as needed	n/a	Aims 1a, 1b, 3

*A-CRA* Adolescent Community Reinforcement Approach, *CHS* Chestnut Health Systems, *ILS* Implementation Leadership Scale

^a^See Fig. 1 for details of the project aims and how they relate to each other

^b^Data collected from organization-focused grantees in the previous project will be included in the analysis

**Table 3:** Data collection activities for comparing A-CRA sustainment outcomes between grant types

Activity	Measures	Participants	Time	Compensation	Relevant Aims^a^
Semi-structured interviews (all waves)			45 min	$25	Aims 2a, 2b, 3
	*A-CRA supervisor knowledge	Supervisors^b^			
	*Number of certified therapists	Supervisors^b^			
	*Number of certified supervisors	Supervisors^b^			
	*A-CRA usage	Supervisors^b^			
	*A-CRA dosage	Clinicians^b^			
	*Supervision frequency	Clinicians^b^ Clinicians^b^			
	*Supervision content				
	*Use of session review	Clinicians^b^			
	*A-CRA training quality	Supervisors^b^			
	Organization-reported barriers/ facilitators of A-CRA sustainment	Clinicians, supervisors^b^			
	State agency-reported barriers/ facilitators of A-CRA sustainment	State behavioral health agency administrators			
	Impact of COVID-19 on A-CRA sustainment	Clinicians, supervisors, state behavioral health agency administrators			
Web survey (all waves)			30 min	$25	Aims 2a, 2b, 3
	*A-CRA clinician knowledge	Clinicians^b^			
	Sustainment capacities (PSAT) [67]	Clinicians, supervisors^b^			
	External support [65]	Supervisors^b^			
	Leadership support (ILS [66])	Clinicians^b^			
Document review	A-CRA sustainment-related documents	State behavioral health agency administrators	as needed	n/a	Aims 2a, 2b, 3

*A-CRA* Adolescent Community Reinforcement Approach, *ILS* Implementation Leadership Scale, *PSAT* Program Sustainability Assessment Tool

^*^An element that is used to calculate the 10-element A-CRA sustainment outcome measure [68]

^a^See Fig. 1 for details of the project aims and how they relate to each other

^b^Data collected from organization-focused grantees in the previous project will be included in the analysis

#### A-CRA certification records

In year 1, CHS will create a database of penetration outcomes for all sampled treatment organizations. The database will detail the certification levels achieved during the grant period by each clinician and supervisor. Given the link between A-CRA fidelity and clinical outcomes [14, 16, 17], CHS will include each certified individuals’ fidelity data from sessions rated by trained CHS staff.

#### Semi-structured interviews

The interview protocols will use a combination of open-ended questions and focused, standard probes [69]. Protocols will be tailored to each participants’ role, and for treatment organizations, whether the organization is still delivering A-CRA; see Additional file 2 for copies of all Wave 1 protocols. The interviews will gather information about the state- and/or organization-level approaches to disseminating A-CRA, how the implementation of A-CRA was supported during the funding period (in the first interview only), how sustainment of A-CRA has been supported or discontinued post-funding, and sustainability planning. State administrators will also be asked about the infrastructure (e.g., training, policy development) developed during their grant period and plans to sustain those activities. Clinicians and supervisors will also be asked questions from our 10-element composite measure of A-CRA sustainment [68] (specified in Table 3). For organizations that did not sustain A-CRA, some questions will be anchored to the 6-month period prior to A-CRA discontinuation to allow comparable information to be collected. Initial interviews are anticipated to take 45 min, and subsequent interviews will be shortened (focusing on changes since the last wave) to approximately 30 min. We plan to audio-record and transcribe interviews.

#### Previously collected interviews

As noted previously, we collected semi-structured interviews from 169 clinicians and supervisors at organization-focused grantee organizations [32, 34]. The interviews were collected across three waves up to 5 years post-funding. These data will be combined with interview data from state-focused grantees for the planned analyses. As much as possible, the interview protocols for the current project were adapted from those used in our prior work, to ensure comparability of findings. We also grounded the protocols in the EPIS framework, to ensure we consistently ask about multi-level influences on A-CRA outcomes while distinguishing between implementation and sustainment phases.

#### Clinician surveys

Following each interview, clinicians and supervisors will be sent a web-based survey that collects standardized measures of multi-level contextual influences on A-CRA penetration and sustainment (again grounded in EPIS), one A-CRA sustainment element (see Table 3), potential moderators and mediators (see Fig. 1), and other descriptive information. As with interviews, the survey items will be tailored to the participant’s role and A-CRA sustainment status; again, some non-sustainer questions will be anchored to 6 months prior to discontinuation. The surveys are designed to take ~30 min to complete. See Additional file 3 for all Wave 1 survey items.

#### Previously collected surveys

Again, we will combine the newly collected survey data with previously collected waves of survey data from the clinicians and supervisors in our previous project [32, 34]. The measures collected in this project will be a subset of those used in our previous surveys to ensure comparability. However, we also supplemented the survey items with a few recently published measures that capture relevant descriptive variables (e.g., financial status, self-reported sustainment).

#### Document review

We will ask state administrators to identify and share documentation of state-focused grant activities for qualitative review. This method can provide useful insights into complex systems-level processes (like A-CRA implementation and sustainment) when interpreted alongside other qualitative and quantitative data [38, 70]. We will solicit documents related to the presence of, the extent of (e.g., number/frequency), and plans to maintain various state EBP infrastructure components listed in the Requests for Proposals from SAMHSA state-focused grants. Examples include grant progress reports and related data, contracts with A-CRA treatment organizations, and strategic planning tools. In interviews, state administrators will identify documents to be shared; the PI will follow up via email or phone to establish any needed confidentiality/data sharing agreements and secure the documents.

#### Focus groups with federal and state agency administrators

For identifying policy implications (aim 3), where it is important to distinguish among experts’ differing and nuanced viewpoints, focus groups are an ideal data collection method [69]. Following the final interview wave, we will hold virtual focus groups (via video conference) with state SUD agency administrators and other (state and federal) administrators. We will arrange three state-level groups, each grouping together states that had similar experiences with A-CRA; the federal administrator group will be separate. Before each group, we will ask attendees to review a policy brief [71, 72] that we will create outlining (a) key financing issues motivating this project and (b) preliminary findings. In each 45-min group, the focus group leaders (a combination of the first three authors) will review the policy brief, then engage attendees in a discussion of implications for integrating the findings into EBP-focused federal grant policies. We will take detailed field notes plus audio-record and transcribe focus groups.

### Measures

#### Grant type

We will determine participants’ involvement in SAMHSA grants based on CHS records. We will verify that determination during the consent process and with questions at the start of each interview.

#### Outcomes

As recommended for evaluating implementation strategies [28, 73, 74], we will compare the impacts of organization-focused versus state-focused grants using key implementation outcomes [23, 24, 28] for A-CRA: penetration (aim 1) and sustainment (aim 2).

##### Penetration

We define penetration as the proportion of providers (i.e., clinicians and supervisors) in a service system that were certified in A-CRA during active implementation. We will use CHS administrative records to define and measure penetration (see Table 2). Potential providers (i.e., the denominator) will include all individuals eligible for training and/or certification activities at a treatment organization during the state-focused grant period; the grant funding period will be treated as active implementation. Certification status will be based on CHS’s standard definitions for different levels and types of A-CRA certification.

A-CRA clinician certification is based on proficient demonstration of A-CRA procedures, which are 19 clinical techniques or activities used by clinicians (e.g., functional analysis of substance use, problem-solving, communication skills); first-level certification indicates proficiency in nine basic procedures, and full certification indicates proficiency in every procedure [13]. Further certification for proficiency with transition-age youth (ages 18–25) is also available and considers two additional procedures. A-CRA supervisor certification is based on proficient demonstration of supervision-specific skills and knowledge, as well as the ability to accurately and reliably rate A-CRA sessions. We will construct several penetration variables, representing the percent of participants achieving any certification as well as first-level, full, transition-age youth, or supervisor certifications.

We will also verify that each certified individual achieved adequate A-CRA fidelity, defined as an average competence rating of ≥3 out of 5 across the relevant activities for a given A-CRA certification (i.e., procedures in session recordings, supervisors’ competence in rating sessions) following past research [14, 16, 17]. We will calculate average fidelity scores that contributed to each individual’s clinical (first-level, full, transition-age) and/or supervisor certification; the same ratings can contribute to all clinical certifications, so these cannot be calculated separately. We will also calculate descriptive measures for each certification type characterizing time to certification, number of session recordings reviewed, and procedure-specific scores.

##### Sustainment

We define A-CRA sustainment over the 5-year post-grant period using a 10-element measure developed in our previous research [68], which captures the ongoing quality of treatment delivery, staffing, and supervision (see Table 3). These elements are pragmatic [75] to collect from treatment organizations and center on program-level processes, rather than clinical outcomes, as recommended for sustainment measures [76].

The A-CRA sustainment elements are collected via interviews (5 from supervisors, 4 from clinicians) except for the A-CRA knowledge test for clinicians, which is collected through the web-based survey to match how the test is typically administered. Each element will be assessed as follows: (1) clinician knowledge via a 10-item multiple choice survey, (2) supervisor knowledge using a 15-item true/false questionnaire, presence of certified (3) clinicians(s) and (4) supervisor(s) is self-reported and verified with CHS records, (5) usage by the self-reported percent of eligible youth receiving A-CRA in the past six months, (6) dosage by clinicians’ self-report of the number of sessions delivered (12–14 prescribed), clinicians also report whether clinical supervision (7) occurs biweekly, (8) involves six key activities, and (9) includes a review of recorded sessions, and (10) quality of A-CRA training plans submitted by supervisors will be rated by CHS staff (i.e., percentage of expected training components included). A composite A-CRA sustainment score is created by normalizing each element (on a 0 to 1 scale, averaged across all ratings from the same treatment organization), then summing the normalized scores (i.e., composite scores range from 0 [no] to 10 [complete] sustainment) [68].

We will also measure clinicians’ and supervisors’ global ratings of A-CRA sustainment using the newly published, three-item Provider Report of Sustainment Scale [77]. This offers a point of comparison for our primary sustainment measure.

#### Mechanisms

We will also examine components of the mechanisms [78, 79] by which organization-focus and state-focused grants influence A-CRA implementation outcomes. This will include testing for mediation of sustainment outcomes and moderation of both penetration and sustainment outcomes. All variables will be captured through the web-based surveys.

##### Penetration

We will test two a priori moderators of A-CRA penetration outcomes: outer-context reimbursement for services (“external support”), and inner-context leadership support for implementation [49, 80, 81]. We will capture external support using a series of survey items designed for adolescent treatment providers [65] that ask supervisors to report what percentage of treatment for youth is reimbursed from various sources (e.g., Medicaid, juvenile justice contracts); we will consider both the total proportion of youth treatment receiving external support and patterns of funding as potential moderators. We will measure leadership support using the 12-item Implementation Leadership Scale [66], which measures clinician perceptions of their supervisors’ leadership qualities (proactive, knowledgeable, supportive, perseverant) toward EBPs using a five-point scale (0=“not at all,” 4=“very great extent”). We will calculate an average score across all items. Note that our previous work examined leadership support toward EBPs in general among organization-focused grantees [33]; we will collect the same measure again for comparability, but because we did not previously find leadership support predicted sustainment outcomes, we will administer a second version of the Implementation Leadership Scale to explore the role of A-CRA-specific support in our sample.

##### Sustainment

We expect that the moderating effect of leadership [66] will hold true for A-CRA sustainment. We also expect changes in external support [65] during the sustainment period to function as a mediator variable, because state-focused grants might influence the availability of funding. Furthermore, we anticipate additional moderators and mediators based on the eight domains of EBP sustainment capacity identified in the Public Health Sustainability Framework [50]. We will measure these domains with the 40-item Program Sustainability Assessment Tool [67]; ratings use a seven-point scale (0=“to little or no extent,” 7=“to a very great extent”) and are averaged to produce an overall sustainment capacity score and domain subscale scores. We expect that differences in sustainment between state-focused and organization-focused grants will be mediated by increases in the “funding stability” domain over time and moderated by overall sustainment capacity.

#### Descriptive measures of A-CRA barriers and facilitators

We will collect various other measures that characterize the states and organizations implementing A-CRA. Broadly, the measures align with our previous project while capturing important constructs from all major EPIS domains: innovation, inner context, outer context, and bridging factors that link outer and inner contexts. The measures include both structured, closed-response questions and open-ended exploration of barriers and facilitators. We briefly describe specific topics asked about here; for details, see Additional files 2 and 3.

*Innovation factor* measures capture participants’ perceptions of and attitudes toward the A-CRA model (e.g., complexity, relative advantage). *Inner context* measures describe the treatment organizations delivering A-CRA, including their focus of services, staffing (e.g., number of therapists and supervisors, turnover rates), adolescent SUD treatment capacity (e.g., number of youth served, length of stay), and organizational plans to spread and sustain A-CRA (or not). Sustainment capacities captured by the Program Sustainability Assessment Tool [67] will also be considered here, as will a recently published measure of the organization’s financial climate [82]. *Outer context* measures describe extra-organizational factors, including sources of adolescent SUD treatment referrals, formal partnerships between organizations, and policies that affected A-CRA penetration and sustainment. The inclusion of state agency administrators’ views will greatly expand our understanding of outer context factors. Finally, we will collect detailed descriptions of *bridging factors* by asking state agency administrators about A-CRA infrastructure developed in their state. These questions will explore how each state tailored [83–85] their grant-funded efforts to local contexts and allow us to examine if certain infrastructure components were associated with A-CRA penetration and sustainment.

#### COVID-19 impact

Our project timeline coincides with the COVID-19 pandemic, so we must consider the potential impacts on key project outcomes. Actions to prevent the spread of COVID-19, such as telehealth service delivery [86, 87], and budget shortfalls due to economic constriction [88] are transforming U.S. behavioral health services [89]. We expect statistical controls or corrections (i.e., sensitivity analyses) will likely be insufficient to account for the far-reaching and inter-related impacts of the pandemic on employment, treatment availability, substance use, and funding priorities [90]. Instead, we will gather mixed-method data to help us understand the contextual influences of COVID-19 on A-CRA penetration and sustainment. In Wave 1 interviews, we will ask how the COVID-19 pandemic has impacted A-CRA services, barriers to penetration and sustainment, and strategies used to address those barriers. The Wave 1 survey will include additional items about how COVID-19 impacted A-CRA services. These questions may be repeated or modified in later waves, depending on the course of the pandemic.

#### Measures for sensitivity analyses

To help understand trends that may be confounded with grant type in our quasi-experimental design, we will conduct a series of sensitivity analyses. We will account for non-secular trends by creating a series of binary variables (using CHS records) that indicate which years each state had active SAMHSA state-focused grant support. To account for secular trends, we will collect non-equivalent dependent variables [91, 92] from publicly available data about the participating treatment organizations. Non-equivalent dependent variables are uniquely useful when comparing groups exposed to different policy interventions in non-overlapping time periods with no control groups available. These variables capture an observed secular trend because they (1) *are not* expected to be influenced by the predictor variable of interest (i.e., grant type) but (2) *are* expected to be influenced by factors that provide an alternate explanation for observed differences (i.e., other factors that could promote EBP implementation at SUD treatment organizations).

The non-equivalent dependent variable design is strengthened by the inclusion of multiple variables, each with controlled covariates. At a minimum, we plan to examine variables representing organization-level use of medication treatments for opioid use disorder (e.g., methadone detoxification/maintenance, buprenorphine use) as non-equivalent dependent variables; each represents a discrete EBP that could be influenced (as with A-CRA) by efforts to improve substance use care, but not by A-CRA-focused grants. We will collect each variable from the publicly available National Survey of Substance Abuse Treatment Services (N-SSATS) [93], an annual census of SUD treatment organizations (since 2000; response rates ≥90%), and control for increases in state-level (a) opioid-related overdose death rates among adults using CDC cause of death data [94] and (b) SAMHSA grant funding amounts for opioid-specific treatment services [95]. We will also continue exploring N-SSATS data to identify other candidate non-equivalent dependent variables, each of which would require its own set of covariates. We will collect all variables for every year relevant to our analyses; i.e., 2009 (when the first organization-focused grants ended) through 2025 (our final year of data collection).

#### Policy implications

For aim 3, we will explore government agencies’ adoption of financing strategies rather than treatment organizations’ adoption of A-CRA. We will develop focus group facilitation guides based on a recent adaptation [96] of the Consolidated Framework for Implementation Research (CFIR [97, 98]) for policy implementation. Questions will be tailored to the characteristics of attendees, soliciting views about the use of organization-focused versus state-focused grants to support EBP penetration and sustainment; usefulness of states’ and organizations’ grant-funded activities; and barriers and facilitators for high-priority changes—related to characteristics of policy changes, policymakers, inner-context government agencies, and outer-context public opinion and political climates. Focus group discussions will be guided by our findings from aims 1 and 2.

### Analysis plan

Our analytic approach is grounded in mixed methods [57], combining quantitative data (standardized interview and survey items) and qualitative data (e.g., open-ended interview questions, document review, focus groups) to gain higher-level insights beyond what either approach provides in isolation. Qualitative data will deepen our understanding of quantitative findings in aims 1 and 2, representing a QUAN + Qual mixed-method design; we will rely more heavily on the qualitative data for aim 3, taking a QUAL + Quan approach [57].

Aim 1 only requires CHS administrative data, qualitative data collected in year 1, and non-equivalent dependent variables, so we plan to complete data analysis by the end of year 2. For aim 2, we will conduct waves of data collection across 4 years (see Table 1), maintaining progress on data management, document review, and non-equivalent dependent variable collection during low-effort recruitment periods. Aim 3 will use data from the main waves, which we will separate and incorporate into comparative case studies in years 4 and 5—along with the focus group data.

#### Qualitative analysis of interviews

For aims 1 and 2, we will follow best practices for conventional content analysis [99], using Microsoft Excel and NVivo qualitative software to organize and analyze the new interview transcripts. The first two authors will first read and sort sections of the transcripts into common themes. We will use the previous project’s themes as a starting point [32], but allow for iterative refinement through the identification of emergent themes and comparison to concepts represented in EPIS. Once 50% of interview data are collected for a wave, we will develop a codebook with theme descriptions, definitional criteria, and exemplars [100], then train research assistants to code the remaining transcripts. After fully coding 10% of the interviews, we will review the coding and discuss discrepancies to finalize the codebook. Interviewing staff will also provide input into coding. If our codebook has major changes from the previous project, we will re-code prior qualitative data with the new codes.

#### Quantitative data analysis

We will integrate the data collected in this project with our prior data [33, 34, 48]. To compare state-focused vs. organization-focused grants, we will fit hierarchical models for penetration outcomes (aim 1) and longitudinal pattern-mixture models [101, 102] for sustainment outcomes (aim 2). Variables in the model come from two levels, state and organization, with ratings from multiple respondents averaged to the organization level (as in the previous project). In aim 2, each organization will have up to four repeated measurements, allowing us to fit organization-specific trajectories. Longitudinal pattern-mixture models do not require fully synchronized measurements across organizations, which is suitable for our analyses. We will have missing data at some measurement points due to nonresponse and the timing of data collection (see Table 1), so we will apply multiple imputation [103] and data reweighting [104] to handle nonresponse. If organizations differ significantly on observable characteristics and baseline outcomes, the model fitting process will use propensity scores [105] to adjust for covariate balancing. These models can be readily fit in the general statistical software package SAS 9 PROC MIXED. After fitting all models, we will apply the step-up method [106] to adjust for multiple comparisons and maintain a type I error rate <0.05 for each aim. For analyses of mechanisms, we will examine moderators by adding interaction terms to the models and will use structural equation models to examine indirect and direct mediation effects; for aim 2, the moderator and mediator variables are time-varying. Additional file 4 presents detailed equations for each analysis in aims 1 and 2.

We hypothesize that treatment organizations implementing A-CRA under state-focused grants will have higher penetration rates compared to organization-focused grantees, with differences between grant types moderated by external support and leadership quality. Similarly, we predict that state-focused grants will show greater A-CRA sustainment by treatment organizations across 5 years post-funding, with moderation by leadership quality and sustainment capacity mediation by changes in external support and funding stability over time.

#### Statistical power

For aim 1, under regular settings (power>80%, 2-sided *p*-value<.05), we can detect a medium effect size of 0.46 times standard deviation (*SD*) on penetration when the covariates have no effect, and a medium effect size of 0.40 times *SD* when the covariates explain 20% of the variance in penetration. The power for moderation analysis is weaker and more difficult to quantify; in a simplified setting of high versus low values, we can detect a large moderation effect size of 0.89 times *SD*.

Under the same settings for aim 2, if we suppose that across all patterns each treatment organization can provide two measurements over time, on average, and assume an intra-class correlation no greater than 0.40, we can detect a small standardized effect size of 0.34 times *SD*. Based on guidelines for mediation analysis [107], we can detect a moderately small mediation relationship (i.e., standardized *μ*^(0)^=0.26) by most methods drawing inference for the indirect effect, when the intra-class correlation is no bigger than 0.50. In a simplified setting of high vs. low values in a moderator, we can detect a medium moderation effect size of 0.69 times *SD*.

#### Sensitivity analyses

Sensitivity analyses will account for observed and unobserved secular trends, using the same models for penetration and sustainment. First, we will test non-equivalent-dependent variables (medication treatment variables) as *Y*^*N*^ outcomes, moderators during the grant funding period (aim 1a) or in each year of sustainment (aim 2a). We hypothesize grant type will not have significant effects on non-equivalent dependent variables, but if we find one, we will adjust the standardized effect size for the relevant outcome (penetration or sustainment) by subtracting out the effect size for the non-equivalent dependent variable. Second, we will repeat all primary analyses controlling for each organizations’ years of active funding (time fixed efforts), which could capture unobserved secular trends missed by our non-equivalent-dependent variables. If grant effects are reduced by controlling for time, we will use our qualitative data to help understand why.

#### Comparative case studies to identify policy implications

We will develop a policy brief summarizing our findings and use case study methods [55, 56] to integrate details about state-level infrastructure elements supporting A-CRA; and multi-level determinants of financing strategies’ use, identified through focus groups with state and federal agency leadership. Our comparative case study approach will treat the 19 state-focused grantee states as individual cases. This involves creating descriptive summaries of grant activities undertaken by each state to support A-CRA and lessons learned for executing these grants. Our case study reports will integrate quantitative and qualitative data from interviews, surveys, reviewed documents, and focus groups with multiple stakeholder types, consistent with rigorous case identification and analysis methods [69, 108].

We will begin the comparative case studies in year 4 by creating descriptive summaries of state infrastructure for A-CRA reported by state SUD agency administrators in their interviews and identified via review of documents (e.g., progress reports, contracts, sustainment plans). Document review [70] is a form of content analysis, in which the first author and research assistants will conduct a line-by-line review of each document, extracting findings into a state-specific matrix with analysis codes (rows) for each document (columns) [38]. Qualitative interview themes will help interpret the documents. We will compare and contrast the infrastructure elements in each state in terms of presence and extent (e.g., frequency, % of funding allocated), continuity (e.g., anticipated vs. unintended discontinuation), and barriers and facilitators to use (based on CFIR [96]). We will examine associations with outcomes based on exploratory statistical tests, heavily leveraging qualitative data to ensure accurate interpretation and maximize depth of understanding. At various points in the analysis, a given pair of states may be grouped together or contrasted, depending on the characteristic being considered. We will also consider differences in perspective among the participant groups.

Following those initial analyses, we will develop our sampling strategy for focus groups [56, 69, 109] by grouping together states that represent related cases (e.g., identified similar policy-related barriers and facilitators; used funds to develop similar EBP infrastructure elements). To identify implications, we expect to conduct three focus groups of state administrators and their partners plus a federal administrator group. We will use a rapid coding approach to conventional content analysis, developed for implementation research [110], to analyze focus group data. This analytic approach allows researchers to identify discrete, pre-determined information (e.g., barriers and facilitators to policy changes) that can guide practical decision-making [111]. The facilitators will take field notes during each focus group and synthesize the notes into themes once all groups are completed. Focus groups will also be audio-recorded and transcribed to allow for verification and identifying key quotations. We will integrate identified policy implications into the case study summaries, using other data as needed to help contextualize and understand the focus group input. Ultimately, we will update the policy brief reviewed by the focus groups to reflect key project findings, including implications from the focus groups and a summary of the state-level case studies, and disseminate the brief to help policymakers understand financing EBPs for adolescent SUD through federal grants.

## Discussion

Effective strategies are needed that can secure and direct financial resources to support EBP implementation in adolescent SUD treatment services. This project aims to compare two grant-making strategies used by the U.S. SAMHSA Center for Substance Abuse Treatment on the penetration (i.e., widespread adoption) and sustained use of A-CRA over the past 15+ years. This research will be challenging due to its reliance on a natural experiment, but our mixed-methods approach will provide an in-depth, comprehensive understanding of these financing strategies with practical implications for policymakers.

This research project will be the first to directly compare the effects of state-focused versus organization-focused grants on EBP penetration and sustainment. Understanding the outcomes, moderators, and mediators of different grant types has important implications for how SAMHSA and other entities administer future support for SUD EBPs such as A-CRA. Due to its novelty, our project also has implications for financing strategies across diverse EBPs in behavioral health, medicine, public health, and prevention; we will document contextual factors that funders should consider when generalizing our results to their own grant mechanisms. Furthermore, our novel measurement and analytic approaches (e.g., non-equivalent-dependent variables, a multi-element measure of sustainment, policy implementation frameworks) represent significant advances for implementation science that will be of broad interest to the field [51, 96].

Our research approach has several limitations. First, we ideally would have evaluated clinical outcomes of A-CRA, rather than assuming youth improvement based on fidelity scores. However, state agencies use highly variable methods to collect and track client-level data, so aggregating and analyzing such data was not feasible for the proposed research. Focusing on implementation outcomes that are related to clinical outcomes is the most feasible option. Second, we will only have enough statistical power to examine outcomes at the organizational level, yet the major advantage of state-focused grants may be their ability to support state-level penetration and sustainment outcomes. If our primary findings warrant it, we may use simple descriptive and inferential statistics to compare state-level outcomes in states that received organization-focused versus state-focused grants, acknowledging that these analyses will be exploratory only. Third, we may not find the hypothesized difference between organization-focused and state-focused strategies for a variety of reasons (e.g., more variation in outcomes within financing strategies than between strategies). Regardless, we can still identify policymakers’ perspectives on more impactful grant-making approaches—and their potential mechanisms—through aim 3.

## Conclusions

The proposed research will substantially advance knowledge in implementation science by examining, for the first time, the different outcomes of two federal financing strategies that directly target EBP penetration and sustainment. Without a better understanding of financing strategies, the public health impact of high-quality and implementation-ready treatments like A-CRA will likely remain low. This project represents an important step in increasing the availability of high-quality adolescent SUD treatment while simultaneously advancing implementation science as a whole.

## Supplementary Information

**Additional file 1.** StaRI checklist for A-CRA Financing.

**Additional file 2.** Interview protocols for A-CRA Financing.

**Additional file 3.** Survey items for A-CRA Financing.

**Additional file 4.** Analysis plan for A-CRA Financing.

## Data Availability

Data sharing is not applicable for the original data collection described in this article, as it is a study protocol and no datasets have been generated or analyzed. Other materials (e.g., data collection instruments) are available in the Additional files accompanying this article or from the corresponding author on reasonable request. See the publications from our previous project [16, 33, 34] for details regarding data-sharing of the previously collected datasets.

## Abbreviations

A-CRA: Adolescent Community Reinforcement Approach
CFIR: Consolidated Framework for Implementation Research
CHS: Chestnut Health Systems
EBP: Evidence-based practice
EPIS: Exploration Preparation Implementation Sustainment framework
N-SSATS: National Survey of Substance Abuse Treatment Services
SAMHSA: Substance Abuse and Mental Health Services Administration
StaRI: Standards for Reporting Implementation Studies
SUD: Substance use disorder
