# Supplementary material for: Comparing two federal financing strategies on penetration and sustainment of the adolescent community reinforcement approach for substance use disorders: protocol for a mixed-method study
Source: Implement Sci Commun. 2022 May 13;3:51. doi: 10.1186/s43058-022-00298-y (PMC9099033; doi:10.1186/s43058-022-00298-y)
Supplement: Supplementary file 2 — Additional file 2. Interview protocols for A-CRA Financing. [file 43058_2022_298_MOESM2_ESM.docx]

# A-CRA Financing Project: STATE ADMIN INTERVIEW COVER SHEET

**RESPONDENT ID(s):**

**RESPONDENT NAME(s):**

**STATE ID:**

**RESPONDANT STATE:**

**STATE AGENCY NAME:**

**DATE OF INTERVIEW: ______ / ______ /_______**

**TIME OF INTERVIEW (INTERVIEWEE TIME): _______ PT/MT/CT/ET**

**INTERVIEWER:**

**PHONE NUMBER FOR INTERVIEW:**

**E-MAIL:**

**A-CRA GRANTS RECEIVED:**

**SAT-ED START DATE: ______ / ______ /_______ END DATE: ______ / ______ /_______**

**SYT START DATE: ______ / ______ /_______ END DATE: ______ / ______ /_______**

**SYT-I START DATE: ______ / ______ /_______ END DATE: ______ / ______ /_______**

**STR START DATE: ______ / ______ /_______ END DATE: ______ / ______ /_______**

**YT-I START DATE: ______ / ______ /_______ END DATE: ______ / ______ /_______**

**other START DATE: ______ / ______ /_______ END DATE: ______ / ______ /_______**

**(if other, describe: ___________________ )**

**OTHER NAMES FOR THE SAMHSA/CSAT-FUNDED A-CRA IMPLEMENTATION PROJECT IN THIS STATE:**

**RELEVANT CASE NOTES:**

***STATE ADMIN INTERVIEW VERBAL CONSENT***

(read prior to beginning interview/recording)

Hi [STATE ADMIN NAME],

This is [INTERVIEWER NAME] calling from RAND to speak with you about [STATE AGENCY NAME]’s experiences with implementing the Adolescent Community Reinforcement Approach (A-CRA) treatment model in [STATE]. Is this still a good time to complete your interview? To ensure confidentiality, make sure you are in a private, secure location for completing the interview.

>IF NO, reschedule.

>IF YES, great.

We would like a representative from [STATE AGENCY NAME] to answer these questions. Ideally, it should be someone knowledgeable about your A-CRA implementation efforts. Are you the right person at your agency to participate in this opportunity?

>IF NO: May I get the contact information of the best person to speak with about the state’s youth/adolescent substance use treatment programs (especially A-CRA implementation)? [RECORD CONTACT INFORMATION TO FOLLOW-UP]

>IF YES, thanks for confirming.

Before we begin, let me assure you that your responses to these questions will be held in strict confidence. In collaboration with Chestnut Health Systems, we are requesting interviews with state agency representatives in nearly 20 states that received funding from the SAMHSA Centers for Substance Abuse Treatment (CSAT) to deliver A-CRA. We will use information from the interviews to understand how different CSAT funding models influence the implementation and sustainability of A-CRA. We will also examine information provided by treatment organizations that implemented A-CRA as part of CSAT grants like yours through surveys and interviews. We will not attribute comments to specific individuals or programs in any of our reports or publications. Your responses will not be shared with your agency or with SAMHSA.

Today’s interview will last about 60 minutes. You will receive a $25 Amazon e-gift card as a thank you for your participation.

Your participation in this discussion is entirely voluntary. We would like to have your responses to all of the questions. However, if you’re uncomfortable with any question we can skip it, and you can stop the interview at any time. There are no right or wrong answers – we are interested in your perspectives and experiences. After the interview, we may follow up with you about sharing documentation related to your CSAT grant that would be useful for us to review. It will always be your decision whether to share. Finally, we would like to audio-record the interview to ensure that we capture everything that is said. We will destroy the recording once we confirm we have captured everything in our de-identified notes and transcripts. However, you can still participate in the interview even if you do not give permission to audio-record.

If you have any questions or want to discuss the project further at any time, you may always contact us at 360-578-2911 or at pham@rand.org. Furthermore, if you have questions about your rights as a research participant or need to report a research-related injury or concern, you can contact RAND's Human Subjects Protection Committee toll-free at (866) 697-5620 or email [hspcinfo@​rand.org](mailto:hspcinfo@​rand.org)​. If you contact the Committee, please reference Study #2020-N0887.

- **Do you have any questions?**
- **Are you willing to take part in this discussion?**

>IF NO: That is not a problem, thank you for your time.

>IF YES, great.

- **Is it ok with you if we audio-tape this discussion?**

>IF NO: That is not a problem. I can take notes while we’re talking so I don’t miss anything important, though that means we might proceed through the interview more slowly than usual. I could also arrange for a colleague to take notes during the interview. [RESCHEDULE IF NEEDED]

>IF YES, perfect, let’s get started.

**Interview Protocol – STATE BEHAVIORAL HEALTH AGENCY ADMINISTRATOR**

1. Tell me a little bit about the state agency where you work. What types of services does your agency oversee or administer? (e.g., age groups, problem types, etc.)

2. What is your primary professional role(s) at your agency? (What are you responsible for overseeing?)

3. At the state agency, have you worked on any SAMHSA/CSAT-funded projects to disseminate A-CRA? Yes or No?

🞏 No **– SKIP TO QUESTION 4**🞏 Yes - **CONTINUE**

3A. [IF YES] Between what dates did you work on the project(s)? Your best guess is fine.

Enter Start Date (MM/YY)

Enter End Date (MM/YY)

4. What do you think of A-CRA as a treatment for youth – does it meet the needs of the populations you serve? How so or why not? Of note, throughout this interview, by “youth” we typically mean ages 12 through 17 (or through age 24 if A-CRA training in your state included young adults).

5. What do think of A-CRA as a treatment for outpatient youth substance use treatment services – does it fit well into treatment organizations’ services and capabilities? How so or why not?

6. How has your state agency selected treatment organizations that you worked with to support A-CRA implementation?

*[IF NEEDED, PROMPT: I am asking how you decided which treatment organizations got to participate in A-CRA implementation activities, like sending their clinicians to A-CRA training. This includes both initial “lab sites” or “dissemination sites,” as well as other organizations worked with during or after the CSAT grant.]*

7. Next, I’m going to list seven activities that your state agency might have engaged in its efforts to help provider organizations implement A-CRA. Please let me know whether your state agency engaged in each of these activities, and if so, any details you know about what was done and when.

7A. Providing training for organizations to implement A-CRA?

🞏 No
🞏 Yes - during the SAMHSA CSAT grant period

🞏 Yes - currently engaging in this activity

🞏 Yes - planning to continue over the coming year

🞏 Don't Know

IF YES, please describe [PROBE AS NEEDED: What have those activities looked like in your state? Who and what have been involved? How has the activity changed since the grant funding period, if at all?]:

7B. Directly funding organizations to implement A-CRA?

🞏 No
🞏 Yes - during the SAMHSA CSAT grant period

🞏 Yes - currently engaging in this activity

🞏 Yes - planning to continue over the coming year
🞏 Don't Know

IF YES, please describe [PROBE AS NEEDED: What have those activities looked like in your state? Who and what have been involved? How has the activity changed since the grant funding period, if at all?]:

7C. Developing state policies that promote use of A-CRA?

*[INTWR, IF NEEDED: examples of state policies might include:*

*-the state requires that providers use an evidence based treatment (EBT) and A-CRA is an EBT*

*-enhanced billing rates for using A-CRA]*

🞏 No
🞏 Yes - during the SAMHSA CSAT grant period

🞏 Yes - currently engaging in this activity

🞏 Yes - planning to continue over the coming year
🞏 Don't Know

IF YES, please describe [PROBE AS NEEDED: What have those activities looked like in your state? Who and what have been involved? How has the activity changed since the grant funding period, if at all?]:

7D. Supporting organizations’ sustainability planning for use of A-CRA?

*[INTWR, IF NEEDED: examples of sustainability planning might include:*

*-providing templates or worksheets for developing an A-CRA sustainability plan*

*-offering feedback or guidance on A-CRA sustainability plans]*

🞏 No
🞏 Yes - during the SAMHSA CSAT grant period

🞏 Yes - currently engaging in this activity

🞏 Yes - planning to continue over the coming year
🞏 Don't Know

IF YES, please describe [PROBE AS NEEDED: What have those activities looked like in your state? Who and what have been involved? How has the activity changed since the grant funding period, if at all?]:

7E. Supporting organizations’ efforts to promote youth/family engagement?

*[INTWR, IF NEEDED: examples of engagement support might include:*

*-providing example protocols or procedures for outreach activities meant to engage youth/families in A-CRA*

*-offering feedback or guidance on youth/family engagement plans: position descriptions, procedures, etc.]*

🞏 No
🞏 Yes - during the SAMHSA CSAT grant period

🞏 Yes - currently engaging in this activity

🞏 Yes - planning to continue over the coming year
🞏 Don't Know

IF YES, please describe [PROBE AS NEEDED: What have those activities looked like in your state? Who and what have been involved? How has the activity changed since the grant funding period, if at all?]:

7F. Promoting organizations’ capacity for inter-organizational coordination?

*[INTWR, IF NEEDED: examples of supporting inter-organizational coordination might include:*

*-working with other state agencies, like child welfare or juvenile justice, to build their capacity to support A-CRA treatment organizations*

*-helping treatment organizations build capacity to coordinate with outside treatment providers as part of A-CRA treatment delivery, or as part of learning A-CRA such as learning collaboratives]*

🞏 No
🞏 Yes - during the SAMHSA CSAT grant period

🞏 Yes - currently engaging in this activity

🞏 Yes - planning to continue over the coming year
🞏 Don't Know

IF YES, please describe [PROBE AS NEEDED: What have those activities looked like in your state? Who and what have been involved? How has the activity changed since the grant funding period, if at all?]:

7G. Promoting organizations’ improving their computer systems or electronic health records?

🞏 No
🞏 Yes - during the SAMHSA CSAT grant period

🞏 Yes - currently engaging in this activity

🞏 Yes - planning to continue over the coming year
🞏 Don't Know

IF YES, please describe [PROBE AS NEEDED: What have those activities looked like in your state? Who and what have been involved? How has the activity changed since the grant funding period, if at all?]:

7H. Are there other activities or resources your state agency has provided to support A-CRA implementation?

🞏 No
🞏 Yes - during the SAMHSA CSAT grant period

🞏 Yes - currently engaging in this activity

🞏 Yes - planning to continue over the coming year
🞏 Don't Know

IF YES, please describe [PROBE AS NEEDED: What have those activities looked like in your state? Who and what have been involved? How has the activity changed since the grant funding period, if at all?]:

8. What implementation activities have you personally been involved in as part of state efforts to implement A-CRA?

8A. Providing training for organizations to implement A-CRA?

🞏 No
🞏 Yes

8B. Directly funding organizations to implement A-CRA?

🞏 No
🞏 Yes

8C. Developing state policies that promote use of A-CRA?

🞏 No
🞏 Yes

8D. Supporting organizations’ sustainability planning for use of A-CRA?

🞏 No
🞏 Yes

8E. Supporting organizations’ efforts to promote youth/family engagement?

🞏 No
🞏 Yes

8F. Promoting organizations’ capacity for inter-organizational coordination?

🞏 No
🞏 Yes

8G. Promoting organizations’ improving their computer systems or electronic health records?

🞏 No
🞏 Yes

8H. Other activities or resources your state agency currently provides to support A-CRA implementation?
🞏 No
🞏 Yes

9. How has your state agency obtained funding for continuing the A-CRA implementation activities or resources that you started during the SAMHSA CSAT grant? These are the activities and resources discussed in the previous series of questions – beyond paying for services. I’m going to read five options, please let me know which ones your agency has used.

9A. Internal funding from agency's budget?

🞏 No
🞏 Yes
🞏 Don't Know

IF YES, please describe:

9B. State budget appropriations?

🞏 No
🞏 Yes
🞏 Don't Know

IF YES, please describe:

9C. Block grant funds?
🞏 No
🞏 Yes
🞏 Don't Know

IF YES, please describe:

9D. Federal grants (like SAMHSA CSAT)?

🞏 No
🞏 Yes
🞏 Don't Know

IF YES, please describe:

9E. Payments from a special funding source (for example, a dedicated tax or bond, settlement fund, etc.)?

🞏 No
🞏 Yes
🞏 Don't Know

IF YES, please describe:

9F. Other sources of funding?

🞏 No
🞏 Yes
🞏 Don't Know

IF YES, please describe:

10. Currently, what funding options does your state agency offer provider organizations to support youth substance use treatment delivery in general?

*[IF NEEDED: By sources of funding, we mean any source from which the provider organization receives money to pay for costs related to treatment delivery].*

*[IF NEEDED: Examples could include awarding grants or contracts to provider organizations, offering pass-through funds from federal grants or other sources, administering Medicaid/CHIP public health insurance, and value-based or outcomes-based payment systems.]*

11. What funding options, if any, does your agency currently offer to support provider organizations that deliver A-CRA specifically? By this, I mean funding for A-CRA activities or services begun during the SAMHSA CSAT grant.

*[IF NEEDED: By sources of funding, we mean any source from which the provider organization receives money to pay for costs related to A-CRA delivery].*

*[IF NEEDED: examples could include awarding grants or contracts to provider organizations that specify use of A-CRA, offering pass-through funds from federal grants or other sources to support A-CRA activities, increased billing rates for A-CRA, value-based or outcomes-based payment systems tied to A-CRA outcomes, etc.]*

12. The next few questions ask about how successful your state effort has been at implementing A-CRA. Please answer to the best of your knowledge, in the organizations/settings you would know about. I will ask you about success in three areas.

12A. How successful has your state effort been in training clinicians in A-CRA at provider organizations in your state?

*[IF NEEDED: You might think of factors like the number of clinicians trained, diversity of regions or organizations represented in trainings, etc.]*

12B. How successful has your state effort been in getting clinicians certified in A-CRA?

*[IF NEEDED: By certified, we mean they completed all training requirements – such as coaching and submission of recorded sessions – and received written documentation of certification. This includes first-level, full, transitional age youth, and supervisor certifications.]*

12C. How successful has your state effort been in youth receiving A-CRA in your state?

*[IF NEEDED: You might think of factors like the number of youth completing treatment, ability to treat the highest-need youth populations, etc.]*

13. Tell me about any ways in which your state agency has collaborated with other entities to support A-CRA.

*[IF NEEDED, PROMPT: For example, you may have engaged in training or support activities with your state substance use services authority and/or Chestnut Health Systems, or have partnered with universities or research institutions on research related to A-CRA].*

13A. Which of these collaborations is your agency continuing, if any? Why or why not?

14. Tell me about any efforts or planning at your state agency to obtain resources for continuing A-CRA beyond your state’s grant period. By resources, we mean things needed to support A-CRA such as money, staff, supervision, training, and A-CRA manuals.

*[IF NEEDED, PROMPT: By strategic planning, we mean the process by which an organization defines its strategy or direction and makes decisions about how to allocate resources to pursue the strategy.]*

14A. Which of these efforts or planning is your agency continuing, if any? Why or why not?

15. Can you please describe any state or federal policies that have supported A-CRA delivery? These could include funding and billing rules that support A-CRA delivery. Include policies at your state agency and other agencies.

*[IF NEEDED, PROMPT: Here are some examples:*

*-State/federal agency/insurance requires use of an evidence based treatment (EBT) and A-CRA is an EBT*

*-Our agency’s mission emphasizes involvement of the family, A-CRA does that]*

15A. Which of these policies are currently supporting A-CRA delivery, if any?

16. What about state or federal policies that interfere with A-CRA delivery? (Including at your agency and other agencies)

*[IF NEEDED, PROMPT: Here are some examples:*

*-if a state decided to require that substance use clinics deliver a different treatment (other than A-CRA), this policy might interfere with the delivery of A-CRA.*

*- if certain insurance plans stopped reimbursing for A-CRA or the full number of sessions, this policy could interfere with A-CRA delivery.]*

16A. Which of these policies are currently impacting A-CRA delivery, if any?

17. Can you tell me about any pressure your state agency has experienced to continue delivering A-CRA or to discontinue its use?

*[IF NEEDED, PROMPT: Pressure to deliver A-CRA could come from a variety of sources. For ex., organizations might experience pressure if there is some financial incentive to deliver A-CRA. If a state department of health decides its mission is to deliver evidence-based treatment for substance use, this could also be seen as pressure.]*

18. Would you say provider organizations in your state are generally supportive or reluctant to use A-CRA? Can you give me an example?

19. Next, I would like to know what factors helped efforts to implement and sustain A-CRA in your state. Please let me know if these factors were important in your state, and if yes, how so.

*[PROBE AS NEEDED: What factors were most helpful in that domain? Can you give me some examples? Were there important changes in which factors were most helpful once funding ended?]*

19A. Would you say characteristics of A-CRA helped efforts to implement and sustain A-CRA in your organization?  Yes or No?

*[IF NEEDED: for example, treatment content, structure of sessions, etc.]*

🞏 No
🞏 Yes

IF YES, please describe: When was that factor most impactful? Was it during the CSAT grant funding period, after the CSAT funding ended, or throughout?

19B. Would you say characteristics of key individuals helped efforts to implement and sustain A-CRA in your organization?  Yes or No?

*[IF NEEDED: “key individuals” are people in the state who had a major positive impact on A-CRA implementation; could be within or outside of your state agency]*

🞏 No
🞏 Yes

IF YES, please describe: When was that factor most impactful? Was it during the CSAT grant funding period, after the CSAT funding ended, or throughout?

19C. Would you say characteristics of treatment organizations helped efforts to implement and sustain A-CRA in your organization?  Yes or No?

🞏 No
🞏 Yes

IF YES, please describe: When was that factor most impactful? Was it during the CSAT grant funding period, after the CSAT funding ended, or throughout?

19D. Would you say helpful factors within your state agency helped efforts to implement and sustain A-CRA in your organization?  Yes or No?

*[IF NEEDED: for example, organizational leadership, staffing patterns, scheduling appointments, etc.]*

🞏 No
🞏 Yes

IF YES, please describe: When was that factor most impactful? Was it during the CSAT grant funding period, after the CSAT funding ended, or throughout?

19E. Would you say helpful factors outside your state agency helped efforts to implement and sustain A-CRA in your organization?  Yes or No?

*[IF NEEDED: for example, state/federal leadership, climate/culture re: substance use]*

🞏 No
🞏 Yes

IF YES, please describe: When was that factor most impactful? Was it during the CSAT grant funding period, after the CSAT funding ended, or throughout?

19F. Would you say partnerships with other agencies or organizations helped efforts to implement and sustain A-CRA in your organization?  Yes or No?

🞏 No
🞏 Yes

IF YES, please describe: When was that factor most impactful? Was it during the CSAT grant funding period, after the CSAT funding ended, or throughout?

19G. Were there other helpful factors for implementing and sustaining A-CRA?

🞏 No
🞏 Yes

IF YES, please describe: When was that factor most impactful? Was it during the CSAT grant funding period, after the CSAT funding ended, or throughout?

20. Now, I’m going ask how the same six categories of factors may have included barriers that hindered efforts to implement and sustain A-CRA in your state. Please let me know if these factors were important in your state, and if yes, how so.

*[PROBE AS NEEDED: What factors were the biggest barriers in that domain? Can you give me some examples? Were there important changes in which factors were the biggest barriers once funding ended?]*

20A. Would you say characteristics of A-CRA hindered efforts to implement and sustain A-CRA in your organization? Yes or No?

*[IF NEEDED: for example, treatment content, structure of sessions, etc.]*

🞏 No
🞏 Yes

IF YES, please describe: When was that factor most impactful? Was it during the CSAT grant funding period, after the CSAT funding ended, or throughout?

20B. Would you say challenges with key individuals hindered efforts to implement and sustain A-CRA in your organization? Yes or No?

*[IF NEEDED: “key individuals” are people in the state who presented major challenges for A-CRA implementation; could be within or outside of your state agency]*

🞏 No
🞏 Yes

IF YES, please describe: When was that factor most impactful? Was it during the CSAT grant funding period, after the CSAT funding ended, or throughout?

20C. Would you say challenges with treatment organizations hindered efforts to implement and sustain A-CRA in your organization? Yes or No?

🞏 No
🞏 Yes

IF YES, please describe: When was that factor most impactful? Was it during the CSAT grant funding period, after the CSAT funding ended, or throughout?

20D. Would you say challenges within your state agency hindered efforts to implement and sustain A-CRA in your organization? Yes or No?

*[IF NEEDED: for example, organizational leadership, staffing patterns, scheduling appointments, etc.]*

🞏 No
🞏 Yes

IF YES, please describe: When was that factor most impactful? Was it during the CSAT grant funding period, after the CSAT funding ended, or throughout?

20E. Would you say challenges outside your state agency hindered efforts to implement and sustain A-CRA in your organization? Yes or No?

*[IF NEEDED: for example, state/federal leadership, climate/culture re: substance use]*

🞏 No
🞏 Yes

IF YES, please describe: When was that factor most impactful? Was it during the CSAT grant funding period, after the CSAT funding ended, or throughout?

20F. Would you say issues in partnerships with other agencies or organizations hindered efforts to implement and sustain A-CRA in your organization? Yes or No?

🞏 No
🞏 Yes

IF YES, please describe: When was that factor most impactful? Was it during the CSAT grant funding period, after the CSAT funding ended, or throughout?

20G. Were there other barriers to implementing and sustaining A-CRA?

🞏 No
🞏 Yes

IF YES, please describe: When was that factor most impactful? Was it during the CSAT grant funding period, after the CSAT funding ended, or throughout?

Next, I’m going to ask you a few questions about the COVID-19 pandemic.

21. How has the COVID-19 pandemic affected substance use treatment services in your state?

22. When the pandemic began in March 2020, were any treatment organizations delivering A-CRA in your state?

🞏 No – ***IF NO, SKIP TO QUESTION 25***
🞏 Yes - ***CONTINUE***

🞏 Don’t know – ***SKIP TO QUESTION 25***

**IF QUESTION 22 = YES**

23. What have been the most significant changes affecting A-CRA programs in your state due to COVID-19?

*[PROBE AS NEEDED:*

*…Organizations discontinued A-CRA due to COVID-related changes*

*…Changes in referrals to A-CRA*

*…Changes in how A-CRA assessment, treatment, or case management services are delivered*

*…Changes in staffing*

*…Remote work*

*…Telehealth service delivery*

*Were there other changes? If so, please describe: ]*

24. Have there been changes made in response to COVID-19 that have been beneficial, and will those changes continue beyond the pandemic?

*[PROBE AS NEEDED:*

*…Changes in referrals to A-CRA*

*…Changes in how A-CRA assessment, treatment, or case management services are delivered*

*…Changes in staffing*

*…Remote work*

*…Telehealth service delivery*

*Were there other beneficial changes? If so, please describe: ]*

OK, we are almost done. Next, I want to ask you about your general impressions regarding the SAMHSA CSAT-funded A-CRA project and then have a few questions about you.

25. If you had a chance to participate in a SAMHSA CSAT project again, would you consider it?

🞏 No
🞏 Yes

26. Why/why not?

27. Is there anything you would change about the SAMHSA CSAT project in order to improve the sustainment of A-CRA in your state?

28. How many years of experience do you have in the administration of substance use treatment services?

29. How many years of experience do you have in your current position and similar positions/levels? This would include experience at other agencies, if relevant.

30. Do you have any experience as a provider of substance use treatment services?

🞏 No – ***IF NO, SKIP TO question 32***

🞏 Yes - ***CONTINUE***

**IF QUESTION 30 = YES**

31. How many years of experience do you have as a provider?

32. Is there anything else you would like to share related to the topic of sustaining A-CRA in your state?

33. Are there written reports or other documentation that you could share with us that might give us insight into your state’s A-CRA initiative? This could include anything that helps us understand the supports provided by your state agency and/or the services provided by treatment organizations that implemented A-CRA. Documents from the grant period are useful, as are documents from the time period since the grant ended. Even redacted or generic/incomplete documents could be very useful to us. I will send you an email after this call with a list, and you can let us know if they’d be available to share and any relevant considerations.

[IF TIME] Here is a list of documents that state agencies might be able to share. Would you be able to share any of the following?  If so, can you describe the documents and any next steps for sharing them?

- CSAT grant progress reports and/or applications?
- Data or information related to CSAT grant progress, such as evaluation reports?
- Contracts with A-CRA treatment organizations, from during the CSAT grant?
- Contracts with A-CRA treatment organizations, from after the CSAT grant?
- Planning tools, e.g., “financial maps” of A-CRA funding, from during the CSAT grant? (Financial maps were required as a sustainability planning tool for some grantees)
- Planning tools, e.g., “financial maps” of A-CRA funding, from after the CSAT grant?
- Information disseminated about your state A-CRA initiative for a broader audience? For example: presentations, articles, media coverage, sharing on social media, etc.
- COVID-19 response plans relevant for A-CRA?
- Other written reports or documentation that might give us insight into your state’s A-CRA initiative?

[TURN OFF RECORDER]

That's all the interview questions I have. Thank you for providing this information. We will be sending your $25 Amazon e-gift card by e-mail within the next few days. [IF STATE ADMINISTRATOR INTERVIEW OR GROUP INTERVIEW, FIGURE OUT WHO THE GIFT CARD SHOULD GO TO (ONLY ONE PER STATE). IT IS POSSIBLE THEY CANNOT ACCEPT THE GIFT CARD.]

[IF PARTICIPANT WILL BE INTERVIEWED AGAIN, complete the tracking module beginning on the final page]

# A-CRA Financing Project: CLINICIAN AND/OR SUPERVISOR INTERVIEW COVER SHEET

**RESPONDENT ID:**

**RESPONDENT NAME:**

**TREATMENT SITE ID:**

**TREATMENT SITE NAME:**

**STATE ID:**

**STATE:**

**PHONE#:**

**EMAIL:**

**DATE OF INTERVIEW: ______ / ______ /_______**

**TIME OF INTERVIEW (INTERVIEWEE TIME): _______ PT/MT/CT/ET**

**INTERVIEWER: ______________________**

**A-CRA Status Expected: Sustainer Non-Sustainer Unknown**

**Title/Position Expected: Clinician Supervisor Clinician & Supervisor Other Unknown**

**CHS RECORD OF TRAINING? Yes No Unknown**

**RESPONDANT STATE: ______________________**

**A-CRA GRANTS RECEIVED:**

**SAT-ED START DATE: ______ / ______ /_______ END DATE: ______ / ______ /_______**

**SYT START DATE: ______ / ______ /_______ END DATE: ______ / ______ /_______**

**SYT-I START DATE: ______ / ______ /_______ END DATE: ______ / ______ /_______**

**STR START DATE: ______ / ______ /_______ END DATE: ______ / ______ /_______**

**YT-I START DATE: ______ / ______ /_______ END DATE: ______ / ______ /_______**

**other START DATE: ______ / ______ /_______ END DATE: ______ / ______ /_______**

**(if other, describe: ___________________ )**

**PLAN TO INTERVIEW IN WAVE 2? Yes No**

**OTHER NAMES FOR THE SAMHSA/CSAT-FUNDED A-CRA IMPLEMENTATION PROJECT IN THIS STATE:**

**RELEVANT CASE NOTES:**

***CLINICIAN INTERVIEW VERBAL CONSENT***

(READ PRIOR TO BEGINNING INTERVIEW/RECORDING)

Hi [CLINICIAN NAME],

This is [INTERVIEWER NAME] calling from RAND to speak with you about the youth substance use treatment program where you work and any experiences you have with implementing the Adolescent Community Reinforcement Approach (A-CRA) treatment model. Is this still a good time to complete your interview? To ensure confidentiality, make sure you are in a private, secure location for completing the interview.

>IF NO, RESCHEDULE.

>IF YES, great.

We would like a clinician and/or clinical supervisor for youth substance use treatment to answer these questions. Ideally, it should be someone knowledgeable about your A-CRA program. Are you the right person at your organization to participate in this opportunity?

>IF NO: May I please have the contact information of the best contact(s) for youth substance use treatment? [RECORD CONTACT INFORMATION TO FOLLOW-UP]

>IF YES, thanks for confirming.

Before we begin, let me assure you that your responses to these questions will be held in strict confidence. In collaboration with Chestnut Health Systems, we are requesting interviews and surveys from clinicians and supervisors from nearly 20 states that received A-CRA training from Chestnut Health, as part of SAMHSA Center of Substance Abuse Treatment (CSAT) grants received by state substance use services authorities. We will use information from the interviews and surveys to understand how different CSAT funding models influence the sustainability of A-CRA delivery after funding ends. We will also examine information provided by state organizations that received the CSAT grants and data already collected from prior CSAT grantee organizations that implemented A-CRA. We will not attribute comments to specific individuals or programs in any of our reports or publications. Your responses will not be shared with your organization or with SAMHSA.

Today’s interview will last up to 45 minutes. Afterwards, we will ask you to complete a 30-minute online survey on your own to give us a more complete picture. You will receive a $50 Amazon electronic gift card upon completion of the web survey as a thank you for your participation.

Your participation in this discussion and the survey is entirely voluntary. We would like to have your responses to all of the questions. However, if you’re uncomfortable with any question we can skip it, and you can stop the interview at any time. There are no right or wrong answers – we are interested in your perspectives and experiences. Finally, we would like to audio-record the interview to ensure that we capture everything that is said. We will destroy the recording once we confirm we have captured everything in our de-identified notes and transcripts. However, you can still participate in the interview even if you do not give permission to audio-record.

If you have any questions or want to discuss the project further at any time, you may always contact us at 360-578-2911 or at pham@rand.org. Furthermore, if you have questions about your rights as a research participant or need to report a research-related injury or concern, you can contact RAND's Human Subjects Protection Committee toll-free at (866) 697-5620 or email [hspcinfo@​rand.org](mailto:hspcinfo@​rand.org)​. If you contact the Committee, please reference Study #2020-N0887.

- **Do you have any questions?**
- **Are you willing to take part in this discussion?**

>IF NO: That is not a problem, thank you for your time.

>IF YES, great.

- **Is it ok with you if we audio-tape this discussion?**

>IF NO: That is not a problem. I can take notes while we’re talking so I don’t miss anything important, though that means we might proceed through the interview more slowly than usual. I could also arrange for a colleague to take notes during the interview. [RESCHEDULE IF NEEDED]

>IF YES, perfect, let’s get started.

- **[IF UNKNOWN] Based on the background information we’ve received, I am not sure whether your organization currently delivers A-CRA. Can you confirm whether or not A-CRA is delivered by clinicians at your organization?**

>IF NO: BEGIN NON-SUSTAINER INTERVIEW

>IF YES: BEGIN SUSTAINER INTERVIEW

**Sustainer Interview – CLINICIAN**

1. Based on the background information we’ve received, it sounds like your agency currently delivers the Adolescent Community Reinforcement Approach or A-CRA. Is this correct?

🞏 No – ***SWITCH TO NON-SUSTAINER INTERVIEW***🞏 Yes - ***CONTINUE***

2. Tell me a little bit about the substance use services at your agency. What services are available? What types of clients do you serve? (e.g., age range, services for specific substances).

3. What youth age range do you use A-CRA with at your agency? Throughout this interview, by “youth” we typically mean ages 12 through 17 (or through age 24 if your A-CRA training included young adults).

4. What is your primary professional role(s) at your clinic/site?

🞏 Clinical Supervisor – ***SWITCH TO SUPERVISOR ONLY VERSION***

🞏 Counselor or Clinician – ***CONTINUE***
🞏 Clinical Supervisor and Counselor/Clinician ***– SWITCH TO SUPERVISOR + CLINICIAN VERSION***

🞏 Other, please describe: ____________________(e.g., administrator, grant manager) ***- SWITCH TO SUPERVISOR VERSION***

5. Did you participate in any SAMHSA/CSAT-funded A-CRA training initiatives in your state? Yes or No?

*[IF NEEDED, PROMPT AS RELEVANT: Our records do/do not indicate that you received training in A-CRA as part of a SAMHSA CSAT grant under the [initiative] in [grants years]. (IF KNOWN) In your state, I believe this initiative was called [NAME]. So would it be accurate to say you did/did not participate in your state’s A-CRA training initiative?]*

🞏 No – ***SKIP TO QUESTION 6***🞏 Yes – ***CONTINUE***

**IF QUESTION 5 = Yes**

5A. Between what dates did you work on the project? Your best guess is fine.

Enter Start Date (MM/YY)

Enter End Date (MM/YY)

6. What role(s) have you served in delivering A-CRA? ***[MARK ALL THAT APPLY]***

🞏 A-CRA Therapist
🞏 A-CRA Clinical Supervisor
🞏 Other (Please describe: ____________________________________________________)
🞏 No Role in delivering ACRA

7. Have you received any A-CRA certifications? This means that you completed all training requirements (such as coaching and submission of recorded sessions) and received written documentation of the certification.

***[MARK ALL THAT APPLY]***

*[IF NEEDED, PROMPT:  A-CRA supervisor certification is different from A-CRA certification.  It refers to being certified to provide A-CRA clinical supervision].*

🞏 First-level clinician certification (passed 9 A-CRA procedures)
🞏 Full clinician certification (passed all 19 A-CRA procedures)
🞏 Supervisor certification
🞏 A-CRA-TAY (Transitional Age Youth) certification

🞏 No, never certified in A-CRA

8. What do you think of A-CRA as a treatment for youth? Please note that throughout this interview, by “youth” we mean the population served during your SAMHSA CSAT grant periods.

9. Does your program currently offer any different types of treatment for youth with substance use disorders besides A-CRA?

🞏 No – ***SKIP TO QUESTION 11***
🞏 Yes - ***CONTINUE***
🞏 Don’t Know – ***SKIP TO QUESTION 11***

**IF QUESTION 9= YES**

10. What are those treatments called?

*[IF NEEDED, PROMPT: Examples of other treatments include Motivational Enhancement Therapy (MET), Cognitive Behavioral Therapy (CBT), Multidimensional Family Therapy (MDFT), 12-step facilitation, and supportive counseling as well as medication treatments like Suboxone or Naltrexone.*

*OR you can just describe the treatment. Is it outpatient? How long does it last? # of sessions/days/months?]*

11. Tell me about any ways in which your organization collaborates with other organizations to sustain A-CRA.

*[IF NEEDED, PROMPT: For example, you may have engaged in training or support activities with your state substance use services authority and/or Chestnut Health Systems, or have partnered with universities or research institutions on research related to A-CRA].*

12. Tell me about any planning that has been done to ensure resources are available to continue A-CRA. By resources, we mean things needed to support A-CRA. For example, this could include things like money, staff, supervision, training, and A-CRA manuals.

[*IF NEEDED, PROMPT: By strategic planning, we mean the process by which an organization defines its strategy or direction and makes decisions about how to allocate resources to pursue the strategy.]*

13. Can you please describe any policies that support A-CRA delivery? These could include external policies, such as funding source and billing rules that support A-CRA, as well as internal policies at your organization.

*[IF NEEDED, PROMPT: By policies, we mean either organizational, state, national, or local policies. Here are some examples:*

*-State/county/health insurance co requires that we use an evidence based treatment (EBT) and A-CRA is an EBT*

*-Our agency’s mission emphasizes involvement of the family, A-CRA does that]*

14. What about policies (external or internal) that interfere with A-CRA delivery?

*[IF NEEDED, PROMPT:  By policies, we mean either organizational, state, national, or local policies. Here are some examples:*

*-if a state decided to require that substance use clinics deliver a different treatment (other than A-CRA), this policy might interfere with the delivery of A-CRA.*

*- if certain insurance plans stopped reimbursing for A-CRA or the full number of sessions, this policy would interfere with A-CRA delivery.]*

15. Does A-CRA meet the needs of the populations you serve? How so or why not?

16. Can you tell me about any pressure your organization experienced to continue delivering A-CRA or to discontinue its use?

*[IF NEEDED, PROMPT: Pressure to deliver A-CRA could come from a variety of sources. For ex., organizations might experience pressure if there is some financial incentive to deliver A-CRA. If a state department of health decides its mission is to deliver evidence based treatment for substance use, this could also be seen as pressure.]*

17. Would you say staff are supportive or reluctant to use A-CRA? Can you give me an example of what people have said or done to indicate their supportiveness or reluctance toward A-CRA?

18. Next, I would like to know what factors helped efforts to implement and sustain A-CRA in your organization. Please let me know if these factors were important in your organization, and if yes, how so.

*[PROBE AS NEEDED: What factors were most helpful in that domain? Can you give me some examples? Were there important changes in which factors were most helpful once funding ended?]*

18A. Would you say characteristics of A-CRA helped efforts to implement and sustain A-CRA in your organization?  Yes or No?

*[IF NEEDED: for example, treatment content, structure of sessions, etc.]*

🞏 No
🞏 Yes

IF YES, please describe: When was that factor most impactful? Was it during the CSAT grant funding period, after the CSAT funding ended, or throughout?

18B. Would you say characteristics of key individuals helped efforts to implement and sustain A-CRA in your organization?  Yes or No?

*[IF NEEDED: “key individuals” are people who had a major positive impact on A-CRA implementation; could be within or outside of your treatment organization]*

🞏 No
🞏 Yes

IF YES, please describe: When was that factor most impactful? Was it during the CSAT grant funding period, after the CSAT funding ended, or throughout?

18C. Would you say client perspectives on A-CRA helped efforts to implement and sustain A-CRA in your organization?  Yes or No?

*[IF NEEDED: for example, if clients found A-CRA acceptable, appropriate, feasible, etc.]*

🞏 No
🞏 Yes

IF YES, please describe: When was that factor most impactful? Was it during the CSAT grant funding period, after the CSAT funding ended, or throughout?

18D. Would you say helpful factors within your organization helped efforts to implement and sustain A-CRA in your organization?  Yes or No?

*[IF NEEDED: for example, organizational leadership, staffing patterns, scheduling appointments, etc.]*

🞏 No
🞏 Yes

IF YES, please describe: When was that factor most impactful? Was it during the CSAT grant funding period, after the CSAT funding ended, or throughout?

18E. Would you say helpful factors outside your organization helped efforts to implement and sustain A-CRA in your organization?  Yes or No?

*[IF NEEDED: for example, state leadership, federal support, community factors]*

🞏 No
🞏 Yes

IF YES, please describe: When was that factor most impactful? Was it during the CSAT grant funding period, after the CSAT funding ended, or throughout?

18F. Would you say partnerships with other organizations helped efforts to implement and sustain A-CRA in your organization?  Yes or No?

🞏 No
🞏 Yes

IF YES, please describe: When was that factor most impactful? Was it during the CSAT grant funding period, after the CSAT funding ended, or throughout?

18G. Would you say funding helped efforts to implement and sustain A-CRA in your organization?  Yes or No?

🞏 No
🞏 Yes

IF YES, please describe: When was that factor most impactful? Was it during the CSAT grant funding period, after the CSAT funding ended, or throughout?

18H. Were there other helpful factors during or after the grant period?

🞏 No
🞏 Yes

IF YES, please describe: When was that factor most impactful? Was it during the CSAT grant funding period, after the CSAT funding ended, or throughout?

19. Now, I’m going ask how the same six categories of factors may have included barriers that hindered efforts to implement and sustain A-CRA in your organization. Please let me know if these factors were important in your organization, and if yes, how so.

*[PROBE AS NEEDED: What factors were the biggest barriers in that domain? Can you give me some examples? Were there important changes in which factors were the biggest barriers once funding ended?]*

19A. Would you say characteristics of A-CRA hindered efforts to implement and sustain A-CRA in your organization? Yes or No?

*[IF NEEDED: for example, treatment content, structure of sessions, etc.]*

🞏 No
🞏 Yes

IF YES, please describe: When was that factor most impactful? Was it during the CSAT grant funding period, after the CSAT funding ended, or throughout?

19B. Would you say challenges with key individuals hindered efforts to implement and sustain A-CRA in your organization? Yes or No?

*[IF NEEDED: “key individuals” are people who presented major challenges for A-CRA implementation; could be within or outside of your treatment organization]*

🞏 No
🞏 Yes

IF YES, please describe: When was that factor most impactful? Was it during the CSAT grant funding period, after the CSAT funding ended, or throughout?

19C. Would you say client perspectives on A-CRA hindered efforts to implement and sustain A-CRA in your organization? Yes or No?

*[IF NEEDED: for example, if clients found A-CRA acceptable, appropriate, feasible, etc.]*

🞏 No
🞏 Yes

IF YES, please describe: When was that factor most impactful? Was it during the CSAT grant funding period, after the CSAT funding ended, or throughout?

19D. Would you say challenges within your organization hindered efforts to implement and sustain A-CRA in your organization? Yes or No?

*[IF NEEDED: for example, organizational leadership, staffing patterns, scheduling appointments, etc.]*

🞏 No
🞏 Yes

IF YES, please describe: When was that factor most impactful? Was it during the CSAT grant funding period, after the CSAT funding ended, or throughout?

19E. Would you say challenges outside your organization hindered efforts to implement and sustain A-CRA in your organization? Yes or No?

*[IF NEEDED: for example, state leadership, federal support, community factors]*

🞏 No
🞏 Yes

IF YES, please describe: When was that factor most impactful? Was it during the CSAT grant funding period, after the CSAT funding ended, or throughout?

19F. Would you say issues in partnerships with other organizations hindered efforts to implement and sustain A-CRA in your organization? Yes or No?

🞏 No
🞏 Yes

IF YES, please describe: When was that factor most impactful? Was it during the CSAT grant funding period, after the CSAT funding ended, or throughout?

19G. Would you say funding hindered efforts to implement and sustain A-CRA in your organization? Yes or No?

🞏 No
🞏 Yes

IF YES, please describe: When was that factor most impactful? Was it during the CSAT grant funding period, after the CSAT funding ended, or throughout?

19H. Were there other barriers during or after the grant period?

🞏 No
🞏 Yes

IF YES, please describe: When was that factor most impactful? Was it during the CSAT grant funding period, after the CSAT funding ended, or throughout?

20. Do you have a copy of the A-CRA manual? *[IF NEEDED, PROMPT: If you have access to a shared copy of the manual through your organization, electronically, etc. that counts as having a copy.]*

🞏 No – ***SKIP TO QUESTION 22***
🞏 Yes - ***CONTINUE***

21. In the past six-months, how often would you say you used the manual?

*[IF NEEDED, PROMPT: By use, we mean a reference to the manual. It could mean a quick review of key session content in preparation for a session, or a thorough read-through in order to master the material.]*

🞏 Never
🞏 A few times per year, or less
🞏 About once a month
🞏 A few times per month
🞏 Weekly
🞏 Daily

22. In the past six-months, how often do you receive group clinical supervision?

*[IF NEEDED, PROMPT:  This includes any supervision you received; not supervision you provided. Our focus is on A-CRA supervision but you can include any supervision where A-CRA would be discussed – it does not need to be exclusively supervision for A-CRA cases.]*

🞏 Never
🞏 Once a week
🞏 Every two weeks
🞏 Monthly
🞏 Other, please describe how often:___________________________________

23. In the past six-months, how often do you receive individual clinical supervision?

*[IF NEEDED, PROMPT:  This includes any supervision you received; not supervision you provided. Our focus is on A-CRA supervision but you can include any supervision where A-CRA would be discussed – it does not need to be exclusively supervision for A-CRA cases.]*

🞏 Never
🞏 Once a week
🞏 Every two weeks
🞏 Monthly
🞏 Other, please describe how often:___________________________________

**IF QUESTION 22 = Never AND QUESTION 23 = Never, SKIP TO QUESTION 34**

**Which of the following happens during supervision sessions?**

24. My supervisor asks how my week has gone.

🞏 No
🞏 Yes

25. My supervisor discusses agency paperwork requirements.

🞏 No
🞏 Yes

26. My supervisor reviews my A-CRA case review report.

🞏 No
🞏 Yes

27. My supervisor asks me if I have any problem cases.

🞏 No
🞏 Yes

28. My supervisor reviews a recorded session with me and tells me what I have done well.

🞏 No
🞏 Yes

29. My supervisor reviews a recorded session with me and gives me suggestions about how I can improve my treatment delivery

🞏 No
🞏 Yes

30. My supervisor observes a live session and tells me what I have done well.

🞏 No
🞏 Yes

31. My supervisor observes a live session and gives me suggestions about how I can improve my treatment delivery

🞏 No
🞏 Yes

32. My supervisor asks me about my personal problems.

🞏 No
🞏 Yes

33. My supervisor role plays with me the correct way to do a procedure.

🞏 No
🞏 Yes

**Currently, when you are introducing A-CRA to a new client…**

34. How many sessions do you tell the client receiving A-CRA they will have?

_______0-99

🞏 LESS THAN 12
🞏 12 OR MORE

35. How many weeks do you tell the client receiving A-CRA the treatment will take?

_______0-99

🞏 LESS THAN 12 WEEKS
🞏 12 WEEKS OR MORE

36. How has the COVID-19 pandemic affected substance use treatment services at your organization?

37. How has it affected the ability of your organization to sustain A-CRA services?

38. What have been the most significant changes affecting your A-CRA program in response to COVID-19?

*[PROBE AS NEEDED: Changes could include things like…*

*…Changes in referrals to A-CRA*

*…Changes in how A-CRA assessment, treatment, or case management services are delivered*

*…Changes in staffing*

*…Remote work*

*…Telehealth service delivery*

*Were there other changes? If so, please describe: ]*

39. Have there been changes made in response to COVID-19 that have been beneficial, and will those changes continue beyond the pandemic?

*[PROBE AS NEEDED: These may be some of the changes you already described, or may be different. Changes could include things like…*

*…Changes in referrals to A-CRA*

*…Changes in how A-CRA assessment, treatment, or case management services are delivered*

*…Changes in staffing*

*…Remote work*

*…Telehealth service delivery*

*Were there other beneficial changes? If so, please describe: ]*

**OK, we are almost done. Next, I want to ask you about your general impressions regarding the SAMHSA CSAT-funded A-CRA project and then have a few questions about you.**

40. If you had a chance to participate in a SAMHSA CSAT project again, would you consider it?

🞏 No
🞏 Yes

41. Why/why not?

42. Is there anything you would change about the SAMHSA CSAT project in order to improve the sustainment of A-CRA at your agency?

43. Is there anything else you would like to share related to the topic of sustaining A-CRA at your agency?

[TURN OFF RECORDER]

That's all the interview questions I have. Thank you for providing this information. We will soon be sending you the link to the web survey in order to provide a more complete picture of the treatment offered to youth at your organization and about organizational and clinical support. We will send it within a few days, and will send your $50 Amazon e-gift card as soon as the survey is complete.

[IF PARTICIPANT WILL BE INTERVIEWED AGAIN, complete the tracking module beginning on the final page]

**Non-Sustainer Interview – CLINICIAN**

1. Based on the background information we’ve received, it sounds like your agency currently does not deliver the Adolescent Community Reinforcement Approach or A-CRA anymore. Is this correct?

🞏 No – **SWITCH TO SUSTAINER INTERVIEW**
🞏 Yes - ***CONTINUE***

2. Tell me a little bit about the substance use services at your agency. What services are available? What types of clients do you serve? (e.g., age range, services for specific substances).

3. What youth age range do you use A-CRA with at your agency? Throughout this interview, by “youth” we typically mean ages 12 through 17 (or through age 24 if your A-CRA training included young adults).

4. What is your primary professional role(s) at your clinic/site?

🞏 Clinical Supervisor – ***SWITCH TO SUPERVISOR ONLY VERSION***

🞏 Counselor or Clinician - ***CONTINUE***
🞏 Clinical Supervisor and Counselor/Clinician ***– SWITCH TO SUPERVISOR + CLINICIAN VERSION***

🞏 Other, please describe: ____________________(e.g., administrator, grant manager) ***- SWITCH TO SUPERVISOR VERSION***

5. Did you participate in any SAMHSA/CSAT-funded A-CRA training initiatives in your state? Yes or No?

🞏 No – ***SKIP TO QUESTION 6***🞏 Yes – ***CONTINUE***

**IF QUESTION 5 = Yes**

5A. Between what dates did you work on the project? Your best guess is fine.

Enter Start Date (MM/YY)

Enter End Date (MM/YY)

6. What role(s) did you serve while A-CRA was being delivered at your agency? ***[MARK ALL THAT APPLY]***

🞏 A-CRA Therapist
🞏 A-CRA Supervisor
🞏 Other (Please describe: ____________________________________________________)
🞏 No Role in delivering ACRA

7. Have you received any A-CRA certifications? This means that you completed all training requirements (such as coaching and submission of recorded sessions) and received written documentation of the certification.

**[*MARK ALL THAT APPLY*]**

🞏 First-level clinician certification (passed 9 A-CRA procedures)
🞏 Full clinician certification (passed all 19 A-CRA procedures)
🞏 Supervisor certification

🞏 A-CRA-TAY (Transitional Age Youth) certification

🞏 No, never certified in A-CRA

*[IF NEEDED, PROMPT:  A-CRA supervisor certification is different from A-CRA certification.  It refers to being certified to provide A-CRA clinical supervision].*

8. What do you think of A-CRA as a treatment for youth?

9. When did you/your agency stop delivering A-CRA? If you do not know the exact date, please give your best estimate.

Month/Year:______ /______

10. What were the main reasons you/your agency stopped delivering A-CRA?

11. What would have increased your desire to continue delivering A-CRA?

12. What would have increased your ability to continue delivering A-CRA?

13. Does your program currently offer any different types of treatment for youth with substance use disorders besides A-CRA?

🞏 No – ***SKIP TO QUESTION 20***
🞏 Yes - ***CONTINUE***
🞏 Don’t Know – ***SKIP TO QUESTION 20***

IF QUESTION 13= YES

14. What are those treatments called?

*[IF NEEDED, PROMPT: Examples of other treatments include Motivational Enhancement Therapy (MET), Cognitive Behavioral Therapy (CBT), Multidimensional Family Therapy (MDFT), 12-step facilitation, and supportive counseling as well as medication treatments like Suboxone or Naltrexone.*

*OR you can just describe the treatment. Is it outpatient? How long does it last? # of sessions/days/months?]*

20. Tell me about any ways in which your organization collaborates with other organizations – especially anything related to A-CRA. *[IF NEEDED, PROMPT: For example, you may have engaged in training or support activities with your state substance use services authority and/or Chestnut Health Systems, or have partnered with universities or research institutions on research related to A-CRA].*

21. Was there any planning done to ensure resources were available to continue A-CRA beyond the initial funding period? By resources, we mean things needed to support A-CRA. For example, this could include things like money, staff, supervision, training, and A-CRA manuals.

[*IF NEEDED, PROMPT: By strategic planning, we mean the process by which an organization defines its strategy or direction and makes decisions about how to allocate resources to pursue the strategy.]*

22. Can you please describe any policies that supported A-CRA delivery? These could include external policies, such as funding source and billing rules that support A-CRA, as well as internal policies at your organization.

*[IF NEEDED, PROMPT: By policies, we mean either organizational, state, national, or local policies. Here are some examples:*

*-State/county/health insurance co requires that we use an evidence based treatment (EBT) and A-CRA is an EBT*

*-Our agency’s mission emphasizes the involvement of the family, A-CRA does that]*

23. What about policies (external or internal) that interfered with A-CRA delivery?

*[IF NEEDED, PROMPT:  By policies, we mean either organizational, state, national, or local policies. Here are some examples:*

*-if a state decided to require that substance use clinics deliver a different treatment (other than A-CRA), this policy might interfere with the delivery of A-CRA.*

*- if certain insurance plans stopped reimbursing for A-CRA or the full number of sessions, this policy would interfere with A-CRA delivery.]*

24. Did A-CRA meet the needs of the populations you serve? How so or why not?

25. Can you tell me about any pressure your organization experienced to continue delivering A-CRA or to discontinue its use? *[IF NEEDED, PROMPT:  Pressure to deliver A-CRA could come from a variety of sources. For example, organizations might experience pressure if there is some financial incentive to deliver A-CRA. If a state department of health decides its mission is to deliver evidence based treatment for substance use, this could also be seen as pressure.]*

26. Would you say staff were supportive or reluctant to use A-CRA? Can you give me an example of what people have said or done to indicate their supportiveness or reluctance toward A-CRA?

27. Next, I would like to know what factors helped efforts to implement and sustain A-CRA in your organization. Please let me know if these factors were important at your organization, and if yes, how so. *[PROBE AS NEEDED: What factors were most helpful in that domain? Can you give me some examples? Were there important changes in which factors were most helpful once funding ended?]*

27A. Would you say characteristics of A-CRA helped efforts to implement and sustain A-CRA in your organization?  Yes or No?

*[IF NEEDED: for example, treatment content, structure of sessions, etc.]*

🞏 No
🞏 Yes

IF YES, please describe: When was that factor most impactful? Was it during the CSAT grant funding period, after the CSAT funding ended, or throughout?

27B. Would you say characteristics of key individuals helped efforts to implement and sustain A-CRA in your organization?  Yes or No?

*[IF NEEDED: “key individuals” are people who had a major positive impact on A-CRA implementation; could be within or outside of your treatment organization]*

🞏 No
🞏 Yes

IF YES, please describe: When was that factor most impactful? Was it during the CSAT grant funding period, after the CSAT funding ended, or throughout?

27C. Would you say client perspectives on A-CRA helped efforts to implement and sustain A-CRA in your organization?  Yes or No?

*[IF NEEDED: for example, if clients found A-CRA acceptable, appropriate, feasible, etc.]*

🞏 No
🞏 Yes

IF YES, please describe: When was that factor most impactful? Was it during the CSAT grant funding period, after the CSAT funding ended, or throughout?

27D. Would you say helpful factors within your organization helped efforts to implement and sustain A-CRA in your organization?  Yes or No?

*[IF NEEDED: for example, organizational leadership, staffing patterns, scheduling appointments, etc.]*

🞏 No
🞏 Yes

IF YES, please describe: When was that factor most impactful? Was it during the CSAT grant funding period, after the CSAT funding ended, or throughout?

27E. Would you say helpful factors outside your organization helped efforts to implement and sustain A-CRA in your organization?  Yes or No?

*[IF NEEDED: for example, state leadership, federal support, community* ***factors]***

🞏 No
🞏 Yes

IF YES, please describe: When was that factor most impactful? Was it during the CSAT grant funding period, after the CSAT funding ended, or throughout?

27F. Would you say partnerships with other organizations helped efforts to implement and sustain A-CRA in your organization?  Yes or No?

🞏 No
🞏 Yes

IF YES, please describe: When was that factor most impactful? Was it during the CSAT grant funding period, after the CSAT funding ended, or throughout?

27G. Would you say funding helped efforts to implement and sustain A-CRA in your organization?  Yes or No?

🞏 No
🞏 Yes

IF YES, please describe: When was that factor most impactful? Was it during the CSAT grant funding period, after the CSAT funding ended, or throughout?

27H. Were there other helpful factors during or after the grant period?

🞏 No
🞏 Yes

IF YES, please describe: When was that factor most impactful? Was it during the CSAT grant funding period, after the CSAT funding ended, or throughout?

28. Now, I’m going ask how the same six categories of factors may have included barriers that hindered efforts to implement and sustain A-CRA in your organization. Please let me know if these factors were important at your organization, and if yes, how so.

*[PROBE AS NEEDED: What factors were the biggest barriers in that domain? Can you give me some examples? Were there important changes in which factors were the biggest barriers once funding ended?]*

28A. Would you say characteristics of A-CRA hindered efforts to implement and sustain A-CRA in your organization? Yes or No?

*[IF NEEDED: for example, treatment content, structure of sessions, etc.]*

🞏 No
🞏 Yes

IF YES, please describe: When was that factor most impactful? Was it during the CSAT grant funding period, after the CSAT funding ended, or throughout?

28B. Would you say challenges with key individuals hindered efforts to implement and sustain A-CRA in your organization? Yes or No?

*[IF NEEDED: “key individuals” are people who presented major challenges for A-CRA implementation; could be within or outside of your treatment organization]*

🞏 No
🞏 Yes

IF YES, please describe: When was that factor most impactful? Was it during the CSAT grant funding period, after the CSAT funding ended, or throughout?

28C. Would you say client perspectives on A-CRA hindered efforts to implement and sustain A-CRA in your organization? Yes or No?

*[IF NEEDED: for example, if clients found A-CRA acceptable, appropriate, feasible, etc.]*

🞏 No
🞏 Yes

IF YES, please describe: When was that factor most impactful? Was it during the CSAT grant funding period, after the CSAT funding ended, or throughout?

28D. Would you say challenges within your organization hindered efforts to implement and sustain A-CRA in your organization? Yes or No?

*[IF NEEDED: for example, organizational leadership, staffing patterns, scheduling appointments, etc.]*

🞏 No
🞏 Yes

IF YES, please describe: When was that factor most impactful? Was it during the CSAT grant funding period, after the CSAT funding ended, or throughout?

28E. Would you say challenges outside your organization hindered efforts to implement and sustain A-CRA in your organization? Yes or No?

*[IF NEEDED: for example, state leadership, federal support, community factors]*

🞏 No
🞏 Yes

IF YES, please describe: When was that factor most impactful? Was it during the CSAT grant funding period, after the CSAT funding ended, or throughout?

28F. Would you say issues in partnerships with other organizations hindered efforts to implement and sustain A-CRA in your organization? Yes or No?

🞏 No
🞏 Yes

IF YES, please describe: When was that factor most impactful? Was it during the CSAT grant funding period, after the CSAT funding ended, or throughout?

28G. Would you say funding hindered efforts to implement and sustain A-CRA in your organization? Yes or No?

🞏 No
🞏 Yes

IF YES, please describe: When was that factor most impactful? Was it during the CSAT grant funding period, after the CSAT funding ended, or throughout?

28H. Were there other barriers during or after the grant period?

🞏 No
🞏 Yes

IF YES, please describe: When was that factor most impactful? Was it during the CSAT grant funding period, after the CSAT funding ended, or throughout?

29. Have you ever had a copy of the A-CRA manual? *[IF NEEDED, PROMPT: If you have access to a shared copy of the manual through your organization, electronically, etc. that counts as having a copy.]*

🞏 No - ***SKIP TO QUESTION 33***🞏 Yes - ***CONTINUE***

**IF QUESTION 29 = Yes**

30. Please think about the six-month period, right before A-CRA treatment delivery ended. How often did you use the manual?

*[IF NEEDED, PROMPT: By use, we mean a reference to the manual. It could mean a quick review of key session content in preparation for a session, or a thorough read-through in order to master the material.]*

🞏 Never
🞏 A few times per year, or less
🞏 About once a month
🞏 A few times per month
🞏 Weekly
🞏 Daily

**IF QUESTION 29 = Yes**

31. Do you still use your manual?

*[IF NEEDED, PROMPT: By use, we mean a reference to the manual. It could mean a quick review of key session content in preparation for a session, or a thorough read-through in order to master the material.]*

🞏 No – ***SKIP TO QUESTION 33***🞏 Yes - ***CONTINUE***

**IF QUESTION 31 = Yes**

32. How often do you use your manual now?

*[IF NEEDED, PROMPT: By use, we mean a reference to the manual. It could mean a quick review of key session content in preparation for a session, or a thorough read-through in order to master the material.]*

🞏 Never
🞏 A few times per year, or less
🞏 About once a month
🞏 A few times per month
🞏 Weekly
🞏 Daily

**For the next few questions, please think about the six-month period, right before A-CRA treatment delivery ended.**

33. How often did you receive group clinical supervision?

*[IF NEEDED, PROMPT:  This includes any supervision you received; not supervision you provided. Our focus is on A-CRA supervision but you can include any supervision where A-CRA would be discussed – it does not need to be exclusively supervision for A-CRA cases.]*

🞏 Never
🞏 Once a week
🞏 Every two weeks
🞏 Monthly
🞏 Other, please describe how often:___________________________________

34. Again, please think about the six-month period, right before A-CRA treatment delivery ended. How often did you receive individual clinical supervision?

*[IF NEEDED, PROMPT:  This includes any supervision you received; not supervision you provided. Our focus is on A-CRA supervision but you can include any supervision where A-CRA would be discussed – it does not need to be exclusively supervision for A-CRA cases.]*

🞏 Never
🞏 Once a week
🞏 Every two weeks
🞏 Monthly
🞏 Other, please describe how often:___________________________________

**IF QUESTION 33 = Never AND QUESTION 34 = Never, SKIP TO QUESTION 45**

**Thinking about the six-month period, right before A-CRA treatment delivery ended, which of the following happened during supervision sessions?**

35. My supervisor asked how my week had gone.

🞏 No
🞏 Yes

36. (During the six-month period, right before A-CRA treatment delivery ended), my supervisor discussed agency paperwork requirements.

🞏 No
🞏 Yes

37. (During the six-month period, right before A-CRA treatment delivery ended), my supervisor reviewed my A-CRA case review report.

🞏 No
🞏 Yes

38. (During the six-month period, right before A-CRA treatment delivery ended), my supervisor asked me if I had any problem cases.

🞏 No
🞏 Yes

39. (During the six-month period, right before A-CRA treatment delivery ended), my supervisor reviewed a recorded session with me and told me what I did well.

🞏 No
🞏 Yes

40. (During the six-month period, right before A-CRA treatment delivery ended), my supervisor reviewed a recorded session with me and gave me suggestions about how I could improve my treatment delivery

🞏 No
🞏 Yes

41. During the six-month period, right before A-CRA treatment delivery ended, my supervisor observed a live session and told me what I did well.

🞏 No
🞏 Yes

42. (During the six-month period, right before A-CRA treatment delivery ended), my supervisor observed a live session and gave me suggestions about how I could improve my treatment delivery

🞏 No
🞏 Yes

43. (During the six-month period, right before A-CRA treatment delivery ended), my supervisor asked me about my personal problems.

🞏 No
🞏 Yes

44. During the six-month period, right before A-CRA treatment delivery ended, my supervisor role played with me the correct way to do a procedure.

🞏 No
🞏 Yes

**Now I’d like to ask you the same questions, but now please think about the current practices in the adolescent treatment program.**

45. How often do you receive group clinical supervision?

*[IF NEEDED, PROMPT:  This includes any supervision you received; not supervision you provided. Our focus is on A-CRA supervision but you can include any supervision where A-CRA would be discussed – it does not need to be exclusively supervision for A-CRA cases.]*

🞏 Never
🞏 Once a week
🞏 Every two weeks
🞏 Monthly
🞏 Other, please describe how often:___________________________________

46. How often do you receive individual clinical supervision?

*[IF NEEDED, PROMPT:  This includes any supervision you received; not supervision you provided. Our focus is on A-CRA supervision but you can include any supervision where A-CRA would be discussed – it does not need to be exclusively supervision for A-CRA cases.]*

🞏 Never
🞏 Once a week
🞏 Every two weeks
🞏 Monthly
🞏 Other, please describe how often:___________________________________

***IF QUESTION 45= Never AND QUESTION 46 = Never, SKIP TO QUESTION 56***

**Thinking about the current practices in your adolescent treatment program, which of the following happens during supervision sessions?**

47. My supervisor asks me how my week has gone.

🞏 No
🞏 Yes

48. My supervisor discusses agency paperwork requirements.

🞏 No
🞏 Yes

49. My supervisor asks me if I have any problem cases.

🞏 No
🞏 Yes

50. My supervisor reviews a recorded session with me and tells me what I have done well.

🞏 No
🞏 Yes

51. My supervisor reviews a recorded session with me and gives me suggestions about how I could improve my treatment delivery.

🞏 No
🞏 Yes

52. My supervisor observes a live session and tells me what I have done well.

🞏 No
🞏 Yes

53. My supervisor observes a live session and gives me suggestions about how I could improve my treatment delivery.

🞏 No
🞏 Yes

54. My supervisor asks me about my personal problems.

🞏 No
🞏 Yes

55. My supervisor role plays with me the correct way to deliver therapy.

🞏 No
🞏 Yes

56. During the six month period, right before A-CRA treatment delivery ended, how many sessions did you tell the client receiving A-CRA they would have?

_______0-99

🞏 LESS THAN 12
🞏 12 OR MORE

57. During the six month period, right before A-CRA treatment delivery ended, how many weeks did you tell the client receiving A-CRA the treatment would take?

_______0-99

🞏 LESS THAN 12 WEEKS
🞏 12 WEEKS OR MORE

58. How has the COVID-19 pandemic affected substance use treatment services at your organization?

59. When the pandemic began in March 2020, was your organization still delivering A-CRA?

🞏 No – ***SKIP TO QUESTION 62***
🞏 Yes – ***CONTINUE***

🞏 Don’t know – ***SKIP TO QUESTION 62***

**IF QUESTION 59 = YES**

60. What have been the most significant changes affecting your A-CRA program in response to COVID-19?

*[PROBE AS NEEDED: Changes could include things like…*

*…A-CRA was discontinued due to COVID-related changes*

*…Changes in referrals to A-CRA*

*…Changes in how A-CRA assessment, treatment, or case management services are delivered*

*…Changes in staffing*

*…Remote work*

*…Telehealth service delivery*

*Were there other changes? If so, please describe: ]*

**IF QUESTION 59 = YES**

61. Have there been changes made in response to COVID-19 that have been beneficial, and will those changes continue beyond the pandemic?

*[PROBE AS NEEDED: These may be some of the changes you already described, or may be different. Changes could include things like…*

*…Changes in referrals to A-CRA*

*…Changes in how A-CRA assessment, treatment, or case management services are delivered*

*…Changes in staffing*

*…Remote work*

*…Telehealth service delivery*

*Were there other beneficial changes? If so, please describe: ]*

**OK, we are almost done. Next, I want to ask you about your general impressions regarding the SAMHSA CSAT-funded A-CRA project and then have a few questions about you.**

62. If you had a chance to participate in a SAMHSA CSAT project again, would you consider it?

🞏 No
🞏 Yes

63. Why/why not?

64. Is there anything you would change about the SAMHSA CSAT project in order to improve the sustainment of A-CRA at your agency?

65. Is there anything else you would like to share related to the topic of sustaining A-CRA at your agency?

[TURN OFF RECORDER]

That's all the interview questions I have. Thank you for providing this information. We will soon be sending you the link to the web survey in order to provide a more complete picture of the treatment offered to youth at your organization and about organizational and clinical support. We will send it within a few days, and will send your $50 Amazon e-gift card as soon as the survey is complete.

[IF PARTICIPANT WILL BE INTERVIEWED AGAIN, complete the tracking module beginning on the final page]

***CLINICIAN/SUPERVISOR INTERVIEW VERBAL CONSENT***

(READ PRIOR TO BEGINNING INTERVIEW/RECORDING)

Hi [CLINICIAN/SUPERVISOR NAME],

This is [INTERVIEWER NAME] calling from RAND to speak with you about the youth substance use treatment program where you work and any experiences you have with implementing the Adolescent Community Reinforcement Approach (A-CRA) treatment model. Is this still a good time to complete your interview? To ensure confidentiality, make sure you are in a private, secure location for completing the interview.

>IF NO, RESCHEDULE.

>IF YES, great.

We would like a clinician and/or clinical supervisor for youth substance use treatment to answer these questions. Ideally, it should be someone knowledgeable about your A-CRA program. Are you the right person at your organization to participate in this opportunity?

>IF NO: May I please have the contact information of the best contact(s) for youth substance use treatment? [RECORD CONTACT INFORMATION TO FOLLOW-UP]

>IF YES, thanks for confirming.

Before we begin, let me assure you that your responses to these questions will be held in strict confidence. In collaboration with Chestnut Health Systems, we are requesting interviews and surveys from clinicians and supervisors from nearly 20 states that received A-CRA training from Chestnut Health, as part of SAMHSA Center of Substance Abuse Treatment (CSAT) grants received by state substance use services authorities. We will use information from the interviews and surveys to understand how different CSAT funding models influence the sustainability of A-CRA delivery after funding ends. We will also examine information provided by state organizations that received the CSAT grants and data already collected from prior CSAT grantee organizations that implemented A-CRA. We will not attribute comments to specific individuals or programs in any of our reports or publications. Your responses will not be shared with your organization or with SAMHSA.

Today’s interview will last up to 45 minutes. Afterwards, we will ask you to complete a 30-minute online survey on your own to give us a more complete picture. You will receive a $50 Amazon electronic gift card upon completion of the web survey as a thank you for your participation.

Your participation in this discussion and the survey is entirely voluntary. We would like to have your responses to all of the questions. However, if you’re uncomfortable with any question we can skip it, and you can stop the interview at any time. There are no right or wrong answers – we are interested in your perspectives and experiences. Finally, we would like to audio-record the interview to ensure that we capture everything that is said. We will destroy the recording once we confirm we have captured everything in our de-identified notes and transcripts. However, you can still participate in the interview even if you do not give permission to audio-record.

If you have any questions or want to discuss the project further at any time, you may always contact us at 360-578-2911 or at pham@rand.org. Furthermore, if you have questions about your rights as a research participant or need to report a research-related injury or concern, you can contact RAND's Human Subjects Protection Committee toll-free at (866) 697-5620 or email [hspcinfo@​rand.org](mailto:hspcinfo@​rand.org)​. If you contact the Committee, please reference Study #2020-N0887.

- **Do you have any questions?**
- **Are you willing to take part in this discussion?**

>IF NO: That is not a problem, thank you for your time.

>IF YES, great.

- **Is it ok with you if we audio-tape this discussion?**

>IF NO: That is not a problem. I can take notes while we’re talking so I don’t miss anything important, though that means we might proceed through the interview more slowly than usual. I could also arrange for a colleague to take notes during the interview. [RESCHEDULE IF NEEDED]

>IF YES, perfect, let’s get started.

- **[IF UNKNOWN] Based on the background information we’ve received, I am not sure whether your organization currently delivers A-CRA. Can you confirm whether or not A-CRA is delivered by clinicians at your organization?**

>IF NO: BEGIN NON-SUSTAINER INTERVIEW

>IF YES: BEGIN SUSTAINER INTERVIEW

**Sustainer Interview – CLINICAL SUPERVISOR & CLINICIAN**

1. Based on the background information we’ve received, it sounds like your agency currently delivers the Adolescent Community Reinforcement Approach or A-CRA. Is this correct?

🞏 No – ***SWITCH TO NON-SUSTAINER INTERVIEW***🞏 Yes - ***CONTINUE***

2. Tell me a little bit about the substance use services at your agency. What services are available? What types of clients do you serve? (e.g., age range, services for specific substances).

3. What youth age range do you use A-CRA with at your agency? Throughout this interview, by “youth” we typically mean ages 12 through 17 (or through age 24 if your A-CRA training included young adults).

4. What is your primary professional role(s) at your clinic/site?

🞏 Clinical Supervisor – ***SWITCH TO SUPERVISOR ONLY VERSION***

🞏 Counselor or Clinician - ***SWITCH TO CLINICIAN ONLY VERSION***
🞏 Clinical Supervisor and Counselor/Clinician ***– CONTINUE HERE WITH SUPERVISOR + CLINICIAN VERSION***

🞏 Other, please describe: ____________________(e.g., administrator, grant manager) ***- SWITCH TO SUPERVISOR VERSION***

5. Did you participate in any SAMHSA/CSAT-funded A-CRA training initiatives in your state? Yes or No?

*[IF NEEDED, PROMPT AS RELEVANT: Our records do/do not indicate that you received training in A-CRA as part of a SAMHSA CSAT grant under the [initiative] in [grants years]. (IF KNOWN) In your state, I believe this initiative was called [NAME]. So would it be accurate to say you did/did not participate in your state’s A-CRA training initiative?]*

🞏 No – ***SKIP TO QUESTION 6***🞏 Yes – ***CONTINUE***

**IF QUESTION 5 = YES**

5A. Between what dates did you work on the project? Your best guess is fine.

Enter Start Date (MM/YY)

Enter End Date (MM/YY)

6. What role(s) have you served in delivering A-CRA? ***[MARK ALL THAT APPLY]***

🞏 A-CRA Therapist
🞏 A-CRA Clinical Supervisor
🞏 Other (Please describe: ____________________________________________________)
🞏 No Role in delivering ACRA

7. Have you received any A-CRA certifications? This means that you completed all training requirements (such as coaching and submission of recorded sessions) and received written documentation of the certification.

***[MARK ALL THAT APPLY]***

*[IF NEEDED, PROMPT:  A-CRA supervisor certification is different from A-CRA certification.  It refers to being certified to provide A-CRA clinical supervision].*

🞏 First-level clinician certification (passed 9 A-CRA procedures)
🞏 Full clinician certification (passed all 19 A-CRA procedures)
🞏 Supervisor certification
🞏 A-CRA-TAY (Transitional Age Youth) certification

🞏 No, never certified in A-CRA

8. What do you think of A-CRA as a treatment for youth? Please note that throughout this interview, by “youth” we mean the population served during your SAMHSA CSAT grant periods.

9. Does your program currently offer any different types of treatment for youth with substance use disorders besides A-CRA?

🞏 No – ***SKIP TO QUESTION 11***
🞏 Yes - ***CONTINUE***
🞏 Don’t Know – ***SKIP TO QUESTION 11***

**IF QUESTION 9= YES**

10. What are those treatments called?

*[IF NEEDED, PROMPT: Examples of other treatments include Motivational Enhancement Therapy (MET), Cognitive Behavioral Therapy (CBT), Multidimensional Family Therapy (MDFT), 12-step facilitation, and supportive counseling as well as medication treatments like Suboxone or Naltrexone.*

*OR you can just describe the treatment. Is it outpatient? How long does it last? # of sessions/days/months?]*

11. Over the past 6 months, approximately how many youth received substance use treatment at your agency?

_______0-999

12. Approximately how many youth received A-CRA over the past 6 months?

_______0-999

13. Currently, how many clinicians at your agency treat youth with substance use problems?

_______0-999

14. Currently, how many of the clinicians at your agency have received an A-CRA certification?

*[IF NEEDED, PROMPT: By certified, we mean they completed all training requirements – such as coaching and submission of recorded sessions – and received written documentation of certification. This includes first-level, full, transitional age youth, and supervisor certifications.]*

_______0-999

15. How many clinicians deliver A-CRA at your agency?

_______0-999

16. Next, I’d like to ask you about your agency’s plan to continue A-CRA clinical supervision support.

Does your agency plan to maintain a Clinical Supervisor focused on A-CRA?

🞏 No – ***SKIP TO QUESTION 17***
🞏 Yes
🞏 Don’t Know

**IF QUESTION 16= YES**

16A. Does your agency plan to support Clinical Supervisor and Counselor time for supervision?

🞏 No
🞏 Yes
🞏 Don’t Know

**IF QUESTION 16= YES**

16B. Does your agency plan to support the Clinical Supervisor to listen to recorded therapy sessions and provide individualized feedback to counselors?

🞏 No
🞏 Yes
🞏 Don’t Know

17. Tell me about any ways in which your organization collaborates with other organizations to sustain A-CRA.

*[IF NEEDED, PROMPT: For example, you may have engaged in training or support activities with your state substance use services authority and/or Chestnut Health Systems, or have partnered with universities or research institutions on research related to A-CRA].*

18. Tell me about any planning that has been done to ensure resources are available to continue A-CRA. By resources, we mean things needed to support A-CRA. For example, this could include things like money, staff, supervision, training, and A-CRA manuals.

[*IF NEEDED, PROMPT: By strategic planning, we mean the process by which an organization defines its strategy or direction and makes decisions about how to allocate resources to pursue the strategy.]*

19. Can you please describe any policies that support A-CRA delivery? These could include external policies, such as funding source and billing rules that support A-CRA, as well as internal policies at your organization.

*[IF NEEDED, PROMPT: By policies, we mean either organizational, state, national, or local policies. Here are some examples:*

*-State/county/health insurance co requires that we use an evidence based treatment (EBT) and A-CRA is an EBT*

*-Our agency’s mission emphasizes involvement of the family, A-CRA does that]*

20. What about policies (external or internal) that interfere with A-CRA delivery?

*[IF NEEDED, PROMPT:  By policies, we mean either organizational, state, national, or local policies. Here are some examples:*

*-if a state decided to require that substance use clinics deliver a different treatment (other than A-CRA), this policy might interfere with the delivery of A-CRA.*

*- if certain insurance plans stopped reimbursing for A-CRA or the full number of sessions, this policy would interfere with A-CRA delivery.]*

21. Does A-CRA meet the needs of the populations you serve? How so or why not?

22. Can you tell me about any pressure your organization experienced to continue delivering A-CRA or to discontinue its use?

*[IF NEEDED, PROMPT: Pressure to deliver A-CRA could come from a variety of sources. For ex., organizations might experience pressure if there is some financial incentive to deliver A-CRA. If a state department of health decides its mission is to deliver evidence based treatment for substance use, this could also be seen as pressure.]*

23. Would you say staff are supportive or reluctant to use A-CRA? Can you give me an example of what people have said or done to indicate their supportiveness or reluctance toward A-CRA?

24. Next, I would like to know what factors helped efforts to implement and sustain A-CRA in your organization. Please let me know if these factors were important in your organization, and if yes, how so.

*[PROBE AS NEEDED: What factors were most helpful in that domain? Can you give me some examples? Were there important changes in which factors were most helpful once funding ended?]*

24A. Would you say characteristics of A-CRA helped efforts to implement and sustain A-CRA in your organization?  Yes or No?

*[IF NEEDED: for example, treatment content, structure of sessions, etc.]*

🞏 No
🞏 Yes

IF YES, please describe: When was that factor most impactful? Was it during the CSAT grant funding period, after the CSAT funding ended, or throughout?

24B. Would you say characteristics of key individuals helped efforts to implement and sustain A-CRA in your organization?  Yes or No?

*[IF NEEDED: “key individuals” are people who had a major positive impact on A-CRA implementation; could be within or outside of your treatment organization]*

🞏 No
🞏 Yes

IF YES, please describe: When was that factor most impactful? Was it during the CSAT grant funding period, after the CSAT funding ended, or throughout?

24C. Would you say client perspectives on A-CRA helped efforts to implement and sustain A-CRA in your organization?  Yes or No?

*[IF NEEDED: for example, if clients found A-CRA acceptable, appropriate, feasible, etc.]*

🞏 No
🞏 Yes

IF YES, please describe: When was that factor most impactful? Was it during the CSAT grant funding period, after the CSAT funding ended, or throughout?

24D. Would you say helpful factors within your organization helped efforts to implement and sustain A-CRA in your organization?  Yes or No?

*[IF NEEDED: for example, organizational leadership, staffing patterns, scheduling appointments, etc.]*

🞏 No
🞏 Yes

IF YES, please describe: When was that factor most impactful? Was it during the CSAT grant funding period, after the CSAT funding ended, or throughout?

24E. Would you say helpful factors outside your organization helped efforts to implement and sustain A-CRA in your organization?  Yes or No?

*[IF NEEDED: for example, state leadership, federal support, community factors]*

🞏 No
🞏 Yes

IF YES, please describe: When was that factor most impactful? Was it during the CSAT grant funding period, after the CSAT funding ended, or throughout?

24F. Would you say partnerships with other organizations helped efforts to implement and sustain A-CRA in your organization?  Yes or No?

🞏 No
🞏 Yes

IF YES, please describe: When was that factor most impactful? Was it during the CSAT grant funding period, after the CSAT funding ended, or throughout?

24G. Would you say funding helped efforts to implement and sustain A-CRA in your organization?  Yes or No?

🞏 No
🞏 Yes

IF YES, please describe: When was that factor most impactful? Was it during the CSAT grant funding period, after the CSAT funding ended, or throughout?

24H. Were there other helpful factors during or after the grant period?

🞏 No
🞏 Yes

IF YES, please describe: When was that factor most impactful? Was it during the CSAT grant funding period, after the CSAT funding ended, or throughout?

25. Now, I’m going ask how the same six categories of factors may have included barriers that hindered efforts to implement and sustain A-CRA in your organization. Please let me know if these factors were important in your organization, and if yes, how so.

*[PROBE AS NEEDED: What factors were the biggest barriers in that domain? Can you give me some examples? Were there important changes in which factors were the biggest barriers once funding ended?]*

25A. Would you say characteristics of A-CRA hindered efforts to implement and sustain A-CRA in your organization? Yes or No?

*[IF NEEDED: for example, treatment content, structure of sessions, etc.]*

🞏 No
🞏 Yes

IF YES, please describe: When was that factor most impactful? Was it during the CSAT grant funding period, after the CSAT funding ended, or throughout?

25B. Would you say challenges with key individuals hindered efforts to implement and sustain A-CRA in your organization? Yes or No?

*[IF NEEDED: “key individuals” are people who presented major challenges for A-CRA implementation; could be within or outside of your treatment organization]*

🞏 No
🞏 Yes

IF YES, please describe: When was that factor most impactful? Was it during the CSAT grant funding period, after the CSAT funding ended, or throughout?

25C. Would you say client perspectives on A-CRA hindered efforts to implement and sustain A-CRA in your organization? Yes or No?

*[IF NEEDED: for example, if clients found A-CRA acceptable, appropriate, feasible, etc.]*

🞏 No
🞏 Yes

IF YES, please describe: When was that factor most impactful? Was it during the CSAT grant funding period, after the CSAT funding ended, or throughout?

25D. Would you say challenges within your organization hindered efforts to implement and sustain A-CRA in your organization? Yes or No?

*[IF NEEDED: for example, organizational leadership, staffing patterns, scheduling appointments, etc.]*

🞏 No
🞏 Yes

IF YES, please describe: When was that factor most impactful? Was it during the CSAT grant funding period, after the CSAT funding ended, or throughout?

25E. Would you say challenges outside your organization hindered efforts to implement and sustain A-CRA in your organization? Yes or No?

*[IF NEEDED: for example, state leadership, federal support, community factors]*

🞏 No
🞏 Yes

IF YES, please describe: When was that factor most impactful? Was it during the CSAT grant funding period, after the CSAT funding ended, or throughout?

25F. Would you say issues in partnerships with other organizations hindered efforts to implement and sustain A-CRA in your organization? Yes or No?

🞏 No
🞏 Yes

IF YES, please describe: When was that factor most impactful? Was it during the CSAT grant funding period, after the CSAT funding ended, or throughout?

25G. Would you say funding hindered efforts to implement and sustain A-CRA in your organization? Yes or No?

🞏 No
🞏 Yes

IF YES, please describe: When was that factor most impactful? Was it during the CSAT grant funding period, after the CSAT funding ended, or throughout?

25H. Were there other barriers during or after the grant period?

🞏 No
🞏 Yes

IF YES, please describe: When was that factor most impactful? Was it during the CSAT grant funding period, after the CSAT funding ended, or throughout?

26. How many staff supervise clinicians who treat youth with substance use problems?

______0-999

27. Do you supervise clinicians who deliver A-CRA?

🞏 No
🞏 Yes
🞏 Don’t Know

28. How many others at your agency supervise clinicians delivering A-CRA?

______0-99

29. Have you personally certified any clinicians in A-CRA?

🞏 No –***SKIP TO QUESITON 46***
🞏 Yes - ***CONTINUE***

*[IF NEEDED, PROMPT: By certified, we mean they completed all training requirements – such as coaching and submission of recorded sessions – and received written documentation of certification. Respond “yes” if you have personally certified anyone with the first-level, full, transitional age youth, and/or supervisor certifications.]*

**IF QUESTION 29 = YES**

30. You mentioned that you personally had certified one or more clinicians in A-CRA at your organization. Can you please describe the certification process to me?

The clinician was asked to:

🞏 Read the A-CRA manual

🞏 Take an online A-CRA research course

🞏 Pass an A-CRA quiz with a score of 80% or higher

🞏 Attend a Chestnut or Robert J. Meyers A-CRA initial training OR attend an in-house training

🞏 Participate in regular coaching calls with Chestnut or regular supervision with in-house Clinical Supervisor (respondent) during certification (regular= @ every other week)

🞏 Regularly record therapy sessions for in-house Clinical Supervisor (respondent) review/Chestnut’s review (regular= at least some sessions weekly))

🞏 Demonstrate competency in General Clinical Skills on the DSRs

🞏 Demonstrate competency in the following A-CRA procedures:

Functional Analysis of Use

Functional Analysis of Pro-social behavior

Happiness Scale

Treatment Plan/Goals of Counseling

Communication Skills

Problem Solving Skills

Adolescent-Caregiver Relationship Skills, and

Homework based of 3 or better on all components of a given procedure using the A-CRA rating manual?

If in-house training was provided, did it:

🞏 Include didactic information about A-CRA procedures?

🞏 Modeling or review of audio recordings of procedures that were well done

🞏 The opportunity to role play procedures

🞏 Other, please explain_____________________________________

**Next, I'm going to ask you a series of questions about how the certification process currently works in your agency. Please respond by saying "True," "False," or "don't know" if you are unsure.**

31. When I decide to pass a clinician on a procedure it is based on ratings of 1 or more on every component of a procedure. Remember that each component of a procedure is rated on a 1 to 5 scale.

🞏 False
🞏 True
🞏 Don’t Know

32. Communication skills is a procedure that people have to pass to attain certification.

🞏 False
🞏 True
🞏 Don’t Know

33. I review recorded sessions during the certification process.

🞏 False
🞏 True
🞏 Don’t Know

34. I am required to sit in sessions with clinicians during the certification process.

🞏 False
🞏 True
🞏 Don’t Know

35. Clinicians record one or two of their sessions.

🞏 False
🞏 True
🞏 Don’t Know

36. I refer to the A-CRA rating manual when rating session recordings.

🞏 False
🞏 True
🞏 Don’t Know

37. Clinicians are required to take a knowledge test as part of the certification process.

🞏 False
🞏 True
🞏 Don’t Know

38. Clinicians are not required to pass General Clinical Skills as part of the certification process.

🞏 False
🞏 True
🞏 Don’t Know

39. Each clinician has a certification workbook.

🞏 False
🞏 True
🞏 Don’t Know

40. I complete the A-CRA checklist when I am listening to a recorded session during or after the certification process.

🞏 False
🞏 True
🞏 Don’t Know

41. Clinicians are required to read the A-CRA manual during the training process.

🞏 False
🞏 True
🞏 Don’t Know

42. Time is set aside for training clinicians in A-CRA

🞏 False
🞏 True
🞏 Don’t Know

43. During training clinicians are required to practice procedures with role-plays.

🞏 False
🞏 True
🞏 Don’t Know

44. Adolescent-Caregiver Relationship Skills is one of the procedures for basic certification.

🞏 False
🞏 True
🞏 Don’t Know

45. It doesn't matter if clinicians show competency in all of the additional procedures as well.

🞏 False
🞏 True
🞏 Don’t Know

46. On average, approximately how many session recordings do clinicians at your site complete to reach first-level certification (pass first 9 A-CRA procedures)? Your best guess is fine.

______0-99

47. Do you have a copy of the A-CRA manual? *[IF NEEDED, PROMPT: If you have access to a shared copy of the manual through your organization, electronically, etc. that counts as having a copy.]*

🞏 No – ***SKIP TO QUESTION 49***
🞏 Yes - ***CONTINUE***

48. In the past six-months, how often would you say you used the manual?

*[IF NEEDED, PROMPT: By use, we mean a reference to the manual. It could mean a quick review of key session content in preparation for a session, or a thorough read-through in order to master the material.]*

🞏 Never
🞏 A few times per year, or less
🞏 About once a month
🞏 A few times per month
🞏 Weekly
🞏 Daily

**For the next few questions, please think about the six-month period, right before A-CRA treatment delivery ended.**

49. In the past six-months, how often do you provide group clinical supervision?

*[IF NEEDED, PROMPT:  This includes any supervision you provided; not supervision you received. Our focus is on A-CRA supervision but you can include any supervision where A-CRA would be discussed – it does not need to be exclusively supervision for A-CRA cases.]*

🞏 Never
🞏 Once a week
🞏 Every two weeks
🞏 Monthly
🞏 Other, please describe how often:___________________________________

50. In the past six-months, how often do you provide individual clinical supervision?

*[IF NEEDED, PROMPT: This includes any supervision you provided; not supervision you received. Our focus is on A-CRA supervision but you can include any supervision where A-CRA would be discussed – it does not need to be exclusively supervision for A-CRA cases.]*

🞏 Never
🞏 Once a week
🞏 Every two weeks
🞏 Monthly
🞏 Other, please describe how often:___________________________________

**IF QUESTION 49 = Never AND QUESTION 50 = Never, SKIP TO QUESTION 61**

**Which of the following happens during supervision sessions?**

51. My supervisor asks how my week has gone.

🞏 No
🞏 Yes

52. My supervisor discusses agency paperwork requirements.

🞏 No
🞏 Yes

53. My supervisor reviews my A-CRA case review report.

🞏 No
🞏 Yes

54. My supervisor asks me if I have any problem cases.

🞏 No
🞏 Yes

55. My supervisor reviews a recorded session with me and tells me what I have done well.

🞏 No
🞏 Yes

56. My supervisor reviews a recorded session with me and gives me suggestions about how I can improve my treatment delivery

🞏 No
🞏 Yes

57. My supervisor observes a live session and tells me what I have done well.

🞏 No
🞏 Yes

58. My supervisor observes a live session and gives me suggestions about how I can improve my treatment delivery

🞏 No
🞏 Yes

59. My supervisor asks me about my personal problems.

🞏 No
🞏 Yes

60. My supervisor role plays with me the correct way to do a procedure.

🞏 No
🞏 Yes

61. How many new clinicians have been trained in A-CRA in the past six-months in the organization?

______0-999

62. How many new clinicians have been trained in A-CRA in the past six-months at a Chestnut or Robert J. Meyers training?

______0-999

**[IF ANSWER TO QUESITON 61 > or = to ANSWER TO QUESTION 62 CONTINUE; OTHERWISE, SKIP TO QUESTION 65]**

**IF ANSWER TO QUESTION 61 >= ANSWER TO QUESTION 62**

63. Are there training agendas for your trainings?

🞏 No ***SKIP TO QUESTION 65***
🞏 Yes ***CONTINUE***

**IF QUESTION 63=Yes AND ANSWER TO QUESTION 61>0 AND ANSWER TO QUESTION 61 >= ANSWER TO QUESTION 62**

64. Will you please send me (email, mail, fax) a copy of the agenda?

🞏 No
🞏 Yes

**Currently, when you are introducing A-CRA to a new client…**

65. How many sessions do you tell the client receiving A-CRA they will have?

_______0-99

🞏 LESS THAN 12
🞏 12 OR MORE

66. How many weeks do you tell the client receiving A-CRA the treatment will take?

_______0-99

🞏 LESS THAN 12 WEEKS
🞏 12 WEEKS OR MORE

67. How has the COVID-19 pandemic affected substance use treatment services at your organization?

68. How has it affected the ability of your organization to sustain A-CRA services?

69. What have been the most significant changes affecting your A-CRA program in response to COVID-19?

*[PROBE AS NEEDED: Changes could include things like…*

*…Changes in referrals to A-CRA*

*…Changes in how A-CRA assessment, treatment, or case management services are delivered*

*…Changes in staffing*

*…Remote work*

*…Telehealth service delivery*

*Were there other changes? If so, please describe: ]*

70. Have there been changes made in response to COVID-19 that have been beneficial, and will those changes continue beyond the pandemic?

*[PROBE AS NEEDED: These may be some of the changes you already described, or may be different. Changes could include things like…*

*…Changes in referrals to A-CRA*

*…Changes in how A-CRA assessment, treatment, or case management services are delivered*

*…Changes in staffing*

*…Remote work*

*…Telehealth service delivery*

*Were there other beneficial changes? If so, please describe: ]*

**OK, we are almost done. Next, I want to ask you about your general impressions regarding the SAMHSA CSAT-funded A-CRA project and then have a few questions about you.**

71. If you had a chance to participate in a SAMHSA CSAT project again, would you consider it?

🞏 No
🞏 Yes

72. Why/why not?

73. Is there anything you would change about the SAMHSA CSAT project in order to improve the sustainment of A-CRA at your agency?

74. Is there anything else you would like to share related to the topic of sustaining A-CRA at your agency?

[TURN OFF RECORDER]

That's all the interview questions I have. Thank you for providing this information. We will soon be sending you the link to the web survey in order to provide a more complete picture of the treatment offered to youth at your organization and about organizational and clinical support. We will send it within a few days, and will send your $50 Amazon e-gift card as soon as the survey is complete.

[IF PARTICIPANT WILL BE INTERVIEWED AGAIN, complete the tracking module beginning on the final page]

**Non-Sustainer Interview – CLINICAL SUPERVISOR & CLINICIAN**

1. Based on the background information we’ve received, it sounds like your agency currently does not deliver the Adolescent Community Reinforcement Approach or A-CRA anymore. Is this correct?

🞏 No – **SWITCH TO SUSTAINER INTERVIEW**
🞏 Yes - ***CONTINUE***

2. Tell me a little bit about the substance use services at your agency. What services are available? What types of clients do you serve? (e.g., age range, services for specific substances).

3. What youth age range do you use A-CRA with at your agency? Throughout this interview, by “youth” we typically mean ages 12 through 17 (or through age 24 if your A-CRA training included young adults).

4. What is your primary professional role(s) at your clinic/site?

🞏 Clinical Supervisor – ***SWITCH TO SUPERVISOR ONLY VERSION***

🞏 Counselor or Clinician - ***SWITCH TO CLINICIAN ONLY VERSION***
🞏 Clinical Supervisor and Counselor/Clinician ***- CONTINUE HERE WITH SUPERVISOR + CLINICIAN VERSION***

🞏 Other, please describe: ____________________(e.g., administrator, grant manager) ***- SWITCH TO SUPERVISOR VERSION***

5. Did you participate in any SAMHSA/CSAT-funded A-CRA training initiatives in your state? Yes or No?

*[IF NEEDED, PROMPT AS RELEVANT: Our records do/do not indicate that you received training in A-CRA as part of a SAMHSA CSAT grant under the [initiative] in [grants years]. (IF KNOWN) In your state, I believe this initiative was called [NAME]. So would it be accurate to say you did/did not participate in your state’s A-CRA training initiative?]*

🞏 No – ***SKIP TO QUESTION 6***🞏 Yes – ***CONTINUE***

**IF QUESTION 5 = Yes**

5A. Between what dates did you work on the project? Your best guess is fine.

Enter Start Date (MM/YY)

Enter End Date (MM/YY)

6. What role(s) did you serve while A-CRA was being delivered at your agency? ***[MARK ALL THAT APPLY]***

🞏 A-CRA Therapist
🞏 A-CRA Supervisor
🞏 Other (Please describe: ____________________________________________________)
🞏 No Role in delivering ACRA

7. Have you received any A-CRA certifications? This means that you completed all training requirements (such as coaching and submission of recorded sessions) and received written documentation of the certification.

**[MARK ALL THAT APPLY]**

🞏 First-level clinician certification (passed 9 A-CRA procedures)
🞏 Full clinician certification (passed all 19 A-CRA procedures)
🞏 Supervisor certification
🞏 A-CRA-TAY (Transitional Age Youth) certification

🞏 No, never certified in A-CRA

*[IF NEEDED, PROMPT:  A-CRA supervisor certification is different from A-CRA certification.  It refers to being certified to provide A-CRA clinical supervision].*

8. What do you think of A-CRA as a treatment for youth?

9. When did you/your agency stop delivering A-CRA? If you do not know the exact date, please give your best estimate.

Month/Year:______ /______

10. What were the main reasons you/your agency stopped delivering A-CRA?

11. What would have increased your desire to continue delivering A-CRA?

12. What would have increased your ability to continue delivering A-CRA?

13. Does your program currently offer any different types of treatment for youth with substance use disorders besides A-CRA?

🞏 No – ***SKIP TO QUESTION 15***
🞏 Yes - ***CONTINUE***
🞏 Don’t Know – ***SKIP TO QUESTION 15***

IF QUESTION 13= YES

14. What are those treatments called?

*[IF NEEDED, PROMPT: Examples of other treatments include Motivational Enhancement Therapy (MET), Cognitive Behavioral Therapy (CBT), Multidimensional Family Therapy (MDFT), 12-step facilitation, and supportive counseling as well as medication treatments like Suboxone or Naltrexone.*

*OR you can just describe the treatment. Is it outpatient? How long does it last? # of sessions/days/months?]*

15. Over the past 6 months, approximately how many youth received substance use treatment at your agency?

_______0-999

16. Approximately how many youth received A-CRA over the past 6 months?

_______0-999

IF QUESTION 16 = 0

16A. Approximately how many youth received A-CRA during the six-month period, right before A-CRA treatment delivery ended?

17. Currently, how many clinicians at your agency treat youth with substance use problems?

_______0-999

18. Currently, how many of the clinicians at your agency have received an A-CRA certification?

*[IF NEEDED, PROMPT: By certified, we mean they completed all training requirements – such as coaching and submission of recorded sessions – and received written documentation of certification. This includes first-level, full, transitional age youth, and supervisor certifications.]*

_______0-999

IF QUESTION 18 = 0

18A. Approximately how many clinicians at your agency had received an A-CRA certification during the six-month period, right before A-CRA treatment delivery ended?

19. Next, I’d like to ask you about your agency’s A-CRA clinical supervision support **during the last six months it was delivered.** Was your agency able to maintain a Clinical Supervisor focused on A-CRA?

🞏 No – ***SKIP TO QUESTION 20***
🞏 Yes
🞏 Don’t Know

**IF QUESTION 19= YES**

19A. Did your agency support Clinical Supervisor and Counselor time for supervision?

🞏 No
🞏 Yes
🞏 Don’t Know

**IF QUESTION 19= YES**

19B. Did your agency support the Clinical Supervisor to listen to recorded therapy sessions and provide individualized feedback to counselors?

🞏 No
🞏 Yes
🞏 Don’t Know

20. Tell me about any ways in which your organization collaborates with other organizations – especially anything related to A-CRA. *[IF NEEDED, PROMPT: For example, you may have engaged in training or support activities with your state substance use services authority and/or Chestnut Health Systems, or have partnered with universities or research institutions on research related to A-CRA].*

21. Was there any planning done to ensure resources were available to continue A-CRA beyond the initial funding period? By resources, we mean things needed to support A-CRA. For example, this could include things like money, staff, supervision, training, and A-CRA manuals.

[*IF NEEDED, PROMPT: By strategic planning, we mean the process by which an organization defines its strategy or direction and makes decisions about how to allocate resources to pursue the strategy.]*

22. Can you please describe any policies that supported A-CRA delivery? These could include external policies, such as funding source and billing rules that support A-CRA, as well as internal policies at your organization.

*[IF NEEDED, PROMPT: By policies, we mean either organizational, state, national, or local policies. Here are some examples:*

*-State/county/health insurance co requires that we use an evidence based treatment (EBT) and A-CRA is an EBT*

*-Our agency’s mission emphasizes the involvement of the family, A-CRA does that]*

23. What about policies (external or internal) that interfered with A-CRA delivery?

*[IF NEEDED, PROMPT:  By policies, we mean either organizational, state, national, or local policies. Here are some examples:*

*-if a state decided to require that substance use clinics deliver a different treatment (other than A-CRA), this policy might interfere with the delivery of A-CRA.*

*- if certain insurance plans stopped reimbursing for A-CRA or the full number of sessions, this policy would interfere with A-CRA delivery.]*

24. Did A-CRA meet the needs of the populations you serve? How so or why not?

25. Can you tell me about any pressure your organization experienced to continue delivering A-CRA or to discontinue its use? *[IF NEEDED, PROMPT:  Pressure to deliver A-CRA could come from a variety of sources. For example, organizations might experience pressure if there is some financial incentive to deliver A-CRA. If a state department of health decides its mission is to deliver evidence based treatment for substance use, this could also be seen as pressure.]*

26. Would you say staff were supportive or reluctant to use A-CRA? Can you give me an example of what people have said or done to indicate their supportiveness or reluctance toward A-CRA?

27. Next, I would like to know what factors helped efforts to implement and sustain A-CRA in your organization. Please let me know if these factors were important in your organization, and if yes, how so.

*[PROBE AS NEEDED: What factors were most helpful in that domain? Can you give me some examples? Were there important changes in which factors were most helpful once funding ended?]*

27A. Would you say characteristics of A-CRA helped efforts to implement and sustain A-CRA in your organization?  Yes or No?

*[IF NEEDED: for example, treatment content, structure of sessions, etc.]*

🞏 No
🞏 Yes

IF YES, please describe: When was that factor most impactful? Was it during the CSAT grant funding period, after the CSAT funding ended, or throughout?

27B. Would you say characteristics of key individuals helped efforts to implement and sustain A-CRA in your organization?  Yes or No?

*[IF NEEDED: “key individuals” are people who had a major positive impact on A-CRA implementation; could be within or outside of your treatment organization]*

🞏 No
🞏 Yes

IF YES, please describe: When was that factor most impactful? Was it during the CSAT grant funding period, after the CSAT funding ended, or throughout?

27C. Would you say client perspectives on A-CRA helped efforts to implement and sustain A-CRA in your organization?  Yes or No?

*[IF NEEDED: for example, if clients found A-CRA acceptable, appropriate, feasible, etc.]*

🞏 No
🞏 Yes

IF YES, please describe: When was that factor most impactful? Was it during the CSAT grant funding period, after the CSAT funding ended, or throughout?

27D. Would you say helpful factors within your organization helped efforts to implement and sustain A-CRA in your organization?  Yes or No?

*[IF NEEDED: for example, organizational leadership, staffing patterns, scheduling appointments, etc.]*

🞏 No
🞏 Yes

IF YES, please describe: When was that factor most impactful? Was it during the CSAT grant funding period, after the CSAT funding ended, or throughout?

27E. Would you say helpful factors outside your organization helped efforts to implement and sustain A-CRA in your organization?  Yes or No?

*[IF NEEDED: for example, state leadership, federal support, community factors]*

🞏 No
🞏 Yes

IF YES, please describe: When was that factor most impactful? Was it during the CSAT grant funding period, after the CSAT funding ended, or throughout?

27F. Would you say partnerships with other organizations helped efforts to implement and sustain A-CRA in your organization?  Yes or No?

🞏 No
🞏 Yes

IF YES, please describe: When was that factor most impactful? Was it during the CSAT grant funding period, after the CSAT funding ended, or throughout?

27G. Would you say funding helped efforts to implement and sustain A-CRA in your organization?  Yes or No?

🞏 No
🞏 Yes

IF YES, please describe: When was that factor most impactful? Was it during the CSAT grant funding period, after the CSAT funding ended, or throughout?

27H. Were there other helpful factors during or after the grant period?

🞏 No
🞏 Yes

IF YES, please describe: When was that factor most impactful? Was it during the CSAT grant funding period, after the CSAT funding ended, or throughout?

28. Now, I’m going ask how the same six categories of factors may have included barriers that hindered your efforts to implement and sustain A-CRA in your organization. Please let me know if these factors were important at your organization, and if yes, how so.

*[PROBE AS NEEDED: What factors were the biggest barriers in that domain? Can you give me some examples? Were there important changes in which factors were the biggest barriers once funding ended?]*

28A. Would you say characteristics of A-CRA hindered efforts to implement and sustain A-CRA in your organization? Yes or No?

*[IF NEEDED: for example, treatment content, structure of sessions, etc.]*

🞏 No
🞏 Yes

IF YES, please describe: When was that factor most impactful? Was it during the CSAT grant funding period, after the CSAT funding ended, or throughout?

28B. Would you say challenges with key individuals hindered efforts to implement and sustain A-CRA in your organization? Yes or No?

*[IF NEEDED: “key individuals” are people who presented major challenges for A-CRA implementation; could be within or outside of your treatment organization]*

🞏 No
🞏 Yes

IF YES, please describe: When was that factor most impactful? Was it during the CSAT grant funding period, after the CSAT funding ended, or throughout?

28C. Would you say client perspectives on A-CRA hindered efforts to implement and sustain A-CRA in your organization? Yes or No?

*[IF NEEDED: for example, if clients found A-CRA acceptable, appropriate, feasible, etc.]*

🞏 No
🞏 Yes

IF YES, please describe: When was that factor most impactful? Was it during the CSAT grant funding period, after the CSAT funding ended, or throughout?

28D. Would you say challenges within your organization hindered efforts to implement and sustain A-CRA in your organization? Yes or No?

*[IF NEEDED: for example, organizational leadership, staffing patterns, scheduling appointments, etc.]*

🞏 No
🞏 Yes

IF YES, please describe: When was that factor most impactful? Was it during the CSAT grant funding period, after the CSAT funding ended, or throughout?

28E. Would you say challenges outside your organization hindered efforts to implement and sustain A-CRA in your organization? Yes or No?

*[IF NEEDED: for example, state leadership, federal support, community factors]*

🞏 No
🞏 Yes

IF YES, please describe: When was that factor most impactful? Was it during the CSAT grant funding period, after the CSAT funding ended, or throughout?

28F. Would you say issues in partnerships with other organizations hindered efforts to implement and sustain A-CRA in your organization? Yes or No?

🞏 No
🞏 Yes

IF YES, please describe: When was that factor most impactful? Was it during the CSAT grant funding period, after the CSAT funding ended, or throughout?

28G. Would you say funding hindered efforts to implement and sustain A-CRA in your organization? Yes or No?

🞏 No
🞏 Yes

IF YES, please describe: When was that factor most impactful? Was it during the CSAT grant funding period, after the CSAT funding ended, or throughout?

28H. Were there other barriers during or after the grant period?

🞏 No
🞏 Yes

IF YES, please describe: When was that factor most impactful? Was it during the CSAT grant funding period, after the CSAT funding ended, or throughout?

29. How many staff supervise clinicians who treat youth with substance use problems?

______0-999

30. Did you supervise clinicians delivering A-CRA at your agency?

🞏 No
🞏 Yes
🞏 Don’t Know

31. How many others at your agency supervised clinicians delivering A-CRA?

______0-99

32. Have you personally certified any clinicians in A-CRA?

🞏 No –***SKIP TO QUESTION 49***
🞏 Yes - ***CONTINUE***

*[IF NEEDED, PROMPT: By certified, we mean they completed all training requirements – such as coaching and submission of recorded sessions – and received written documentation of certification. Respond “yes” if you have personally certified anyone with the first-level, full, transitional age youth, and/or supervisor certifications.]*

**IF QUESTION 32 = Yes**

33. You mentioned that you personally had certified one or more clinicians in A-CRA at your organization. Can you please describe the certification process to me?

The clinician was asked to:

🞏 Read the A-CRA manual

🞏 Take an online A-CRA research course

🞏 Pass an A-CRA quiz with a score of 80% or higher

🞏 Attend a Chestnut or Robert J. Meyers A-CRA initial training OR attend an in-house training

🞏 Participate in regular coaching calls with Chestnut or regular supervision with in-house Clinical Supervisor (respondent) during certification (regular= @ every other week)

🞏 Regularly record therapy sessions for in-house Clinical Supervisor (respondent) review/Chestnut’s review (regular= at least some sessions weekly))

🞏 Demonstrate competency in General Clinical Skills on the DSRs

🞏 Demonstrate competency in the following A-CRA procedures:

Functional Analysis of Use

Functional Analysis of Pro-social behavior

Happiness Scale

Treatment Plan/Goals of Counseling

Communication Skills

Problem Solving Skills

Adolescent-Caregiver Relationship Skills, and

Homework based of 3 or better on all components of a given procedure using the A-CRA rating manual?

If in-house training was provided, did it:

🞏 Include didactic information about A-CRA procedures?

🞏 Modeling or review of audio recordings of procedures that were well done

🞏 The opportunity to role play procedures

🞏 Other, please explain_____________________________________

**Next, I'm going to ask you a series of questions about how the certification process worked in your agency. Please respond by saying "True," "False," or "don't know" if you are unsure.**

34. When I decided to pass a clinician on a procedure it was based on ratings of 1 or more on every component of a procedure. Remember that each component of a procedure is rated on a 1 to 5 scale.

🞏 False
🞏 True
🞏 Don’t Know

35. Communication skills was a procedure that people had to pass to attain certification.

🞏 False
🞏 True
🞏 Don’t Know

36. I reviewed recorded sessions during the certification process.

🞏 False
🞏 True
🞏 Don’t Know

37. I was required to sit in on sessions with clinicians during the certification process.

🞏 False
🞏 True
🞏 Don’t Know

38. Clinicians recorded one or two of their sessions.

🞏 False
🞏 True
🞏 Don’t Know

39. I referred to the A-CRA rating manual when rating session recordings.

🞏 False
🞏 True
🞏 Don’t Know

40. Clinicians were required to take a knowledge test as part of the certification process.

🞏 False
🞏 True
🞏 Don’t Know

41. Clinicians were not required to pass General Clinical Skills as part of the certification process.

🞏 False
🞏 True
🞏 Don’t Know

42. Each clinician had a certification workbook.

🞏 False
🞏 True
🞏 Don’t Know

43. I completed the A-CRA checklist when I was listening to a recorded session during or after the certification process.

🞏 False
🞏 True
🞏 Don’t Know

44. Clinicians were required to read the A-CRA manual during the training process.

🞏 False
🞏 True
🞏 Don’t Know

45. Time was set aside for training clinicians in A-CRA

🞏 False
🞏 True
🞏 Don’t Know

46. During training clinicians were required to practice procedures with role-plays.

🞏 False
🞏 True
🞏 Don’t Know

47. Adolescent-Caregiver Relationship Skills was one of the procedures for basic certification.

🞏 False
🞏 True
🞏 Don’t Know

48. It didn’t matter if clinicians showed competency in all of the additional procedures as well.

🞏 False
🞏 True
🞏 Don’t Know

49. On average, approximately how many session recordings did clinicians at your site complete to reach first-level certification (pass first 9 A-CRA procedures)? Your best guess is fine.

______0-99

50. Have you ever had a copy of the A-CRA manual? *[IF NEEDED, PROMPT: If you have access to a shared copy of the manual through your organization, electronically, etc. that counts as having a copy.]*

🞏 No - ***SKIP TO QUESTION 54***🞏 Yes - ***CONTINUE***

**IF QUESTION 50 = Yes**

51. Please think about the six-month period, right before A-CRA treatment delivery ended. How often did you use the manual?

*[IF NEEDED, PROMPT: By use, we mean a reference to the manual. It could mean a quick review of key session content in preparation for a session, or a thorough read-through in order to master the material.]*

🞏 Never
🞏 A few times per year, or less
🞏 About once a month
🞏 A few times per month
🞏 Weekly
🞏 Daily

**IF QUESTION 50 = Yes**

52. Do you still use your manual?

*[IF NEEDED, PROMPT: By use, we mean a reference to the manual. It could mean a quick review of key session content in preparation for a session, or a thorough read-through in order to master the material.]*

🞏 No – ***SKIP TO QUESTION 54***🞏 Yes - ***CONTINUE***

**IF QUESTION 52 = Yes**

53. How often do you use your manual now?

*[IF NEEDED, PROMPT: By use, we mean a reference to the manual. It could mean a quick review of key session content in preparation for a session, or a thorough read-through in order to master the material.]*

🞏 Never
🞏 A few times per year, or less
🞏 About once a month
🞏 A few times per month
🞏 Weekly
🞏 Daily

**For the next few questions, please think about the six-month period, right before A-CRA treatment delivery ended.**

54. How often did you provide group clinical supervision?

*[IF NEEDED, PROMPT:  This includes any supervision you provided; not supervision you received. Our focus is on A-CRA supervision but you can include any supervision where A-CRA would be discussed – it does not need to be exclusively supervision for A-CRA cases.]*

🞏 Never
🞏 Once a week
🞏 Every two weeks
🞏 Monthly
🞏 Other, please describe how often:___________________________________

55. Again, please think about the six-month period, right before A-CRA treatment delivery ended. How often did you provide individual clinical supervision?

*[IF NEEDED, PROMPT:  This includes any supervision you provided; not supervision you received. Our focus is on A-CRA supervision but you can include any supervision where A-CRA would be discussed – it does not need to be exclusively supervision for A-CRA cases.]*

🞏 Never
🞏 Once a week
🞏 Every two weeks
🞏 Monthly
🞏 Other, please describe how often:___________________________________

**IF QUESTION 54 = Never AND QUESTION 55= Never, SKIP TO QUESTION 66**

**Thinking about the six-month period, right before A-CRA treatment delivery ended, which of the following happened during supervision sessions?**

56. My supervisor asked how my week had gone.

🞏 No
🞏 Yes

57. (During the six-month period, right before A-CRA treatment delivery ended), my supervisor discussed agency paperwork requirements.

🞏 No
🞏 Yes

58. (During the six-month period, right before A-CRA treatment delivery ended), my supervisor reviewed my A-CRA case review report.

🞏 No
🞏 Yes

59. (During the six-month period, right before A-CRA treatment delivery ended), my supervisor asked me if I had any problem cases.

🞏 No
🞏 Yes

60. (During the six-month period, right before A-CRA treatment delivery ended), my supervisor reviewed a recorded session with me and told me what I did well.

🞏 No
🞏 Yes

61. (During the six-month period, right before A-CRA treatment delivery ended), my supervisor reviewed a recorded session with me and gave me suggestions about how I could improve my treatment delivery

🞏 No
🞏 Yes

62. During the six-month period, right before A-CRA treatment delivery ended, my supervisor observed a live session and told me what I did well.

🞏 No
🞏 Yes

63. (During the six-month period, right before A-CRA treatment delivery ended), my supervisor observed a live session and gave me suggestions about how I could improve my treatment delivery

🞏 No
🞏 Yes

64. (During the six-month period, right before A-CRA treatment delivery ended), my supervisor asked me about my personal problems.

🞏 No
🞏 Yes

65. During the six-month period, right before A-CRA treatment delivery ended, my supervisor role played with me the correct way to do a procedure.

🞏 No
🞏 Yes

**Now I’d like to ask you the same questions, but now please think about the current practices in the adolescent treatment program.**

66. How often do you provide group clinical supervision?

*[IF NEEDED, PROMPT:  This includes any supervision you provided; not supervision you received. Our focus is on A-CRA supervision but you can include any supervision where A-CRA would be discussed – it does not need to be exclusively supervision for A-CRA cases.]*

🞏 Never
🞏 Once a week
🞏 Every two weeks
🞏 Monthly
🞏 Other, please describe how often:___________________________________

67. How often do you provide individual clinical supervision?

*[IF NEEDED, PROMPT:  This includes any supervision you provided; not supervision you received. Our focus is on A-CRA supervision but you can include any supervision where A-CRA would be discussed – it does not need to be exclusively supervision for A-CRA cases.]*

🞏 Never
🞏 Once a week
🞏 Every two weeks
🞏 Monthly
🞏 Other, please describe how often:___________________________________

***IF QUESTION 66 = Never AND QUESTION 67= Never, SKIP TO QUESTION 77***

**Thinking about the current practices in your adolescent treatment program, which of the following happens during supervision sessions?**

68. My supervisor asks me how my week has gone.

🞏 No
🞏 Yes

69. My supervisor discusses agency paperwork requirements.

🞏 No
🞏 Yes

70. My supervisor asks me if I have any problem cases.

🞏 No
🞏 Yes

71. My supervisor reviews a recorded session with me and tells me what I have done well.

🞏 No
🞏 Yes

72. My supervisor reviews a recorded session with me and gives me suggestions about how I could improve my treatment delivery.

🞏 No
🞏 Yes

73. My supervisor observes a live session and tells me what I have done well.

🞏 No
🞏 Yes

74. My supervisor observes a live session and gives me suggestions about how I could improve my treatment delivery.

🞏 No
🞏 Yes

75. My supervisor asks me about my personal problems.

🞏 No
🞏 Yes

76. My supervisor role plays with me the correct way to deliver therapy.

🞏 No
🞏 Yes

77. During the six month period, right before A-CRA treatment delivery ended, how many sessions did you tell the client receiving A-CRA they would have?

_______0-99

🞏 LESS THAN 12
🞏 12 OR MORE

78. During the six month period, right before A-CRA treatment delivery ended, how many weeks did you tell the client receiving A-CRA the treatment would take?

_______0-99

🞏 LESS THAN 12 WEEKS
🞏 12 WEEKS OR MORE

79. How has the COVID-19 pandemic affected substance use treatment services at your organization?

80. When the pandemic began in March 2020, was your organization still delivering A-CRA?

🞏 No – ***SKIP TO QUESTION 83***
🞏 Yes – ***CONTINUE***

🞏 Don’t know – ***SKIP TO QUESTION 83***

**IF QUESTION 80= YES**

81. What have been the most significant changes affecting your A-CRA program in response to COVID-19?

*[PROBE AS NEEDED: Changes could include things like…*

*…A-CRA was discontinued due to COVID-related changes*

*…Changes in referrals to A-CRA*

*…Changes in how A-CRA assessment, treatment, or case management services are delivered*

*…Changes in staffing*

*…Remote work*

*…Telehealth service delivery*

*Were there other changes? If so, please describe: ]*

**IF QUESTION 80 = YES**

82. Have there been changes made in response to COVID-19 that have been beneficial, and will those changes continue beyond the pandemic?

*[PROBE AS NEEDED: These may be some of the changes you already described, or may be different. Changes could include things like…*

*…Changes in referrals to A-CRA*

*…Changes in how A-CRA assessment, treatment, or case management services are delivered*

*…Changes in staffing*

*…Remote work*

*…Telehealth service delivery*

*Were there other beneficial changes? If so, please describe: ]*

**OK, we are almost done. Next, I want to ask you about your general impressions regarding the SAMHSA CSAT-funded A-CRA project and then have a few questions about you.**

83. If you had a chance to participate in a SAMHSA CSAT project again, would you consider it?

🞏 No
🞏 Yes

84. Why/why not?

85. Is there anything you would change about the SAMHSA CSAT project in order to improve the sustainment of A-CRA at your agency?

86. Is there anything else you would like to share related to the topic of sustaining A-CRA at your agency?

[TURN OFF RECORDER]

That's all the interview questions I have. Thank you for providing this information. We will soon be sending you the link to the web survey in order to provide a more complete picture of the treatment offered to youth at your organization and about organizational and clinical support. We will send it within a few days, and will send your $50 Amazon e-gift card as soon as the survey is complete.

[IF PARTICIPANT WILL BE INTERVIEWED AGAIN, complete the tracking module beginning on the final page]

***SUPERVISOR INTERVIEW VERBAL CONSENT***

(READ PRIOR TO BEGINNING INTERVIEW/RECORDING)

Hi [SUPERVISOR NAME],

This is [INTERVIEWER NAME] calling from RAND to speak with you about the youth substance use treatment program where you work and any experiences you have with implementing the Adolescent Community Reinforcement Approach (A-CRA) treatment model. Is this still a good time to complete your interview? To ensure confidentiality, make sure you are in a private, secure location for completing the interview.

>IF NO, RESCHEDULE.

>IF YES, great.

We would like a clinician and/or clinical supervisor for youth substance use treatment to answer these questions. Ideally, it should be someone knowledgeable about your A-CRA program. Are you the right person at your organization to participate in this opportunity?

>IF NO: May I please have the contact information of the best contact(s) for youth substance use treatment? [RECORD CONTACT INFORMATION TO FOLLOW-UP]

>IF YES, thanks for confirming.

Before we begin, let me assure you that your responses to these questions will be held in strict confidence. In collaboration with Chestnut Health Systems, we are requesting interviews and surveys from clinicians and supervisors from nearly 20 states that received A-CRA training from Chestnut Health, as part of SAMHSA Center of Substance Abuse Treatment (CSAT) grants received by state substance use services authorities. We will use information from the interviews and surveys to understand how different CSAT funding models influence the sustainability of A-CRA delivery after funding ends. We will also examine information provided by state organizations that received the CSAT grants and data already collected from prior CSAT grantee organizations that implemented A-CRA. We will not attribute comments to specific individuals or programs in any of our reports or publications. Your responses will not be shared with your organization or with SAMHSA.

Today’s interview will last up to 45 minutes. Afterwards, we will ask you to complete a 30-minute online survey on your own to give us a more complete picture. You will receive a $50 Amazon electronic gift card upon completion of the web survey as a thank you for your participation.

Your participation in this discussion and the survey is entirely voluntary. We would like to have your responses to all of the questions. However, if you’re uncomfortable with any question we can skip it, and you can stop the interview at any time. There are no right or wrong answers – we are interested in your perspectives and experiences. Finally, we would like to audio-record the interview to ensure that we capture everything that is said. We will destroy the recording once we confirm we have captured everything in our de-identified notes and transcripts. However, you can still participate in the interview even if you do not give permission to audio-record.

If you have any questions or want to discuss the project further at any time, you may always contact us at 360-578-2911 or at pham@rand.org. Furthermore, if you have questions about your rights as a research participant or need to report a research-related injury or concern, you can contact RAND's Human Subjects Protection Committee toll-free at (866) 697-5620 or email [hspcinfo@​rand.org](mailto:hspcinfo@​rand.org)​. If you contact the Committee, please reference Study #2020-N0887.

- **Do you have any questions?**
- **Are you willing to take part in this discussion?**

>IF NO: That is not a problem, thank you for your time.

>IF YES, great.

- **Is it ok with you if we audio-tape this discussion?**

>IF NO: That is not a problem. I can take notes while we’re talking so I don’t miss anything important, though that means we might proceed through the interview more slowly than usual. I could also arrange for a colleague to take notes during the interview. [RESCHEDULE IF NEEDED]

>IF YES, perfect, let’s get started.

- **[IF UNKNOWN] Based on the background information we’ve received, I am not sure whether your organization currently delivers A-CRA. Can you confirm whether or not A-CRA is delivered by clinicians at your organization?**

>IF NO: BEGIN NON-SUSTAINER INTERVIEW

>IF YES: BEGIN SUSTAINER INTERVIEW

**Sustainer Interview – SUPERVISOR**

1. Based on the background information we’ve received, it sounds like your agency currently delivers the Adolescent Community Reinforcement Approach or A-CRA. Is this correct?

🞏 No – ***SWITCH TO NON-SUSTAINER INTERVIEW***🞏 Yes - ***CONTINUE***

2. Tell me a little bit about the substance use services at your agency. What services are available? What types of clients do you serve? (e.g., age range, services for specific substances).

3. What youth age range do you use A-CRA with at your agency? Throughout this interview, by “youth” we typically mean ages 12 through 17 (or through age 24 if your A-CRA training included young adults).

4. What is your primary professional role(s) at your clinic/site?

🞏 Clinical Supervisor – ***CONTINUE***

🞏 Counselor or Clinician - ***SWITCH TO CLINICIAN ONLY VERSION***
🞏 Clinical Supervisor and Counselor/Clinician ***– SWITCH TO SUPERVISOR + CLINICIAN VERSION***

🞏 Other, please describe: ____________________(e.g., administrator, grant manager) ***- CONTINUE***

5. Did you participate in any SAMHSA/CSAT-funded A-CRA training initiatives in your state? Yes or No?

*[IF NEEDED, PROMPT AS RELEVANT: Our records do/do not indicate that you received training in A-CRA as part of a SAMHSA CSAT grant under the [initiative] in [grants years]. (IF KNOWN) In your state, I believe this initiative was called [NAME]. So would it be accurate to say you did/did not participate in your state’s A-CRA training initiative?]*

🞏 No – ***SKIP TO QUESTION 6***🞏 Yes – ***CONTINUE***

**IF QUESTION 5 = Yes**

5A. Between what dates did you work on the project? Your best guess is fine.

Enter Start Date (MM/YY)

Enter End Date (MM/YY)

6. What role(s) have you served in delivering A-CRA? ***[MARK ALL THAT APPLY]***

🞏 A-CRA Therapist
🞏 A-CRA Clinical Supervisor
🞏 Other (Please describe: ____________________________________________________)
🞏 No Role in delivering ACRA

7. Have you received any A-CRA certifications? This means that you completed all training requirements (such as coaching and submission of recorded sessions) and received written documentation of the certification.

***[MARK ALL THAT APPLY]***

*[IF NEEDED, PROMPT:  A-CRA supervisor certification is different from A-CRA certification.  It refers to being certified to provide A-CRA clinical supervision].*

🞏 First-level clinician certification (passed 9 A-CRA procedures)
🞏 Full clinician certification (passed all 19 A-CRA procedures)
🞏 Supervisor certification
🞏 A-CRA-TAY (Transitional Age Youth) certification

🞏 No, never certified in A-CRA

8. What do you think of A-CRA as a treatment for youth? Please note that throughout this interview, by “youth” we mean the population served during your SAMHSA CSAT grant periods.

9. Does your program currently offer any different types of treatment for youth with substance use disorders besides A-CRA?

🞏 No – ***SKIP TO QUESTION 11***
🞏 Yes - ***CONTINUE***
🞏 Don’t Know – ***SKIP TO QUESTION 11***

**IF QUESTION 9= YES**

10. What are those treatments called?

*[IF NEEDED, PROMPT: Examples of other treatments include Motivational Enhancement Therapy (MET), Cognitive Behavioral Therapy (CBT), Multidimensional Family Therapy (MDFT), 12-step facilitation, and supportive counseling as well as medication treatments like Suboxone or Naltrexone.*

*OR you can just describe the treatment. Is it outpatient? How long does it last? # of sessions/days/months?]*

11. Over the past 6 months, approximately how many youth received substance use treatment at your agency?

_______0-999

12. Approximately how many youth received A-CRA over the past 6 months?

_______0-999

13. Currently, how many clinicians at your agency treat youth with substance use problems?

_______0-999

14. Currently, how many of the clinicians at your agency have received an A-CRA certification?

*[IF NEEDED, PROMPT: By certified, we mean they completed all training requirements – such as coaching and submission of recorded sessions – and received written documentation of certification. This includes first-level, full, transitional age youth, and supervisor certifications.]*

_______0-999

15. How many clinicians deliver A-CRA at your agency?

_______0-999

16. Next, I’d like to ask you about your agency’s plan to continue A-CRA clinical supervision support.

Does your agency plan to maintain a Clinical Supervisor focused on A-CRA?

🞏 No – ***SKIP TO QUESTION 17***
🞏 Yes
🞏 Don’t Know

**IF QUESTION 16= YES**

16A. Does your agency plan to support Clinical Supervisor and Counselor time for supervision?

🞏 No
🞏 Yes
🞏 Don’t Know

**IF QUESTION 16= YES**

16B. Does your agency plan to support the Clinical Supervisor to listen to recorded therapy sessions and provide individualized feedback to counselors?

🞏 No
🞏 Yes
🞏 Don’t Know

17. Tell me about any ways in which your organization collaborates with other organizations to sustain A-CRA.

*[IF NEEDED, PROMPT: For example, you may have engaged in training or support activities with your state substance use services authority and/or Chestnut Health Systems, or have partnered with universities or research institutions on research related to A-CRA].*

18. Tell me about any planning that has been done to ensure resources are available to continue A-CRA. By resources, we mean things needed to support A-CRA. For example, this could include things like money, staff, supervision, training, and A-CRA manuals.

[*IF NEEDED, PROMPT: By strategic planning, we mean the process by which an organization defines its strategy or direction and makes decisions about how to allocate resources to pursue the strategy.]*

19. Can you please describe any policies that support A-CRA delivery? These could include external policies, such as funding source and billing rules that support A-CRA, as well as internal policies at your organization.

*[IF NEEDED, PROMPT: By policies, we mean either organizational, state, national, or local policies. Here are some examples:*

*-State/county/health insurance co requires that we use an evidence based treatment (EBT) and A-CRA is an EBT*

*-Our agency’s mission emphasizes involvement of the family, A-CRA does that]*

20. What about policies (external or internal) that interfere with A-CRA delivery?

*[IF NEEDED, PROMPT:  By policies, we mean either organizational, state, national, or local policies. Here are some examples:*

*-if a state decided to require that substance use clinics deliver a different treatment (other than A-CRA), this policy might interfere with the delivery of A-CRA.*

*- if certain insurance plans stopped reimbursing for A-CRA or the full number of sessions, this policy would interfere with A-CRA delivery.]*

21. Does A-CRA meet the needs of the populations you serve? How so or why not?

22. Can you tell me about any pressure your organization experienced to continue delivering A-CRA or to discontinue its use?

*[IF NEEDED, PROMPT: Pressure to deliver A-CRA could come from a variety of sources. For ex., organizations might experience pressure if there is some financial incentive to deliver A-CRA. If a state department of health decides its mission is to deliver evidence based treatment for substance use, this could also be seen as pressure.]*

23. Would you say staff are supportive or reluctant to use A-CRA? Can you give me an example of what people have said or done to indicate their supportiveness or reluctance toward A-CRA?

24. Next, I would like to know what factors helped efforts to implement and sustain A-CRA in your organization. Please let me know if these factors were important in your organization, and if yes, how so.

*[PROBE AS NEEDED: What factors were most helpful in that domain? Can you give me some examples? Were there important changes in which factors were most helpful once funding ended?]*

24A. Would you say characteristics of A-CRA helped efforts to implement and sustain A-CRA in your organization?  Yes or No?

*[IF NEEDED: for example, treatment content, structure of sessions, etc.]*

🞏 No
🞏 Yes

IF YES, please describe: When was that factor most impactful? Was it during the CSAT grant funding period, after the CSAT funding ended, or throughout?

24B. Would you say characteristics of key individuals helped efforts to implement and sustain A-CRA in your organization?  Yes or No?

*[IF NEEDED: “key individuals” are people who had a major positive impact on A-CRA implementation; could be within or outside of your treatment organization]*

🞏 No
🞏 Yes

IF YES, please describe: When was that factor most impactful? Was it during the CSAT grant funding period, after the CSAT funding ended, or throughout?

24C. Would you say client perspectives on A-CRA helped efforts to implement and sustain A-CRA in your organization?  Yes or No?

*[IF NEEDED: for example, if clients found A-CRA acceptable, appropriate, feasible, etc.]*

🞏 No
🞏 Yes

IF YES, please describe: When was that factor most impactful? Was it during the CSAT grant funding period, after the CSAT funding ended, or throughout?

24D. Would you say helpful factors within your organization helped efforts to implement and sustain A-CRA in your organization?  Yes or No?

*[IF NEEDED: for example, organizational leadership, staffing patterns, scheduling appointments, etc.]*

🞏 No
🞏 Yes

IF YES, please describe: When was that factor most impactful? Was it during the CSAT grant funding period, after the CSAT funding ended, or throughout?

24E. Would you say helpful factors outside your organization helped efforts to implement and sustain A-CRA in your organization?  Yes or No?

*[IF NEEDED: for example, state leadership, federal support, community factors]*

🞏 No
🞏 Yes

IF YES, please describe: When was that factor most impactful? Was it during the CSAT grant funding period, after the CSAT funding ended, or throughout?

24F. Would you say partnerships with other organizations helped efforts to implement and sustain A-CRA in your organization?  Yes or No?

🞏 No
🞏 Yes

IF YES, please describe: When was that factor most impactful? Was it during the CSAT grant funding period, after the CSAT funding ended, or throughout?

24G. Would you say funding helped efforts to implement and sustain A-CRA in your organization?  Yes or No?

🞏 No
🞏 Yes

IF YES, please describe: When was that factor most impactful? Was it during the CSAT grant funding period, after the CSAT funding ended, or throughout?

24H. Were there other helpful factors during or after the grant period?

🞏 No
🞏 Yes

IF YES, please describe: When was that factor most impactful? Was it during the CSAT grant funding period, after the CSAT funding ended, or throughout?

25. Now, I’m going ask how the same six categories of factors may have included barriers that hindered efforts to implement and sustain A-CRA in your organization. Please let me know if these factors were important in your organization, and if yes, how so.

*[PROBE AS NEEDED: What factors were the biggest barriers in that domain? Can you give me some examples? Were there important changes in which factors were the biggest barriers once funding ended?]*

25A. Would you say characteristics of A-CRA hindered efforts to implement and sustain A-CRA in your organization? Yes or No?

*[IF NEEDED: for example, treatment content, structure of sessions, etc.]*

🞏 No
🞏 Yes

IF YES, please describe: When was that factor most impactful? Was it during the CSAT grant funding period, after the CSAT funding ended, or throughout?

25B. Would you say challenges with key individuals hindered efforts to implement and sustain A-CRA in your organization? Yes or No?

*[IF NEEDED: “key individuals” are people who presented major challenges for A-CRA implementation; could be within or outside of your treatment organization]*

🞏 No
🞏 Yes

IF YES, please describe: When was that factor most impactful? Was it during the CSAT grant funding period, after the CSAT funding ended, or throughout?

25C. Would you say client perspectives on A-CRA hindered efforts to implement and sustain A-CRA in your organization? Yes or No?

*[IF NEEDED: for example, if clients found A-CRA acceptable, appropriate, feasible, etc.]*

🞏 No
🞏 Yes

IF YES, please describe: When was that factor most impactful? Was it during the CSAT grant funding period, after the CSAT funding ended, or throughout?

25D. Would you say challenges within your organization hindered efforts to implement and sustain A-CRA in your organization? Yes or No?

*[IF NEEDED: for example, organizational leadership, staffing patterns, scheduling appointments, etc.]*

🞏 No
🞏 Yes

IF YES, please describe: When was that factor most impactful? Was it during the CSAT grant funding period, after the CSAT funding ended, or throughout?

25E. Would you say challenges outside your organization hindered efforts to implement and sustain A-CRA in your organization? Yes or No?

*[IF NEEDED: for example, state leadership, federal support, community factors]*

🞏 No
🞏 Yes

IF YES, please describe: When was that factor most impactful? Was it during the CSAT grant funding period, after the CSAT funding ended, or throughout?

25F. Would you say issues in partnerships with other organizations hindered efforts to implement and sustain A-CRA in your organization? Yes or No?

🞏 No
🞏 Yes

IF YES, please describe: When was that factor most impactful? Was it during the CSAT grant funding period, after the CSAT funding ended, or throughout?

25G. Would you say funding hindered efforts to implement and sustain A-CRA in your organization? Yes or No?

🞏 No
🞏 Yes

IF YES, please describe: When was that factor most impactful? Was it during the CSAT grant funding period, after the CSAT funding ended, or throughout?

25H. Were there other barriers during or after the grant period?

🞏 No
🞏 Yes

IF YES, please describe: When was that factor most impactful? Was it during the CSAT grant funding period, after the CSAT funding ended, or throughout?

26. How many staff supervise clinicians who treat youth with substance use problems?

______0-999

27. Do you supervise clinicians who deliver A-CRA?

🞏 No
🞏 Yes
🞏 Don’t Know

28. How many others at your agency supervise clinicians delivering A-CRA?

______0-99

29. Have you personally certified any clinicians in A-CRA?

🞏 No –***SKIP TO QUESTION 46***
🞏 Yes - ***CONTINUE***

*[IF NEEDED, PROMPT: By certified, we mean they completed all training requirements – such as coaching and submission of recorded sessions – and received written documentation of certification. Respond “yes” if you have personally certified anyone with the first-level, full, transitional age youth, and/or supervisor certifications.]*

**IF QUESTION 29 = YES**

30. You mentioned that you personally had certified one or more clinicians in A-CRA at your organization. Can you please describe the certification process to me?

The clinician was asked to:

🞏 Read the A-CRA manual

🞏 Take an online A-CRA research course

🞏 Pass an A-CRA quiz with a score of 80% or higher

🞏 Attend a Chestnut or Robert J. Meyers A-CRA initial training OR attend an in-house training

🞏 Participate in regular coaching calls with Chestnut or regular supervision with in-house Clinical Supervisor (respondent) during certification (regular= @ every other week)

🞏 Regularly record therapy sessions for in-house Clinical Supervisor (respondent) review/Chestnut’s review (regular= at least some sessions weekly))

🞏 Demonstrate competency in General Clinical Skills on the DSRs

🞏 Demonstrate competency in the following A-CRA procedures:

Functional Analysis of Use

Functional Analysis of Pro-social behavior

Happiness Scale

Treatment Plan/Goals of Counseling

Communication Skills

Problem Solving Skills

Adolescent-Caregiver Relationship Skills, and

Homework based of 3 or better on all components of a given procedure using the A-CRA rating manual?

If in-house training was provided, did it:

🞏 Include didactic information about A-CRA procedures?

🞏 Modeling or review of audio recordings of procedures that were well done

🞏 The opportunity to role play procedures

🞏 Other, please explain_____________________________________

**Next, I'm going to ask you a series of questions about how the certification process currently works in your agency. Please respond by saying "True," "False," or "don't know" if you are unsure.**

31. When I decide to pass a clinician on a procedure it is based on ratings of 1 or more on every component of a procedure. Remember that each component of a procedure is rated on a 1 to 5 scale.

🞏 False
🞏 True
🞏 Don’t Know

32. Communication skills is a procedure that people have to pass to attain certification.

🞏 False
🞏 True
🞏 Don’t Know

33. I review recorded sessions during the certification process.

🞏 False
🞏 True
🞏 Don’t Know

34. I am required to sit in sessions with clinicians during the certification process.

🞏 False
🞏 True
🞏 Don’t Know

35. Clinicians record one or two of their sessions.

🞏 False
🞏 True
🞏 Don’t Know

36. I refer to the A-CRA rating manual when rating session recordings.

🞏 False
🞏 True
🞏 Don’t Know

37. Clinicians are required to take a knowledge test as part of the certification process.

🞏 False
🞏 True
🞏 Don’t Know

38. Clinicians are not required to pass General Clinical Skills as part of the certification process.

🞏 False
🞏 True
🞏 Don’t Know

39. Each clinician has a certification workbook.

🞏 False
🞏 True
🞏 Don’t Know

40. I complete the A-CRA checklist when I am listening to a recorded session during or after the certification process.

🞏 False
🞏 True
🞏 Don’t Know

41. Clinicians are required to read the A-CRA manual during the training process.

🞏 False
🞏 True
🞏 Don’t Know

42. Time is set aside for training clinicians in A-CRA

🞏 False
🞏 True
🞏 Don’t Know

43. During training clinicians are required to practice procedures with role-plays.

🞏 False
🞏 True
🞏 Don’t Know

44. Adolescent-Caregiver Relationship Skills is one of the procedures for basic certification.

🞏 False
🞏 True
🞏 Don’t Know

45. It doesn't matter if clinicians show competency in all of the additional procedures as well.

🞏 False
🞏 True
🞏 Don’t Know

46. On average, approximately how many session recordings do clinicians at your site complete to reach first-level certification (pass first 9 A-CRA procedures)? Your best guess is fine.

______0-99

47. Do you have a copy of the A-CRA manual? *[IF NEEDED, PROMPT: If you have access to a shared copy of the manual through your organization, electronically, etc. that counts as having a copy.]*

🞏 No – ***SKIP TO QUESTION 51***
🞏 Yes – ***CONTINUE***

48. In the past six-months, how often would you say you used the manual?

*[IF NEEDED, PROMPT: By use, we mean a reference to the manual. It could mean a quick review of key session content in preparation for a session, or a thorough read-through in order to master the material.]*

🞏 Never
🞏 A few times per year, or less
🞏 About once a month
🞏 A few times per month
🞏 Weekly
🞏 Daily

49. In the past six-months, how often do you provide group clinical supervision?

*[IF NEEDED, PROMPT:  This includes any supervision you provided; not supervision you received. Our focus is on A-CRA supervision but you can include any supervision where A-CRA would be discussed – it does not need to be exclusively supervision for A-CRA cases.]*

o      Never
o      Once a week
o      Every two weeks
o      Monthly
o      Other, please describe how often:___________________________________

50. In the past six-months, how often do you provide individual clinical supervision?

*[IF NEEDED, PROMPT:  This includes any supervision you provided; not supervision you received. Our focus is on A-CRA supervision but you can include any supervision where A-CRA would be discussed – it does not need to be exclusively supervision for A-CRA cases.]*

o      Never
o      Once a week
o      Every two weeks
o      Monthly
o      Other, please describe how often:___________________________________

51. How many new clinicians have been trained in A-CRA in the past six-months in the organization?

______0-999

52. How many new clinicians have been trained in A-CRA in the past six-months at a Chestnut or Robert J. Meyers training?

______0-999

**[IF ANSWER TO QUESTION 51 > or = to ANSWER TO QUESTION 52 CONTINUE; OTHERWISE, SKIP TO QUESTION 55]**

**IF ANSWER TO QUESTION 51 >= ANSWER TO QUESTION 52**

53. Are there training agendas for your trainings?

🞏 No ***SKIP TO QUESTION 55***
🞏 Yes ***CONTINUE***

**IF QUESTION 53=Yes AND ANSWER TO QUESTION 51>0 AND ANSWER TO QUESTION 51 >= ANSWER TO QUESTION 52**

54. Will you please send me (email, mail, fax) a copy of the agenda?

🞏 No
🞏 Yes

**Currently, when you are introducing A-CRA to a new client…**

55. How many sessions do you tell the client receiving A-CRA they will have?

_______0-99

🞏 LESS THAN 12
🞏 12 OR MORE

56. How many weeks do you tell the client receiving A-CRA the treatment will take?

_______0-99

🞏 LESS THAN 12 WEEKS
🞏 12 WEEKS OR MORE

57. How has the COVID-19 pandemic affected substance use treatment services at your organization?

58. How has it affected the ability of your organization to sustain A-CRA services?

59. What have been the most significant changes affecting your A-CRA program in response to COVID-19?

*[PROBE AS NEEDED: Changes could include things like…*

*…Changes in referrals to A-CRA*

*…Changes in how A-CRA assessment, treatment, or case management services are delivered*

*…Changes in staffing*

*…Remote work*

*…Telehealth service delivery*

*Were there other changes? If so, please describe: ]*

60. Have there been changes made in response to COVID-19 that have been beneficial, and will those changes continue beyond the pandemic?

*[PROBE AS NEEDED: These may be some of the changes you already described, or may be different. Changes could include things like…*

*…Changes in referrals to A-CRA*

*…Changes in how A-CRA assessment, treatment, or case management services are delivered*

*…Changes in staffing*

*…Remote work*

*…Telehealth service delivery*

*Were there other beneficial changes? If so, please describe: ]*

**OK, we are almost done. Next, I want to ask you about your general impressions regarding the SAMHSA CSAT-funded A-CRA project and then have a few questions about you.**

61. If you had a chance to participate in a SAMHSA CSAT project again, would you consider it?

🞏 No
🞏 Yes

62. Why/why not?

63. Is there anything you would change about the SAMHSA CSAT project in order to improve the sustainment of A-CRA at your agency?

64. Is there anything else you would like to share related to the topic of sustaining A-CRA at your agency?

[TURN OFF RECORDER]

That's all the interview questions I have. Thank you for providing this information. We will soon be sending you the link to the web survey in order to provide a more complete picture of the treatment offered to youth at your organization and about organizational and clinical support. We will send it within a few days, and will send your $50 Amazon e-gift card as soon as the survey is complete.

[IF PARTICIPANT WILL BE INTERVIEWED AGAIN, complete the tracking module beginning on the final page]

**Non-Sustainer Interview –SUPERVISOR**

1. Based on the background information we’ve received, it sounds like your agency currently does not deliver the Adolescent Community Reinforcement Approach or A-CRA anymore. Is this correct?

🞏 No – **SWITCH TO SUSTAINER INTERVIEW**
🞏 Yes - ***CONTINUE***

2. Tell me a little bit about the substance use services at your agency. What services are available? What types of clients do you serve? (e.g., age range, services for specific substances).

3. What youth age range do you use A-CRA with at your agency? Throughout this interview, by “youth” we typically mean ages 12 through 17 (or through age 24 if your A-CRA training included young adults).

4. What is your primary professional role(s) at your clinic/site?

🞏 Clinical Supervisor – ***CONTINUE***

🞏 Counselor or Clinician - ***SWITCH TO CLINICIAN ONLY VERSION***
🞏 Clinical Supervisor and Counselor/Clinician ***– SWITCH TO SUPERVISOR + CLINICIAN VERSION***

🞏 Other, please describe: ____________________(e.g., administrator, grant manager) ***– CONTINUE***

5. Did you participate in any SAMHSA/CSAT-funded A-CRA training initiatives in your state? Yes or No?

*[IF NEEDED, PROMPT AS RELEVANT: Our records do/do not indicate that you received training in A-CRA as part of a SAMHSA CSAT grant under the [initiative] in [grants years]. (IF KNOWN) In your state, I believe this initiative was called [NAME]. So would it be accurate to say you did/did not participate in your state’s A-CRA training initiative?]*

🞏 No – ***SKIP TO QUESTION 6***🞏 Yes – ***CONTINUE***

**IF QUESTION 5 = Yes**

5A. Between what dates did you work on the project? Your best guess is fine.

Enter Start Date (MM/YY)

Enter End Date (MM/YY)

6. What role(s) did you serve while A-CRA was being delivered at your agency? ***[MARK ALL THAT APPLY]***

🞏 A-CRA Therapist
🞏 A-CRA Supervisor
🞏 Other (Please describe: ____________________________________________________)
🞏 No Role in delivering ACRA

7. Have you received any A-CRA certifications? This means that you completed all training requirements (such as coaching and submission of recorded sessions) and received written documentation of the certification.

**[MARK ALL THAT APPLY]**

🞏 First-level clinician certification (passed 9 A-CRA procedures)
🞏 Full clinician certification (passed all 19 A-CRA procedures)
🞏 Supervisor certification
🞏 A-CRA-TAY (Transitional Age Youth) certification

🞏 No, never certified in A-CRA

*[IF NEEDED, PROMPT:  A-CRA supervisor certification is different from A-CRA certification.  It refers to being certified to provide A-CRA clinical supervision].*

8. What do you think of A-CRA as a treatment for youth?

9. When did you/your agency stop delivering A-CRA? If you do not know the exact date, please give your best estimate.

Month/Year:______ /______

10. What were the main reasons you/your agency stopped delivering A-CRA?

11. What would have increased your desire to continue delivering A-CRA?

12. What would have increased your ability to continue delivering A-CRA?

13. Does your program currently offer any different types of treatment for youth with substance use disorders besides A-CRA?

🞏 No – ***SKIP TO QUESTION 15***
🞏 Yes - ***CONTINUE***
🞏 Don’t Know – ***SKIP TO QUESTION 15***

IF QUESTION 13= YES

14. What are those treatments called?

*[IF NEEDED, PROMPT: Examples of other treatments include Motivational Enhancement Therapy (MET), Cognitive Behavioral Therapy (CBT), Multidimensional Family Therapy (MDFT), 12-step facilitation, and supportive counseling as well as medication treatments like Suboxone or Naltrexone.*

*OR you can just describe the treatment. Is it outpatient? How long does it last? # of sessions/days/months?]*

15. Over the past 6 months, approximately how many youth received substance use treatment at your agency?

_______0-999

16. Approximately how many youth received A-CRA over the past 6 months?

_______0-999

IF QUESTION 16 = 0

16A. Approximately how many youth received A-CRA during the six-month period, right before A-CRA treatment delivery ended?

17. Currently, how many clinicians at your agency treat youth with substance use problems?

_______0-999

18. Currently, how many of the clinicians at your agency have received an A-CRA certification?

*[IF NEEDED, PROMPT: By certified, we mean they completed all training requirements – such as coaching and submission of recorded sessions – and received written documentation of certification. This includes first-level, full, transitional age youth, and supervisor certifications.]*

_______0-999

IF QUESTION 18 = 0

18A. Approximately how many clinicians at your agency had received an A-CRA certification during the six-month period, right before A-CRA treatment delivery ended?

19. Next, I’d like to ask you about your agency’s A-CRA clinical supervision support **during the last six months it was delivered.** Was your agency able to maintain a Clinical Supervisor focused on A-CRA?

🞏 No – ***SKIP TO QUESTION 20***
🞏 Yes
🞏 Don’t Know

**IF QUESTION 19= YES**

19A. Did your agency support Clinical Supervisor and Counselor time for supervision?

🞏 No
🞏 Yes
🞏 Don’t Know

**IF QUESTION 19= YES**

19B. Did your agency support the Clinical Supervisor to listen to recorded therapy sessions and provide individualized feedback to counselors?

🞏 No
🞏 Yes
🞏 Don’t Know

20. Tell me about any ways in which your organization collaborates with other organizations – especially anything related to A-CRA. *[IF NEEDED, PROMPT: For example, you may have engaged in training or support activities with your state substance use services authority and/or Chestnut Health Systems, or have partnered with universities or research institutions on research related to A-CRA].*

21. Was there any planning done to ensure resources were available to continue A-CRA beyond the initial funding period? By resources, we mean things needed to support A-CRA. For example, this could include things like money, staff, supervision, training, and A-CRA manuals.

*[IF NEEDED, PROMPT: By strategic planning, we mean the process by which an organization defines its strategy or direction and makes decisions about how to allocate resources to pursue the strategy.]*

22. Can you please describe any policies that supported A-CRA delivery? These could include external policies, such as funding source and billing rules that support A-CRA, as well as internal policies at your organization.

*[IF NEEDED, PROMPT: By policies, we mean either organizational, state, national, or local policies. Here are some examples:*

*-State/county/health insurance co requires that we use an evidence based treatment (EBT) and A-CRA is an EBT*

*-Our agency’s mission emphasizes the involvement of the family, A-CRA does that]*

23. What about policies (external or internal) that interfered with A-CRA delivery?

*[IF NEEDED, PROMPT:  By policies, we mean either organizational, state, national, or local policies. Here are some examples:*

*-if a state decided to require that substance use clinics deliver a different treatment (other than A-CRA), this policy might interfere with the delivery of A-CRA.*

*- if certain insurance plans stopped reimbursing for A-CRA or the full number of sessions, this policy would interfere with A-CRA delivery.]*

24. Did A-CRA meet the needs of the populations you serve? How so or why not?

25. Can you tell me about any pressure your organization experienced to continue delivering A-CRA or to discontinue its use? *[IF NEEDED, PROMPT:  Pressure to deliver A-CRA could come from a variety of sources. For example, organizations might experience pressure if there is some financial incentive to deliver A-CRA. If a state department of health decides its mission is to deliver evidence based treatment for substance use, this could also be seen as pressure.]*

26. Would you say staff were supportive or reluctant to use A-CRA? Can you give me an example of what people have said or done to indicate their supportiveness or reluctance toward A-CRA?

27. Next, I would like to know what factors helped efforts to implement and sustain A-CRA in your organization. Please let me know if these factors were important in your organization, and if yes, how so.

*[PROBE AS NEEDED: What factors were most helpful in that domain? Can you give me some examples? Were there important changes in which factors were most helpful once funding ended?]*

27A. Would you say characteristics of A-CRA helped efforts to implement and sustain A-CRA in your organization?  Yes or No?

*[IF NEEDED: for example, treatment content, structure of sessions, etc.]*

🞏 No
🞏 Yes

IF YES, please describe: When was that factor most impactful? Was it during the CSAT grant funding period, after the CSAT funding ended, or throughout?

27B. Would you say characteristics of key individuals helped efforts to implement and sustain A-CRA in your organization?  Yes or No?

*[IF NEEDED: “key individuals” are people who had a major positive impact on A-CRA implementation; could be within or outside of your treatment organization]*

🞏 No
🞏 Yes

IF YES, please describe: When was that factor most impactful? Was it during the CSAT grant funding period, after the CSAT funding ended, or throughout?

27C. Would you say client perspectives on A-CRA helped efforts to implement and sustain A-CRA in your organization?  Yes or No?

*[IF NEEDED: for example, if clients found A-CRA acceptable, appropriate, feasible, etc.]*

🞏 No
🞏 Yes

IF YES, please describe: When was that factor most impactful? Was it during the CSAT grant funding period, after the CSAT funding ended, or throughout?

27D. Would you say helpful factors within your organization helped efforts to implement and sustain A-CRA in your organization?  Yes or No?

*[IF NEEDED: for example, organizational leadership, staffing patterns, scheduling appointments, etc.]*

🞏 No
🞏 Yes

IF YES, please describe: When was that factor most impactful? Was it during the CSAT grant funding period, after the CSAT funding ended, or throughout?

27E. Would you say helpful factors outside your organization helped efforts to implement and sustain A-CRA in your organization?  Yes or No?

*[IF NEEDED: for example, state leadership, federal support, community factors]*

🞏 No
🞏 Yes

IF YES, please describe: When was that factor most impactful? Was it during the CSAT grant funding period, after the CSAT funding ended, or throughout?

27F. Would you say partnerships with other organizations helped efforts to implement and sustain A-CRA in your organization?  Yes or No?

🞏 No
🞏 Yes

IF YES, please describe: When was that factor most impactful? Was it during the CSAT grant funding period, after the CSAT funding ended, or throughout?

27G. Would you say funding helped efforts to implement and sustain A-CRA in your organization?  Yes or No?

🞏 No
🞏 Yes

IF YES, please describe: When was that factor most impactful? Was it during the CSAT grant funding period, after the CSAT funding ended, or throughout?

27H. Were there other helpful factors during or after the grant period?

🞏 No
🞏 Yes

IF YES, please describe: When was that factor most impactful? Was it during the CSAT grant funding period, after the CSAT funding ended, or throughout?

28. Now, I’m going ask how the same six categories of factors may have included barriers that hindered efforts to implement and sustain A-CRA in your organization. Please let me know if these factors were important in your organization, and if yes, how so.

*[PROBE AS NEEDED: What factors were the biggest barriers in that domain? Can you give me some examples? Were there important changes in which factors were the biggest barriers once funding ended?]*

28A. Would you say characteristics of A-CRA hindered efforts to implement and sustain A-CRA in your organization? Yes or No?

*[IF NEEDED: for example, treatment content, structure of sessions, etc.]*

🞏 No
🞏 Yes

IF YES, please describe: When was that factor most impactful? Was it during the CSAT grant funding period, after the CSAT funding ended, or throughout?

28B. Would you say challenges with key individuals hindered efforts to implement and sustain A-CRA in your organization? Yes or No?

*[IF NEEDED: “key individuals” are people who presented major challenges for A-CRA implementation; could be within or outside of your treatment organization]*

🞏 No
🞏 Yes

IF YES, please describe: When was that factor most impactful? Was it during the CSAT grant funding period, after the CSAT funding ended, or throughout?

28C. Would you say client perspectives on A-CRA hindered efforts to implement and sustain A-CRA in your organization? Yes or No?

*[IF NEEDED: for example, if clients found A-CRA acceptable, appropriate, feasible, etc.]*

🞏 No
🞏 Yes

IF YES, please describe: When was that factor most impactful? Was it during the CSAT grant funding period, after the CSAT funding ended, or throughout?

28D. Would you say challenges within your organization hindered efforts to implement and sustain A-CRA in your organization? Yes or No?

*[IF NEEDED: for example, organizational leadership, staffing patterns, scheduling appointments, etc.]*

🞏 No
🞏 Yes

IF YES, please describe: When was that factor most impactful? Was it during the CSAT grant funding period, after the CSAT funding ended, or throughout?

28E. Would you say challenges outside your organization hindered efforts to implement and sustain A-CRA in your organization? Yes or No?

*[IF NEEDED: for example, state leadership, federal support, community factors]*

🞏 No
🞏 Yes

IF YES, please describe: When was that factor most impactful? Was it during the CSAT grant funding period, after the CSAT funding ended, or throughout?

28F. Would you say issues in partnerships with other organizations hindered efforts to implement and sustain A-CRA in your organization? Yes or No?

🞏 No
🞏 Yes

IF YES, please describe: When was that factor most impactful? Was it during the CSAT grant funding period, after the CSAT funding ended, or throughout?

28G. Would you say funding hindered efforts to implement and sustain A-CRA in your organization? Yes or No?

🞏 No
🞏 Yes

IF YES, please describe: When was that factor most impactful? Was it during the CSAT grant funding period, after the CSAT funding ended, or throughout?

28H. Were there other barriers during or after the grant period?

🞏 No
🞏 Yes

IF YES, please describe: When was that factor most impactful? Was it during the CSAT grant funding period, after the CSAT funding ended, or throughout?

29. How many staff supervise clinicians who treat youth with substance use problems?

______0-999

30. Did you supervise clinicians delivering A-CRA at your agency?

🞏 No
🞏 Yes
🞏 Don’t Know

31. How many others at your agency supervised clinicians delivering A-CRA?

______0-99

32. Have you personally certified any clinicians in A-CRA?

🞏 No –***SKIP TO QUESTION 49***
🞏 Yes - ***CONTINUE***

*[IF NEEDED, PROMPT: By certified, we mean they completed all training requirements – such as coaching and submission of recorded sessions – and received written documentation of certification. Respond “yes” if you have personally certified anyone with the first-level, full, transitional age youth, and/or supervisor certifications.]*

**IF QUESTION 32 = Yes**

33. You mentioned that you personally had certified one or more clinicians in A-CRA at your organization. Can you please describe the certification process to me?

The clinician was asked to:

🞏 Read the A-CRA manual

🞏 Take an online A-CRA research course

🞏 Pass an A-CRA quiz with a score of 80% or higher

🞏 Attend a Chestnut or Robert J. Meyers A-CRA initial training OR attend an in-house training

🞏 Participate in regular coaching calls with Chestnut or regular supervision with in-house Clinical Supervisor (respondent) during certification (regular= @ every other week)

🞏 Regularly record therapy sessions for in-house Clinical Supervisor (respondent) review/Chestnut’s review (regular= at least some sessions weekly))

🞏 Demonstrate competency in General Clinical Skills on the DSRs

🞏 Demonstrate competency in the following A-CRA procedures:

Functional Analysis of Use

Functional Analysis of Pro-social behavior

Happiness Scale

Treatment Plan/Goals of Counseling

Communication Skills

Problem Solving Skills

Adolescent-Caregiver Relationship Skills, and

Homework based of 3 or better on all components of a given procedure using the A-CRA rating manual?

If in-house training was provided, did it:

🞏 Include didactic information about A-CRA procedures?

🞏 Modeling or review of audio recordings of procedures that were well done

🞏 The opportunity to role play procedures

🞏 Other, please explain_____________________________________

**Next, I'm going to ask you a series of questions about how the certification process worked in your agency. Please respond by saying "True," "False," or "don't know" if you are unsure.**

34. When I decided to pass a clinician on a procedure it was based on ratings of 1 or more on every component of a procedure. Remember that each component of a procedure is rated on a 1 to 5 scale.

🞏 False
🞏 True
🞏 Don’t Know

35. Communication skills was a procedure that people had to pass to attain certification.

🞏 False
🞏 True
🞏 Don’t Know

36. I reviewed recorded sessions during the certification process.

🞏 False
🞏 True
🞏 Don’t Know

37. I was required to sit in on sessions with clinicians during the certification process.

🞏 False
🞏 True
🞏 Don’t Know

38. Clinicians recorded one or two of their sessions.

🞏 False
🞏 True
🞏 Don’t Know

39. I referred to the A-CRA rating manual when rating session recordings.

🞏 False
🞏 True
🞏 Don’t Know

40. Clinicians were required to take a knowledge test as part of the certification process.

🞏 False
🞏 True
🞏 Don’t Know

41. Clinicians were not required to pass General Clinical Skills as part of the certification process.

🞏 False
🞏 True
🞏 Don’t Know

42. Each clinician had a certification workbook.

🞏 False
🞏 True
🞏 Don’t Know

43. I completed the A-CRA checklist when I was listening to a recorded session during or after the certification process.

🞏 False
🞏 True
🞏 Don’t Know

44. Clinicians were required to read the A-CRA manual during the training process.

🞏 False
🞏 True
🞏 Don’t Know

45. Time was set aside for training clinicians in A-CRA

🞏 False
🞏 True
🞏 Don’t Know

46. During training clinicians were required to practice procedures with role-plays.

🞏 False
🞏 True
🞏 Don’t Know

47. Adolescent-Caregiver Relationship Skills was one of the procedures for basic certification.

🞏 False
🞏 True
🞏 Don’t Know

48. It didn’t matter if clinicians showed competency in all of the additional procedures as well.

🞏 False
🞏 True
🞏 Don’t Know

49. On average, approximately how many session recordings did clinicians at your site complete to reach first-level certification (pass first 9 A-CRA procedures)? Your best guess is fine.

______0-99

50. Have you ever had a copy of the A-CRA manual? *[IF NEEDED, PROMPT: If you have access to a shared copy of the manual through your organization, electronically, etc. that counts as having a copy.]*

🞏 No - ***SKIP TO QUESTION 56***🞏 Yes - ***CONTINUE***

**IF QUESTION 50= Yes**

51. Please think about the six-month period, right before A-CRA treatment delivery ended. How often did you use the manual?

*[IF NEEDED, PROMPT: By use, we mean a reference to the manual. It could mean a quick review of key session content in preparation for a session, or a thorough read-through in order to master the material.]*

🞏 Never
🞏 A few times per year, or less
🞏 About once a month
🞏 A few times per month
🞏 Weekly
🞏 Daily

**IF QUESTION 50 = Yes**

52. Do you still use your manual?

*[IF NEEDED, PROMPT: By use, we mean a reference to the manual. It could mean a quick review of key session content in preparation for a session, or a thorough read-through in order to master the material.]*

🞏 No - ***SKIP TO QUESTION 56***

🞏 Yes - ***CONTINUE***

**IF QUESTION 52 = Yes**

53. How often do you use your manual now?

*[IF NEEDED, PROMPT: By use, we mean a reference to the manual. It could mean a quick review of key session content in preparation for a session, or a thorough read-through in order to master the material.]*

🞏 Never
🞏 A few times per year, or less
🞏 About once a month
🞏 A few times per month
🞏 Weekly
🞏 Daily

**For the next few questions, please think about the six-month period, right before A-CRA treatment delivery ended.**

54. How often do you provide group clinical supervision?

*[IF NEEDED, PROMPT:  This includes any supervision you provided; not supervision you received. Our focus is on A-CRA supervision but you can include any supervision where A-CRA would be discussed – it does not need to be exclusively supervision for A-CRA cases.]*

o      Never
o      Once a week
o      Every two weeks
o      Monthly
o      Other, please describe how often:___________________________________

55. How often do you provide individual clinical supervision?

*[IF NEEDED, PROMPT:  This includes any supervision you provided; not supervision you received. Our focus is on A-CRA supervision but you can include any supervision where A-CRA would be discussed – it does not need to be exclusively supervision for A-CRA cases.]*

o      Never
o      Once a week
o      Every two weeks
o      Monthly
o      Other, please describe how often:___________________________________

56. During the six month period, right before A-CRA treatment delivery ended, how many sessions did you tell the client receiving A-CRA they would have?

_______0-99

🞏 LESS THAN 12
🞏 12 OR MORE

57. During the six month period, right before A-CRA treatment delivery ended, how many weeks did you tell the client receiving A-CRA the treatment would take?

_______0-99

🞏 LESS THAN 12 WEEKS
🞏 12 WEEKS OR MORE

56. How has the COVID-19 pandemic affected substance use treatment services at your organization?

58. When the pandemic began in March 2020, was your organization still delivering A-CRA?

🞏 No – ***SKIP TO QUESTION 61***
🞏 Yes – ***CONTINUE***

🞏 Don’t know – ***SKIP TO QUESTION 61***

**IF QUESTION 58 = YES**

59. What have been the most significant changes affecting your A-CRA program in response to COVID-19?

*[PROBE AS NEEDED: Changes could include things like…*

*…A-CRA was discontinued due to COVID-related changes*

*…Changes in referrals to A-CRA*

*…Changes in how A-CRA assessment, treatment, or case management services are delivered*

*…Changes in staffing*

*…Remote work*

*…Telehealth service delivery*

*Were there other changes? If so, please describe: ]*

**IF QUESTION 58 = YES**

60. Have there been changes made in response to COVID-19 that have been beneficial, and will those changes continue beyond the pandemic?

*[PROBE AS NEEDED: These may be some of the changes you already described, or may be different. Changes could include things like…*

*…Changes in referrals to A-CRA*

*…Changes in how A-CRA assessment, treatment, or case management services are delivered*

*…Changes in staffing*

*…Remote work*

*…Telehealth service delivery*

*Were there other beneficial changes? If so, please describe: ]*

**OK, we are almost done. Next, I want to ask you about your general impressions regarding the SAMHSA CSAT-funded A-CRA project and then have a few questions about you.**

61. If you had a chance to participate in a SAMHSA CSAT project again, would you consider it?

🞏 No
🞏 Yes

62. Why/why not?

63. Is there anything you would change about the SAMHSA CSAT project in order to improve the sustainment of A-CRA at your agency?

64. Is there anything else you would like to share related to the topic of sustaining A-CRA at your agency?

[TURN OFF RECORDER]

That's all the interview questions I have. Thank you for providing this information. We will soon be sending you the link to the web survey in order to provide a more complete picture of the treatment offered to youth at your organization and about organizational and clinical support. We will send it within a few days, and will send your $50 Amazon e-gift card as soon as the survey is complete.

[IF PARTICIPANT WILL BE INTERVIEWED AGAIN, complete the tracking module beginning on the final page]

**A-CRA Financing Project Tracking Module**

Now I need to get some information to help us contact you next year. As I mentioned at the beginning, we would like to interview you every year for up to 4 years. This information, like your responses to all questions in the interview, is completely confidential. It will only be used to contact you about the project; your identity will not be linked to your interview or survey responses in any way.

IF NECESSARY: *You are very important to this study. Your experiences over the years will help us to understand how to better sustain evidence-based treatments, like A-CRA and to ultimately improve the quality of care for youth with substance use problems. When we contact you, you can decide if you want to participate in the next interview and survey.*

Q1. Just in case we are unable to reach you next year, can you confirm your work e-mail address and phone number?

Work Email:

Work Phone:

Q2. How about a personal e-mail address or cell phone number? We will only use your personal email or cell phone number if we are having trouble reaching you at your work e-mail address/phone.

Personal/Alternate Email:

Personal/Alternate Cell Phone:

Q3. Is there anyone we can contact if that may help us reach you? This could be a co-worker, a family member, a friend – whoever you think is best. What is their name and contact information? We will only contact them if we are having trouble reaching you.

Contact Name:

Contact relationship to you:

Contact Phone:

Contact Email:

Q4. Is there anything else we should keep in mind when contacting you for future interviews?

Again, thank you for your participation in this important study.

Q5. INTERVIEW NOTES FIELD:
