# Supplementary material for: Comparing two federal financing strategies on penetration and sustainment of the adolescent community reinforcement approach for substance use disorders: protocol for a mixed-method study
Source: Implement Sci Commun. 2022 May 13;3:51. doi: 10.1186/s43058-022-00298-y (PMC9099033; doi:10.1186/s43058-022-00298-y)
Supplement: Supplementary file 4 — Additional file 4. Analysis plan for A-CRA Financing. [file 43058_2022_298_MOESM4_ESM.docx]

**Additional File 4: Detailed analysis plan with equations for Aims 1 and 2**

***Aim 1a: A-CRA penetration outcomes*.** Aim 1a will evaluate the overall impact of grant type on penetration outcomes (% clinicians certified) at the end of the grant period. Let $Y_{s,i}^{P}$ denote a penetration outcome at site *i* in state *s*. Let $I\left\{ grant type \right\}$ be a binary indicator (1 for state-focused, 0 for organization-focused). The hierarchical model is:

$Y_{s,i}^{P}=\alpha+\mu\times I\left\{ grant type \right\}+X_{s,i}\beta+Z_{s}\lambda+e_{s,i}$ **(1)**

In this model, α is the common intercept for mathematical purposes, $X_{s,i}\beta$ represents the effect of organization-level covariates, and $Z_{s}\lambda$ represents the effect of state-level covariates. The last term $e_{s,i}$is the measurement error. The effect of the grant type is parametrized by $\mu$. Testing H_0_:$\mu=0$ suffices to examine the effect. The *z*-test is automatically performed as a part of model fitting. We will examine whether all controlled covariates are needed by a suitable variable selection procedure such as LASSO or the Bayes information criterion.

***Aim 1b: Moderators of A-CRA penetration*.** We will perform the analysis of hypothesized moderators based on model (1). Let *T_s,i_* denote moderators (baseline outer-context reimbursement or inner-context leadership), both at site *i* in state *s.* Analysis is based on model (2), which expands model (1) by including the two-way interaction term $\eta T_{s,i}\times I\left\{ grant type \right\}$:

$Y_{s,i}^{P}=\alpha^{(2)}+\Delta T_{s,i}+\mu^{(2)}\times I\left\{ grant type \right\}+\eta T_{s,i}{\times I\left\{ grant type \right\}+X}_{s,i}\beta^{(2)}+Z_{s}\lambda^{(2)}+e_{s,i}^{(2)}$ **(2)**

Testing H_0_: $\eta=0$ suffices to examine the hypothesized moderator.

***Aim 2a: A-CRA sustainment outcomes*.** We will apply the pattern-mixture model to the longitudinal sustainment outcomes. Let $Y_{s,i,t}^{S}$ denote sustainment as measured in site *i* at time *t* (e.g., # months since funding ended, 0 ≤ *t*) in state *s*. First, sites are grouped into distinct patterns according to their sustainment status and data availability; for example, sites that sustained A-CRA at similar levels (e.g., high vs. low) or with similar activities (e.g., staffing vs. supervision elements) may be grouped into the same pattern. The model has the following general form:

$Y_{s,i,t}^{S}=\alpha^{(p)}+\lambda^{\left( p \right)}\left( t \right)+\mu\times I\left\{ grant type \right\}+X_{s,i}\beta^{(p)}+Z_{s}\lambda^{\left( p \right)}+\alpha_{i}+e_{s,i,t}$ **(3)**

The average effect of grant type across all patterns is represented by $\mu$ and tested during model fitting. The${}^{\left( p \right)}$ term denotes the pattern-specific effects of intercept $\alpha^{(p)}$, controlled covariates $X_{s,i}\beta^{(p)}+Z_{s}\lambda^{\left( p \right)}$, and a time effect $\lambda^{\left( p \right)}\left( t \right)$ which may be linear or quadratic (depending on the goodness of fit to the data). The${}^{\left( p \right)}$ term allows distinct parameters for each pattern, which prevents bias in the estimation of $\mu$. The last two terms are a site-level random effect *a_i_* (which accounts for autocorrelation within sites) and measurement error.

***Aim 2b: Mediators and moderators of A-CRA penetration outcomes*.** In this model, *M_s,i,t_* denotes the time-varying mediators (outer-context reimbursement or funding stability) and *T_s,i,t_* denotes the time-varying moderators (inner-context leadership or sustainment capacity), all at site *i* and time *t* in state *s.* The mediation analysis is expressed by the structural equation consisting of models (3) and (4) (5) below:

$M_{s,i,t}=\alpha^{(p,0)}+ \lambda^{\left( p,0 \right)}(t)+\mu^{(p,0)}\times I\left\{ grant type \right\}+X_{s,i}\beta^{(p,0)}+Z_{s}\lambda^{(p,0)}+a_{i}^{(0)}+e_{s,i,t}^{(0)}$ **(4)**

$Y_{s,i,t}^{S}=\alpha^{(p,1)}+\lambda^{\left( p,1 \right)}\left( t \right)+\theta M_{s,i,t}+\mu^{(1)}\times I\left\{ grant type \right\}+X_{s,i}\beta^{(p,1)}+Z_{s}\lambda^{(p,1)}+a_{i}^{(1)}+e_{s,i,t}^{(1)}$ **(5)**

The superscript denotes a specific covariate effect in a model equation (0 or 1) and in a pattern *p*. First, we will test $\alpha^{(p,0)}$ to confirm the relation between the mediator and the grant type. Next, we will test the significance of $\theta\mu^{(p,0)}$ and $\mu^{(1)}$ separately**,** which represents the indirect and direct effect of grant type.

The moderation analysis is carried out by adding an interaction term to model (6), similar to Aim 1b. The moderation effect is represented by the interaction $\eta T_{s,i,t}\times I\left\{ grant type \right\}$ and tested by H_0_: $\eta=0$:

$Y_{s,i,t}^{S}=\alpha^{(p,2)}+\Delta T_{s,i,t}+\mu^{(2)}\times I\left\{ grant type \right\}+\eta T_{s,i,t}\times I\left\{ grant type \right\}+X_{s,i}\beta^{(p,2)}+Z_{s}\lambda^{\left( p,2 \right)}$

$+a_{i}^{(2)}+e_{s,i,t}^{(2)}$ **(6)**
